# Comparison of a theoretically driven cognitive therapy (the Feeling Safe Programme) with befriending for the treatment of persistent persecutory delusions: a parallel, single-blind, randomised controlled trial

**DOI:** 10.1016/S2215-0366(21)00158-9

**Published:** 2021-08

**Authors:** Daniel Freeman, Richard Emsley, Rowan Diamond, Nicola Collett, Emily Bold, Eleanor Chadwick, Louise Isham, Jessica C Bird, Danielle Edwards, David Kingdon, Ray Fitzpatrick, Thomas Kabir, Felicity Waite, Oxford Cognitive Approaches to Psychosis Trial Study Group, Lydia Carr, Chiara Causier, Emma Černis, Miriam Kirkham, Sinéad Lambe, Rachel Lister, Laina Rosebrock, Kathryn M Taylor, Ashley-Louise Teale, Eve Twivy

**Affiliations:** a Department of Psychiatry, University of Oxford, Oxford, UK; b Nuffield Department of Population Health, University of Oxford, Oxford, UK; c Oxford Health NHS Foundation Trust, Oxford, UK; d Department of Biostatistics and Health Informatics, King's College London, Institute of Psychiatry, Psychology and Neuroscience, London, UK; e Department of Clinical and Experimental Sciences, University of Southampton, Southampton, UK; f McPin Foundation, London, UK

## Abstract

**Background:**

There is a large clinical need for improved treatments for patients with persecutory delusions. We aimed to test whether a new theoretically driven cognitive therapy (the Feeling Safe Programme) would lead to large reductions in persecutory delusions, above non-specific effects of therapy. We also aimed to test treatment effect mechanisms.

**Methods:**

We did a parallel, single-blind, randomised controlled trial to test the Feeling Safe Programme against befriending with the same therapists for patients with persistent persecutory delusions in the context of non-affective psychosis diagnoses. Usual care continued throughout the duration of the trial. The trial took place in community mental health services in three UK National Health Service trusts. Participants were included if they were 16 years or older, had persecutory delusions (as defined by Freeman and Garety) for at least 3 months and held with at least 60% conviction, and had a primary diagnosis of non-affective psychosis from the referring clinical team. Patients were randomly assigned to either the Feeling Safe Programme or the befriending programme, using a permuted blocks algorithm with randomly varying block size, stratified by therapist. Trial assessors were masked to group allocation. If an allocation was unmasked then the unmasked assessor was replaced with a new masked assessor. Outcomes were assessed at 0 months, 6 months (primary endpoint), and 12 months. The primary outcome was persecutory delusion conviction, assessed within the Psychotic Symptoms Rating Scale (PSYRATS; rated 0–100%). Outcome analyses were done in the intention-to-treat population. Each intervention was provided individually over 6 months. This trial is registered with the ISRCTN registry, ISRCTN18705064.

**Findings:**

From Feb 8, 2016, to July 26, 2019, 130 patients with persecutory delusions (78 [60%] men; 52 [40%] women, mean age 42 years [SD 12·1, range 17–71]; 86% White, 9% Black, 2% Indian; 2·3% Pakistani; 2% other) were recruited. 64 patients were randomly allocated to the Feeling Safe Programme and 66 patients to befriending. Compared with befriending, the Feeling Safe Programme led to significant end of treatment reductions in delusional conviction (−10·69 [95% CI −19·75 to −1·63], p=0·021, Cohen's *d*=–0·86) and delusion severity (PSYRATS, −2·94 [–4·58 to −1·31], p<0·0001, Cohen's *d*=–1·20). More adverse events occurred in the befriending group (68 unrelated adverse events reported in 20 [30%] participants) compared with the Feeling Safe group (53 unrelated adverse events reported in 16 [25%] participants).

**Interpretation:**

The Feeling Safe Programme led to a significant reduction in persistent persecutory delusions compared with befriending. To our knowledge, these are the largest treatment effects seen for patients with persistent delusions. The principal limitation of our trial was the relatively small sample size when comparing two active treatments, meaning less precision in effect size estimates and lower power to detect moderate treatment differences in secondary outcomes. Further research could be done to determine whether greater effects could be possible by reducing the hypothesised delusion maintenance mechanisms further. The Feeling Safe Programme could become the recommended psychological treatment in clinical services for persecutory delusions.

**Funding:**

NIHR Research Professorship and NIHR Oxford Health Biomedical Research Centre.

## Introduction

When added to standard care, cognitive-behavioural therapy (CBT) for the positive symptoms of psychosis has been shown to reduce delusions and represents a substantial step forward in treatment options for patients with psychosis. Talking about delusions is no longer discouraged. However, the effect size of CBT for psychosis on delusions is small (approximately Cohen's *d*=0·3).^1^, ^2^, ^3^ This finding is in contrast to the large effect sizes seen for targeted CBT for social anxiety, for example (Cohen's *d*=1·56).^4^ There have been few tests of CBT for psychosis against alternative psychological treatments. In a programme of work, we set out to develop a new cognitive treatment, which was translated from an empirically established theoretical model, that would produce large effect size reduction in persecutory delusions for patients. We focused on persecutory delusions as factor analyses indicate that it is a distinct delusion type^5^ that is associated with very low psychological wellbeing^6^ and occurs in over 70% of patients who present with non-affective psychosis.^7^

**Research in context**

**Evidence before this study**

The main psychological treatment tested for delusions is cognitive behaviour therapy (CBT) for psychosis, which meta-analyses consistently show has clinical benefits on positive symptoms but of small size. CBT for psychosis has not shown better end of treatment clinical effects compared with the non-specific benefits of befriending. On Feb 26, 2021, we searched PubMed (with no date or language restrictions) using the following search terms: (Psychosis OR Psychotic OR Schizophrenia) AND (CBT OR Cognitive behavior OR Cognitive therapy OR Behavior therapy OR Behaviour therapy) AND (Randomised OR Randomized OR RCT) AND (Befriending). 167 papers were identified. The search process found only two trials that had compared CBT for psychosis to befriending. Neither trial showed end of treatment symptom differences. One trial showed that CBT had greater symptomatic reductions than befriending at later follow-up (9 months) and one trial did not (12 months).

**Added value of this study**

The approach taken to the building of the new therapy (Feeling Safe Programme) was to develop a multifactorial theoretical model of persecutory delusions, test in individual studies treatment techniques targeting each putative causal factor identified in the model, and then combine the successful techniques in a full therapy. Patients were consulted throughout therapy development, and patient personalisation and preference were included in the Feeling Safe Programme. This trial provides the first randomised controlled test of the new intervention, and estimates the benefits of the therapy above the non-specific effects of seeing a therapist. To our knowledge, the Feeling Safe Programme produced the largest treatment effects seen for patients with persistent delusions, and the size of clinical benefits are closer to those found for targeted cognitive behavioural therapy approaches for anxiety disorders.

**Implications of all the available evidence**

Large reductions in delusions, above non-specific therapy benefits, are achievable with targeted psychological treatment. To our knowledge, the Feeling Safe Programme has produced the largest treatment effects for persistent delusions reported to date. Evaluation of the Feeling Safe Programme in multiple different service sites is warranted, and we believe the treatment could be improved even further.

In our theoretical model, persecutory delusions were conceptualised as unfounded threat beliefs (developed in the context of genetic and environmental risk) that are maintained by several psychological processes, including excessive worry, low self-confidence, poor sleep, anomalous experiences, reasoning biases, and safety-seeking behaviours.^8^ The clinical implication of our model is that safety must be relearned by entering feared situations after the influence of the maintenance factors has been reduced. To build the new treatment, we did a series of studies evaluating individual modular interventions targeting each maintenance factor.^9^, ^10^, ^11^, ^12^ We also showed in a cohort of 1800 patients with psychosis that delusion maintenance factors identified in the model had a high prevalence rate and that patients would like them treated.^13^ The individual treatment modules were combined into the Feeling Safe Programme, in which patients chose modules identified via assessment as being relevant to them. A case series with 11 patients indicated the potential for a large treatment effect (Psychotic Symptoms Rating Scale [PSYRATS] *d*=2·3).^14^ Seven (64%) patients no longer held a delusion after treatment.

We tested the Feeling Safe Programme in a randomised controlled trial. It was anticipated that the Feeling Safe Programme would lead to 50% of patients recovering from their persistent persecutory delusion (defined as conviction falling below 50% [ie, greater doubt than certainty in the delusion]). To determine treatment effects above the non-specific benefits of seeing a therapist, the comparison condition was befriending with the same therapists. In two previous head-to-head comparisons, befriending and CBT for psychosis have not differed in end of treatment benefit for patients with psychosis.^15^, ^16^ It was hypothesised that the Feeling Safe Programme would lead to lower levels of conviction in the persecutory delusions than would befriending. The primary endpoint was prespecified as 6 months (ie, the end of treatment), but the persistence of effects at a longer follow-up (ie, 12 months) were also evaluated. Key secondary hypotheses were that the Feeling Safe Programme, compared with befriending, would lead to overall reductions in delusion severity, paranoia, depression, and suicidal ideation, and to improvements in psychological wellbeing, activity, and quality of life. We also aimed to test whether changes in the hypothesised delusion maintenance factors mediated change in delusions. Further, we also aimed to examine three potential moderators of treatment effects (working memory, illicit drug use, and anger) as to whether they would hinder successful therapy.

## Methods

### Study design

We did a randomised controlled explanatory trial with single-blind assessment in two parallel groups: the Feeling Safe Programme and befriending. This study was a single centre study (Oxford, UK), with patients recruited from community services in three local UK NHS trusts. Assessments were done at 0 months, 6 months (post treatment), and 12 months. The trial received approval from an NHS Research Ethics Committee (South Central–Oxford B Research Ethics Committee; ref 15/SC/0508), was registered prospectively, and the protocol published at the start of the trial (March 11, 2016).^17^

### Participants

Participants were included in this study if they: were 16 years or older, had persecutory delusions (as defined by Freeman and Garety)^18^ for at least 3 months and held with at least 60% conviction, and had a primary diagnosis of non-affective psychosis from the referring clinical team. Participants were excluded if they: were receiving another psychological therapy, had an insufficient comprehension of English, had a current primary diagnosis of substance or personality disorder, were being treated in forensic mental health services, had an organic syndrome, or had a learning disability. Written informed consent was obtained. Research assistants sought referrals from Oxford Health NHS Foundation Trust (Oxford, UK), Northamptonshire Healthcare NHS Foundation Trust (Northamptonshire, UK), and Berkshire Healthcare NHS Foundation Trust (Berkshire, UK).

### Randomisation and masking

Participants were randomly assigned to either the Feeling Safe Programme or befriending. Online randomisation (developed by the University of Oxford Primary Care Clinical Trials Unit and ran by FW) used a permuted blocks algorithm, with randomly varying block size, which was stratified by therapist.

The trial coordinator (FW) informed the therapist of allocations. Therapists provided both interventions to reduce the confounding of therapist effects and increase statistical power. Trial assessors were masked to group allocation. If an allocation was shown then re-masking occurred using another assessor. Assessors were unmasked on ten occasions and nine assessments were successfully re-masked (one 12-month assessment was done unmasked).

### Procedures

We aimed to provide each treatment individually in approximately 20 sessions over 6 months. Both treatments were provided by the same nine clinical psychologists, with weekly supervision from DF and FW. Sessions were audio recorded when permission from the participant was provided. Patient beliefs about the potential effectiveness of intervention were assessed after the first session with the Credibility/Expectancy Questionnaire.^19^

The Feeling Safe Programme is modular, personalised, and included patient preference.^14^ In the Feeling Safe Programme, after an assessment with DF or FW, the patient was offered a choice of treatment modules, which were then delivered by a clinical psychologist. The assessment principally comprised a clinical interview concerning a recent instance of persecutory thinking, identifying whether each model maintenance factor was relevant for the person (in the context of the trial formal baseline assessments). An overarching brief formulation, in terms of the maintenance factors, was developed with the patient. Based on the theoretical model,^8^ the range of modules offered were: improving sleep, reducing worry, increasing self-confidence, reducing the impact of voices, improving reasoning processes, and feeling safe enough. Typically, three to four modules were completed, based on patient preference, although all patients were encouraged to complete the feeling safe enough module (this module related to the dropping of safety-seeking behaviours in behavioural tests to reduce threat beliefs and build safety beliefs) before the end of treatment. Each module was written in a series of booklets shared by patient and therapist. A further concise formulation of the particular difficulty (eg, worry, insomnia, and safety-seeking behaviours) was included within each module. Modules varied in length but typically took approximately six sessions. Sessions were mostly weekly (but could be more frequent as needed). Participants could also have a different established evidence-based treatment module if there was a pressing clinical need (eg, dealing with panic attacks happening in sessions). The treatment in the Feeling Safe Programme differs from first generation CBT for psychosis by: inclusion of substantial elements that have not been included in the original manuals (eg, the focus on evaluating safety, addressing sleep dysfunction, worry, and positive self-beliefs); treatment proceeding via achieving (when possible) measured change in each targeted mechanism, one at a time, using a sustained approach; the highly manualised modular elements; implementation of active therapeutic techniques in the first sessions; and frequent contact with patients between sessions to help initiate change (eg, texts and telephone calls). To assess treatment quality, 12 tapes, chosen at random, were rated on the Cognitive Therapy Scale-Revised^20^ (CTS-R) by an independent clinical psychologist. All tapes were rated as providing at least satisfactory cognitive therapy (ie, a score of at least 3 on each CTS-R item). Befriending, called Feeling Safe and Supported, followed a protocol devised by one of the research team members (DK) that was previously successfully used in two large clinical trials for patients with psychosis.^15^, ^16^ The aim was to simulate how a good friend would respond, involving: a general focus on non-threatening topics (although patients were not actively dissuaded from talking about concerns), non-confrontation, empathy, and supportiveness. It was expected that this intervention would provide a break for patients from their fears and encourage re-engagement in activities. The rationale provided to patients was: “The goal of Feeling Safe and Supported is to help you feel safer, happier, and doing more of what you want in life. We know that regular time to connect with other people is good for everyone's wellbeing. You will have regular time being listened to, respected, and talking about everyday topics. This takes our minds off difficulties and helps us to feel better about ourselves. In Feeling Safe and Supported, you will have time to reflect on interests and activities that you enjoy, which can help to increase motivation to do these activities and spark new interests. This all helps us to feel secure, calm, and connected.” Befriending sessions were held typically weekly. 20 tapes of therapy sessions, selected at random from the two interventions, were all correctly categorised by an independent clinical psychologist into type of treatment.

### Outcomes

The primary outcome measure was self-reported conviction in the persecutory delusion, assessed using the PSYRATS (rated 0–100%).^21^ Secondary outcomes were measures of overall paranoia (measured using the Revised Green et al Paranoid Thoughts Scale),^22^ suicidal ideation (measured using the Columbia-Suicide Severity Rating Scale),^23^ depression (measured using the Beck Depression Inventory),^24^ anger (measured using the Dimensions of Anger Reactions [DAR-5] scale),^25^ verbal auditory hallucinations (measured using the auditory hallucinations items from the Specific Psychotic Experiences Questionnaire [SPEQ]),^26^ anhedonia (measured using the anticipatory scale of the Temporal Experience of Pleasure Scale),^27^ psychological wellbeing (measured using the Warwick-Edinburgh Mental Wellbeing Scale),^28^ quality of life (measured using the EQ-5D-5L^29^ and the Long-Term Conditions Questionnaire [LTCQ]),^30^ patient satisfaction (measured using the adapted choice of outcome in CBT for psychoses questionnaire),^31^ and activity (measured using the daily step count and time-budget measure).^32^

For mediation, we included measures of: beliefs about safety (“I generally feel safe around other people”) and vulnerability (“I feel vulnerable”) on 0–100 visual analogue scales; worry (measured using the Penn State Worry Questionnaire; PSWQ);^33^ beliefs about self and others (measured using the Brief Core Schema Scales);^34^ anomalous experiences (measured using the hallucinations item of the SPEQ);^26^ insomnia (measured using the Insomnia Severity Index);^35^ jumping to conclusions (measured using the 60:40 beads task);^36^ possibility of being mistaken (rated 0–100%);^37^ and safety-seeking behaviours (measured using the Safety Behaviours Questionnaire–Persecutory Delusions).^38^ Included as moderators were: working memory (WAIS-III digit span forward, digit span backward, and letter-number sequencing);^34^ illicit drug use (measured using the Maudsley Addiction Profile);^39^ and anger (measured using the Dimensions of Anger Reactions scale).^25^ Service use was recorded using an adapted version of the Economic Patient Questionnaire,^40^ which included questions from the Client Service Receipt Inventory.^41^

At the end of trial participation, we checked medical notes for the following adverse events: death, suicide attempts, serious violent incidents, admissions to forensic units, formal complaints about therapy, and hospital admission. We also recorded any such event that we became aware of during a patient's participation. An independent data monitoring and ethics committee (DMEC) chair rated whether any adverse event was related to treatment or trial procedures.

### Changes to the protocol

The pre-trial protocol (Dec 9, 2015) is provided in the appendix (pp 1–26). The following changes were made to the protocol after the start of the trial. For the statistical analysis plan, approved by the Trial Steering Committee and DMEC before any inspection of outcome data, the EQ-5D-5L was added as a secondary outcome and four model-relevant variables were added as potential mediators (ie, safety belief, vulnerability belief, belief flexibility, and the hallucinations scale from the SPEQ). The DAR-5 was added as a potential moderator of outcome. It was also agreed to use the revised scoring method of the Green et al Paranoid Thoughts Scale.^22^ Further details can be found in the appendix (p 71).

### Statistical analysis

The statistical analysis plan (appendix pp 27–42) was approved by the independent chair of the Trial Steering Group and the members of the DMEC before any inspection of post-randomisation data. A full statistical report is provided in the appendix (pp 43–70).

For the primary continuous outcome and secondary outcomes, linear mixed-effect models were used, with outcome measurement (at the two follow-up timepoints) as the dependant variable. The models included fixed effects for timepoint, treatment, timepoint by treatment interactions, the baseline measure of the outcome, and therapist, assuming a linear relationship between baseline and outcome. The dichotomous outcome of recovery in the delusion was analysed using a logistic mixed-effect model. Persecutory delusion conviction was analysed as a continuous and also as a dichotomous (recovery) variable. The models included a random intercept for participant, an unstructured correlation matrix for the residuals, and were fitted using restricted maximum likelihood estimation. The primary analysis assumed data were missing at random, conditional on the observed values of the outcome at baseline and follow-up, and on other covariates in the model. For each outcome and timepoint, we report the treatment effect estimate as the adjusted mean difference between groups, its SE, 95% CIs, and p value. In addition, we report estimates for Cohen's *d* effect sizes as the adjusted mean difference of the outcome (between the groups) divided by the sample SD of the outcome at baseline.

Parametric regression models tested for mediation of the Feeling Safe Programme on outcome through the putative mediators. Analyses were adjusted for baseline measures of the mediator, outcomes, and possible measured confounders. Moderators were assessed separately by repeating the primary analysis models and including interaction terms between the randomised intervention and each moderator.

For a recovery rate in delusions of 50% in the Feeling Safe Programme, compared with 20% with befriending, a trial would have over 90% power with 60 patients in each group. The trial would, however, gain greater power by examining change in delusion dimensional scores. If the standardised effect of the new intervention compared with befriending was smaller than ten percentage points on the conviction scale (0–100%; *d*=0·5), then we would not consider further development of the intervention to be worth pursuing. If the true effect size was a ten point difference (SD=20), then a two-sample t-test with a two-sided significance level of 0·05 would have 80% power to detect a significant effect with outcome data available for 64 participants per randomised group. We aimed to recruit 75 participants per group, conservatively allowing for a dropout rate of 15%. All analyses were done in Stata version 16.0 (StataCorp. 2019. *Stata Statistical Software*: Release 16. College Station, TX: StataCorp LLC).^42^ This trial is registered with the ISRCTN registry, ISRCTN18705064.

### Role of the funding source

The funder reviewed the study as part of the professorship application. Research assistant costs were also supported by the NIHR Oxford Health Biomedical Research Centre (Oxford, UK). The funders of the study had no role in study design, data collection, data analysis, data interpretation, or writing of the report. The corresponding author (DF), trial coordinator (FW), and statisticians had full access to all the data in the study and the corresponding author had final responsibility for the decision to submit for publication.

## Results

Recruitment took place from Feb 8, 2016, to July 26, 2019, with final follow-up data collected on July 6, 2020. Of 321 patients assessed, 64 patients were randomly allocated to the Feeling Safe Programme and 66 patients to the befriending group (figure). The average age of participants was approximately 40 years, there was a higher proportion of men than women, most patients were unemployed, the most frequent diagnosis was schizophrenia, and almost all participants were prescribed antipsychotic medication (table 1). The two groups were reasonably balanced across baseline characteristics.

**Figure:**
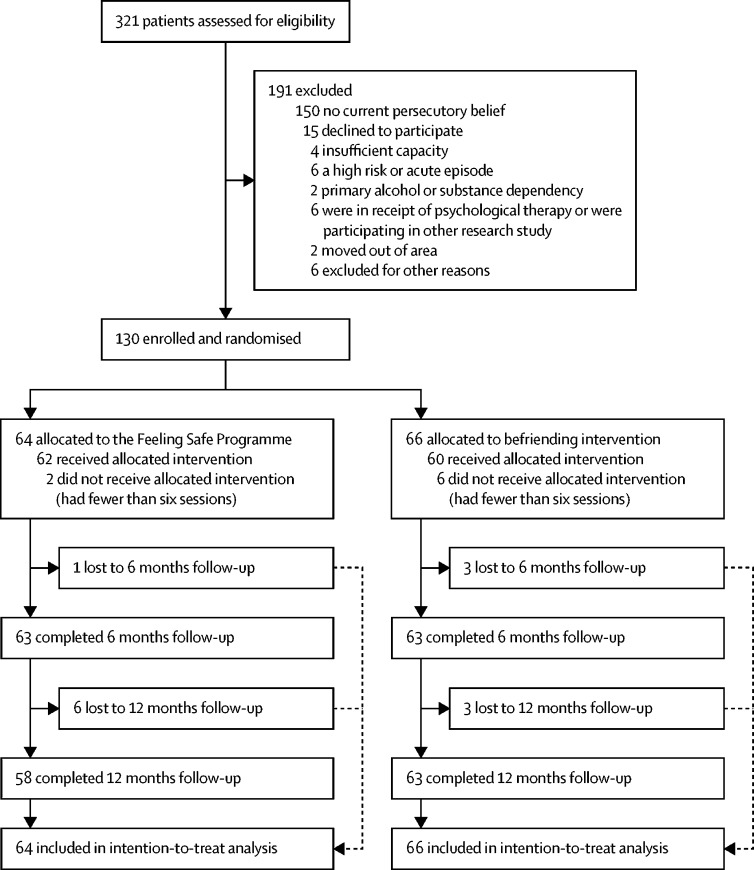
Trial profile

**Table 1:** Sociodemographic and clinical characteristics of the participant group

		**Befriending (n=66)**	**The Feeling Safe Programme (n=64)**	**All participants (n=130)**
Age, years		41·3 (12·0)	41·9 (12·3)	41·6 (12·1)
Gender				
	Women	22 (33%)	30 (47%)	52 (40%)
	Men	44 (67%)	34 (53%)	78 (60%)
Ethnicity				
	White	58 (88%)	52 (81%)	110 (85%)
	Black Caribbean	5 (8%)	3 (5%)	8 (6%)
	Black African	0	2 (3%)	2 (2%)
	Black other	0	1 (2%)	1 (1%)
	Indian	1 (2%)	2 (3%)	3 (2%)
	Pakistani	1 (2%)	2 (3%)	3 (2%)
	Chinese	0	1 (2%)	1 (1%)
	Other	1 (2%)	1 (2%)	2 (2%)
Employment				
	Unemployed	51 (77%)	51 (80%)	102 (79%)
	Employed (full-time)	5 (8%)	1 (2%)	6 (5%)
	Employed (part-time)	5 (8%)	4 (6%)	9 (7%)
	Self employed	0	1 (2%)	1 (1%)
	Retired	2 (3%)	2 (3%)	4 (3%)
	Student	2 (3%)	2 (3%)	4 (3%)
	Housewife or househusband	1 (2%)	3 (5%)	4 (3%)
Marital status				
	Single	49 (75%)	43 (67%)	92 (71%)
	Cohabiting	2 (3%)	0	2 (2%)
	Married or in a civil partnership	8 (12%)	18 (28%)	26 (20%)
	Divorced	7 (11%)	3 (5%)	10 (8%)
Living situation				
	Living alone (with or without children)	29 (44%)	23 (36%)	52 (40%)
	Living with spouse	10 (15%)	14 (22%)	24 (19%)
	Living together as a couple	2 (3%)	2 (3%)	4 (3%)
	Living with parents	17 (26%)	10 (16%)	27 (21%)
	Living with other relatives	0	4 (6%)	4 (3%)
	Living with others (not listed above)	8 (12%)	11 (17%)	19 (15%)
Clinical diagnosis				
	Schizophrenia	43 (65%)	36 (56%)	79 (61%)
	Schizoaffective disorder	9 (14%)	13 (20%)	22 (17%)
	Delusional disorder	2 (3%)	2 (3%)	4 (3%)
	Psychosis NOS	12 (18%)	13 (20%)	25 (19%)
Medication				
	Antipsychotic	64 (97%)	61 (95%)	125 (96%)
	Antidepressant	47 (71%)	32 (50%)	79 (61%)
	Anxiolytic	4 (6%)	7 (11%)	11 (9%)
	Mood stabiliser	10 (15%)	8 (13%)	18 (14%)
	Hypnotic	7 (11%)	5 (8%)	12 (9%)
	Total number of psychotropics	2·6 (1·2)	2·3 (1·3)	2·4 (1·2)
	Antipsychotic CPZ equivalent dose (mg per day)	514·1 (412·9)	449·9 (392·7)	482·7 (402·8)

Data are mean (SD) or n (%). CPZ=chlorpromazine. NOS=not otherwise specified.

Patients allocated to the Feeling Safe Programme attended an average of 19·1 (SD=6·7; median=19) therapy sessions, totalling 1195·2 (464·1) min, and patients allocated to befriending attended an average of 16·4 (5·7; median 18) sessions, totalling 906·4 (352·6) min. Defining a dose of therapy as at least six sessions, 62 (97%) patients had a dose of the Feeling Safe Programme, and 62 (94%) had a dose of befriending. In the Feeling Safe Programme, an average of 2·70 (SD=1·00; median 3) modules were completed. The modules completed were feeling safe enough (56 [88%] patients), self-confidence (32 [50%] patients), worry (28 [44%] patients), voices (21 [33%] patients), sleep (20 [31%] patients), and reasoning (1 [2%] patient). 15 (23%) patients also had an additional module type. The first treatment module completed was: worry (23 [37%] patients), sleep (14 [23%] patients), self-confidence (13 [21%] patients), feeling safe enough (seven [11%] patients), or voices (five [8%] patients).

The second treatment module completed was: feeling safe enough (19 [33%] patients), self-confidence (12 [21%] patients), voices (10 [18%] patients), other (8 [14%] patients), sleep (5 [9%] patients), worry (2 [4%] patients), or reasoning (1 [2%] patient). The third treatment module completed was: feeling safe enough (22 [52%] patients), voices (8 [19%] patients), self-confidence (5 [12%] patients), other (4 [10%] patients), worry (2 [5%] patients), or sleep (1 [2%] patient). Treatment credibility (t=–1·387, p=0·168) and expectancy (t=–1·748, p=0·083) did not significantly differ between the two therapy groups (Feeling Safe Programme credibility mean score=21·2 [SD=4·42], expectancy mean score=19·58 [SD=5·04]; befriending credibility mean score=21·0 [SD=5·03], and expectancy mean score=17·96 [SD=4·99]).

Table 2 summarises primary and secondary outcomes data. The baseline PSYRATS scores indicate a high severity of persecutory delusions. At baseline, 41 (32%) patients rated their degree of conviction in the delusional belief as 100%, and 122 (93·8%) patients rated their delusion conviction as 70% or higher. At the primary end of treatment timepoint, the Feeling Safe Programme, in comparison to befriending, led to significant reductions in delusional conviction (mean difference=–10·69 [95% CI −19·75 to −1·63], p=0·021, Cohen's *d*=–0·86), delusion severity (−2·94 [–4·58 to −1·31], p<0·0001, Cohen's *d*=–1·20), overall paranoia (−5·59 [–10·5 to −0·83], p=0·032), anger (−1·45 [–2·63 to −0·27], p=0·016), and ideas of reference (−2·39 [–4·42 to −0·36], p=0·021), and led to significant improvements in psychological wellbeing (5·09 [2·28 to 7·89], p<0·0001), patient satisfaction (7·92 [1·90 to 13·93], p=0·010), activity assessed by the time budget assessment (5·03 [0·92 to 9·15], p=0·016), and quality of life (measured via EQ-5D-5L; 0·07 [0·01 to 0·14], p=0·027). At end of treatment there were no significant group differences in ideas of persecution (−2·86 [–6·02 to 0·31], p=0·077), quality of life (measured via LTCQ; 3·31 [–0·11 to 6·73], p=0·058), depression (−2·32 [–5·52 to 0·88], p=0·155), hallucinations (−1·07 [–2·27 to 0·13], p=0·080), suicidal ideation (0·18 [–0·25 to 0·62], p=0·408), anhedonia (1·14 [–1·21 to 3·50], p=0·340), activity assessed by step-count (482·98 [–769·21 to 1735·17], p=0·450), or EQ-5D quality of life health today rating (1·59 [–5·12 to 8·30], p=0·643).

**Table 2:** Effect of treatment on primary and secondary outcomes

	**Befriending (n=66)**	**The Feeling Safe Programme (n=64)**	Adjusted difference*(95% CI, SE); p value	**Cohen d (95% CI)**
**Primary analysis: conviction (dimensional)**				
Baseline	86·4 (12·6), n=66	87·1 (12·2), n=64	..	..
6 months	59·6 (27·1), n=63	49·4 (35·5), n=63	−10·69 (4·62); p=0·021 (−19·75 to −1·63)	−0·86 (−1·60 to −0·13)
**Primary analysis: conviction (dichotomous)**				
Baseline	0 (0%), n=66	0 (0%), 64	..	..
6 months	22 (34·9%), n=63	32 (50·8%), n=63	OR=3·94 (2·96); p=0·067 (0·91 to 17·13)	..
**Secondary analysis: PSYRATS (delusion severity)**†				
Baseline	18·2 (2·6), n=66	18·5 (2·3), n=64	..	..
6 months	14·2 (4·8), n=62	11·6 (5·9), n=61	−2·94 (0·83); p<0·0001 (−4·58 to −1·31)	−1·20 (−1·86 to −0·53)
12 months	13·5 (5·6), n=63	11·6 (6·4), n=55	−2·14 (0·85); p=0·012 (−3·80 to −0·47)	−0·87 (−1·54 to −0·19)
**Secondary analysis: 50% PSYRATS (delusion severity) reduction**†				
Baseline	..	..	..	..
6 months	9 (13·6%), n=62	21 (32·8%), n=61	OR=7·76 (6·77); p=0·019 (1·40 to 42·99)	..
12 months	10 (15·2%), n=63	21 (32·8%), n=55	OR=6·26 (5·31); p=0·031 (1·19 to 33·02)	..
**Secondary analysis: psychological wellbeing (WEMWBS)**†				
Baseline	35·1 (8·8), n=66	34·0 (8·2), n=64	..	..
6 months	39·7 (10·8), n=63	43·8 (10·1), n=61	5·09 (1·43); p<0·0001 (2·28 to 7·89)	0·60 (0·27 to 0·93)
12 months	39·4 (9·6), n=62	41·3 (10·0), n=57	2·26 (1·46); p=0·121 (−0·60 to 5·12)	0·27 (−0·07 to 0·60)
**Secondary analysis: patient satisfaction (CHOICE)**†				
Baseline	48·5 (18·7), n=65	47·7 (15·0), n=62	..	..
6 months	61·7 (21·1), n=60	68·3 (21·4), n=55	7·92 (3·07); p=0·010 (1·90 to 13·93)	0·47 (0·11 to 0·82)
12 months	61·4 (23·3), n=59	69·3 (21·3), n=53	7·56 (3·11); p=0·015 (1·46 to 13·66)	0·45 (0·09 to 0·81)
**Secondary analysis: Revised Green et al Paranoid Thoughts Scale (total)**†				
Baseline	44·8 (14·6), n=65	43·9 (13·8), n=63	..	..
6 months	29·8 (17·3), n=62	24·4 (18·4), n=60	−5·59 (2·43) p=0·021 (−10·35 to −0·83)	−0·39 (−0·73 to −0·06)
12 months	31·0 (19·7), n=61	24·4 (17·6), n=57	−5·92 (2·46) p=0·016 (−10·75 to −1·10)	−0·42 (−0·76 to −0·08)
**Secondary analysis: activity (time budget)**†				
Baseline	56·3 (14·3), n=64	51·0 (15·0), n=62	..	..
6 months	59·8 (15·6), n=59	59·4 (15·3), n=55	5·03 (2·10); p=0·016 (0·92 to 9·15)	0·34 (0·06 to 0·62)
12 months	61·1 (16·9), n=55	57·7 (15·6), n=49	1·99 (2·18); p=0·362 (−2·29 to 6·26)	0·13 (−0·15 to 0·42)
**Secondary analysis: anger**†				
Baseline	10·7 (4·5), n=65	11·1 (4·8), n=62	..	..
6 months	9·6 (4·3), n=60	8·3 (3·4), n=52	−1·45 (0·60); p=0·016 (−2·63 to −0·27)	−0·31 (−0·57 to −0·06)
12 months	9·6 (5·1), n=57	9·1 (4·5), n=51	−0·47 (0·61); p=0·445 (−1·66 to 0·73)	−0·10 (−0·36 to 0·16)
**Secondary analysis: Revised Green et al Paranoid Thoughts Scale: reference (part A)**†				
Baseline	17·4 (8·2), n=65	17·3 (7·2), n=63	..	..
6 months	12·6 (8·2), n=62	10·2 (7·1), n=60	−2·39 (1·04); p=0·021 (−4·42 to −0·36)	−0·31 (−0·58 to −0·05)
12 months	13·1 (9·0), n=61	10·2 (7·9), n=57	−2·53 (1·05); p=0·016 (−4·59 to −0·46)	−0·33 (−0·60 to −0·06)
**Secondary analysis: EQ5D—index**†				
Baseline	0·5 (0·3), n=65	0·5 (0·3), n=64	..	..
6 months	0·6 (0·3), n=64	0·6 (0·2), n=56	0·07 (0·03); p=0·027 (0·01 to 0·14)	0·28 (0·03 to 0·53)
12 months	0·6 (0·3), n=61	0·6 (0·2), n=54	0·05 (0·03); p=0·169 (−0·02 to 0·11)	0·18 (−0·08 to 0·43)
**Secondary analysis: Revised Green et al Paranoid Thoughts Scale: persecution (part B)**‡				
Baseline	27·4 (8·5), n=66	26·7 (8·2), n=63	..	..
6 months	17·2 (11·1), n=62	14·3 (11·8), n=60	−2·86 (1·61); p=0·077 (−6·02 to 0·31)	−0·34 (−0·72 to 0·04)
12 months	17·9 (12·2), n=61	14·2 (12·3), n=57	−3·34 (1·64); p=0·042 (−6·56 to −0·12)	−0·40 (−0·78 to −0·01)
**Secondary analysis: long-term conditions questionnaire**‡				
Baseline	35·2 (11·9), n=64	34·5 (8·7), n=61	..	..
6 months	41·6 (13·7), n=59	44·9 (13·0), n=55	3·31 (1·74); 0·058 (−0·11 to 6·73)	0·32 (−0·01 to 0·64)
12 months	41·6 (14·2), n=59	44·3 (11·5), n=52	2·11 (1·78); p=0·234 (−1·37 to 5·60)	0·20 (−0·13 to 0·54)
**Secondary analysis: The Beck Depression Inventory (depression)**‡				
Baseline	31·9 (12·4), n=66	30·2 (11·3), n=64	..	..
6 months	21·5 (12·6), n=61	18·8 (12·0), n=59	−2·32 (1·63); p=0·155 (−5·52 to 0·88)	−0·20 (−0·46 to 0·07)
12 months	23·1 (13·8), n=60	20·3 (13·5), n=56	−1·67 (1·66); p=0·315 (−4·92 to 1·59)	−0·14 (−0·41 to 0·13)
**Secondary analysis: hallucinations**‡				
Baseline	7·4 (5·9), n=65	7·6 (5·9), n=64	..	..
6 months	6·4 (5·5), n=61	6·1 (5·8), n=59	−1·07 (0·61); p=0·080 (−2·27 to 0·13)	−0·18 (−0·39 to 0·02)
12 months	5·1 (5·2), n=62	7·0 (5·7), n=58	1·10 (0·61); p=0·073 (−0·10 to 2·29)	0·19 (−0·02 to 0·39)
**Secondary analysis: suicidal ideation (CSSRS)**‡				
Baseline	2·0 (1·6), n=65	1·9 (1·6), n=62	..	..
6 months	1·4 (1·6), n=58	1·4 (1·6), n=54	0·18 (0·22); p=0·408 (−0·25 to 0·62)	0·12 (−0·16 to 0·39)
12 months	1·3 (1·4), n=55	1·5 (1·7), n=48	0·33 (0·23); p=0·153 (−0·12 to 0·79)	0·21 (−0·08 to 0·50)
**Secondary analysis: anhedonia**‡				
Baseline	30·1 (11·8), n=65	30·8 (11·0), n=64	..	..
6 months	34·1 (12·4), n=61	35·6 (12·0), n=58	1·14 (1·20); p=0·340 (−1·21 to 3·50)	0·10 (−0·11 to 0·31)
12 months	32·5 (12·0), n=60	35·1 (10·3), n=57	1·44 (1·21); p=0·235 (−0·94 to 3·82)	0·13 (−0·08 to 0·34)
**Secondary analysis: step count**‡				
Baseline	7910·9 (6043·2), n=47	6176·1 (3425·2), n=34	..	..
6 months	6749·1 (4826·7), n=38	6413·1 (4307·8), n=28	482·98 (638·89); p=0·450 (−769·21 to 1735·17)	0·09 (−0·15 to 0·34)
12 months	..	..	..	..
**Secondary analysis: EQ5D—health today**‡				
Baseline	47·9 (21·0), n=65	50·2 (20·2), n=64	..	..
6 months	56·5 (23·3), n=62	58·4 (20·7), n=58	1·59 (3·42); p=0·643 (−5·12 to 8·30)	0·08 (−0·25 to 0·40)
12 months	53·8 (25·2), n=61	60·7 (21·1), n=54	6·80 (3·51); p=0·052 (−0·07 to 13·68)	0·33 (−0·01 to 0·67)
**Primary outcomes at follow-up: conviction (dimensional)**				
Baseline	86·4 (12·6), n=66	87·1 (12·2), n=63	..	..
12 months	59·4 (32·8), n=63	50·2 (36·0), n=58	−8·43 (4·71); p=0·074 (−17·67 to 0·81)	−0·68 (−1·43 to 0·07)
**Primary outcomes at follow-up: conviction (dichotomous)**				
Baseline	0 (0%), n=66	0 (0%)		
12 months	22 (34·9%), n=63	27 (46·6%), n=58	OR=2·36 (1·74); p=0·242 (0·56 to 10·00)	

Data are unadjusted mean (SD) or n (%), unless otherwise specified. CHOICE=Choice of outcome in CBT for psychoses. OR=odds ratio. PSYRATS=Psychotic Symptoms Rating Scale. WEMWBS=The Warwick-Edinburgh Mental Wellbeing Scale.

* Adjusted for baseline score, therapist, interaction of timepoint with treatment allocation, and for including a random effect at the individual level.

† End of treatment statistically significant findings.

‡ End of treatment non-statistically significant findings.

The Feeling Safe Programme led to a recovery rate of 50·8%, although this rate did not reach significance in comparison to the befriending group (34·9%; odds ratio=3·94 [95% CI 0·91 to 17·13], p=0·067). Overall, it can be seen that there were large effects of the Feeling Safe Programme on persecutory delusions, but the size of significant changes in other measures that occurred were typically more modest. The gains made by the Feeling Safe participants persisted at 12 months (table 2), although because of a slightly lower number of patients followed-up at this later timepoint and slight fluctuations in scores, not all the previous differences remained significant. Sensitivity analyses are reported in the appendix (pp 60–64).

The effect of treatment on the mediator variables is summarised in table 3. Compared with befriending, there were significant end of treatment improvements with the Feeling Safe Programme in the possibility of being mistaken (mean difference=13·92 [95% CI 5·17 to 22·68], p=0·002), vulnerability beliefs (−12·67 [–21·94 to −3·40], p=0·007), positive beliefs about others (2·24 [0·84 to 3·64], p=0·002), insomnia (−2·98 [–4·88 to −1·09], p=0·002), worry (−3·33 [–6·59 to −0·06], p=0·046), and negative self-beliefs (−1·63 [–3·16 to −0·09], p=0·038). The effect sizes were small to moderate. There were no significant group differences in positive self-beliefs (1·26 [–0·19 to 2·70], p=0·088), negative self-beliefs (−1·30 [–2·84 to 0·24], p=0·097), safety beliefs (6·52 [–2·23 to 15·27], p=0·075), safety behaviours (−2·22 [–6·93 to 3·61], p=0·574), and anomalous experiences (−0·76 [–3·61 to 2·10], p=0·604). At end of treatment, befriending improved jumping to conclusions more than the Feeling Safe Programme (−1·12 [–2·20 to −0·05], p=0·041). Change in a mediator was most often greater in size for those patients who had the associated module (appendix pp 65–66). Mediation analyses are shown in the appendix (pp 53–58). The proportions of Feeling Safe treatment change in delusional conviction at 6 months mediated by each factor (assessed in separate analyses) were: vulnerability belief (97·7%), possibility of being mistaken (94·1%), positive other beliefs (41·1%), safety beliefs (38·7%), negative self-beliefs (33·8%), worry (27·1%), negative other beliefs (25·5%), positive self-beliefs (16·4%), insomnia (14·8%), safety-seeking behaviours (11·2%), anomalous experiences (7·3%), and jumping to conclusions (0·6%). There was no evidence of treatment moderation at end of treatment by working memory, illicit drug use, or anger (appendix pp 59–60).

**Table 3:** Effect of treatment on putative mediators

	**Befriending (n=66)**	**The Feeling Safe Programme (n=64)**	Adjusted difference*(95% CI, SE); p value	**Cohen d (95% CI)**
**Possibility of being mistaken**†				
Baseline	19·92 (20·90), n=66	19·20 (21·21), n=64	..	..
6 months	36·80 (28·07), n=61	48·11 (34·65), n=61	13·92 (4·47); p=0·002 (5·17 to 22·68)	0·66 (0·25 to 1·08)
12 months	35·05 (30·76), n=62	47·16 (33·98), n=57	12·53 (4·52); p=0·006 (3·67 to 21·40)	0·60 (0·17 to 1·02)
**Vulnerability**†				
Baseline	75·15 (23·26), n=66	72·28 (24·07), n=64	..	..
6 months	63·7 (26·89), n=62	49·31 (32·24), n=61	−12·67 (4·73); p=0·007 (−21·94 to −3·40)	−0·54 (−0·93 to −0·14)
12 months	56·73 (2733), n=62	47·77 (31·07), n=57	−7·20 (4·81); p=0·134 (−16·63 to 2·23)	−0·30 (−0·70 to 0·09)
**BCSS positive others**†				
Baseline	9·60 (4·14), n=65	9·11 (4·90), n=62	..	..
6 months	10·05 (4·17), n=61	12·20 (5·96), n=54	2·24 (0·71); p=0·002 (0·84 to 3·64)	0·50 (0·19 to 0·81)
12 months	9·98 (4·41), n=59	11·15 (5·40), n=52	0·98 (0·73); p=0·176 (−0·44 to 2·40)	0·22 (−0·10 to 0·53)
**Insomnia**†				
Baseline	14·02 (6·36), n=65	13·52 (7·4), n=61	..	..
6 months	12·25 (6·81), n=60	8·5 (6·21), n=52	−2·98 (0·97); p=0·002 (−4·88 to −1·09)	−0·43 (−0·71 to −0·16)
12 months	13·32 (6·60), n=57	10·17 (6·78), n=52	−2·02 (0·97); p=0·037 (−3·92 to −0·13)	−0·29 (−0·57 to −0·02)
**PSWQ total (worry)**†				
Baseline	63·26 (11·27), n=65	62·58 (10·68), n=62	..	..
6 months	57·35 (12·78), n=60	54·25 (15·51), n=52	−3·33 (1·67); p=0·046 (−6·59 to −0·06)	−0·30 (−0·60 to −0·01)
12 months	58·30 (12·21), n=57	54·65 (11·34), n=52	−3·11 (1·68); p=0·064 (−6·40 to 0·18)	−0·28 (−0·58 to 0·02)
**BCSS negative self**†				
Baseline	11·98 (5·77), n=65	11·81 (5·31), n=62	..	..
6 months	9·11 (5·79), n=61	7·78 (5·84), n=54	−1·63 (0·78); p=0·038 (−3·16 to −0·09)	−0·29 (−0·57 to −0·02)
12 months	9·53 (6·27), n=58	8·46 (5·84), n=52	−1·15 (0·80); p=0·151 (−2·72 to 0·42)	−0·21 (−0·49 to 0·08)
**Jumping to conclusions**†				
Baseline	4·14 (4·47), n=63	3·50 (3·2), n=56	..	..
6 months	4·98 (4·62), n=55	3·57 (3·34), n=51	−1·12 (0·55); p=0·041 (−2·20 to −0·05)	−0·28 (−0·56 to −0·01)
12 months	4·49 (3·91), n=51	4·08 (3·26), n=39	−0·19 (0·58); p=0·750 (−1·33 to 0·96)	−0·05 (−0·34 to 0·24)
**BCSS positive self**‡				
Baseline	7·35 (4·65), n=66	7·84 (5·17), n=62	..	..
6 months	8·43 (5·15), n=61	9·93 (5·53), n=54	1·26 (0·74); p=0·088 (−0·19 to 2·70)	0·26 (−0·04 to 0·55)
12 months	9·09 (4·73), n=58	9·19 (6·23), n=52	−0·48 (0·76); p=0·527 (−1·96 to 1·00)	−0·10 (−0·40 to 0·20)
**BCSS negative others**‡				
Baseline	13·89 (5·33), n=64	14·34 (5·15), n=62	..	..
6 months	9·74 (6·39), n=61	8·87 (6·40), n=54	−1·30 (0·79); p=0·097 (−2·84 to 0·24)	−0·25 (−0·54 to 0·04)
12 months	11·32 (6·17), n=59	9·08 (6·56), n=52	−2·34 (0·80); p=0·003 (−3·90 to −0·78)	−0·45 (−0·75 to −0·15)
**Safety beliefs**‡				
Baseline	37·88 (29·64), n=65	37·75 (26·34), n=64	..	..
6 months	50·67 (28·71), n=63	55·62 (29·41), n=61	6·52 (4·46); p=0·144 (−2·23 to 15·27)	0·23 (−0·08 to 0·55)
12 months	49·03 (29·47), n=62	57·00 (28·58), n=57	8·09 (4·54); p=0·075 (−0·80 to 16·99)	0·29 (−0·03 to 0·61)
**Safety behaviours**‡				
Baseline	34·14 (16·58), n=65	33·85 (16·91), n=62	..	..
6 months	21·98 (16·54), n=58	19·85 (18·49), n=52	−2·22 (2·40); p=0·355 (−6·93 to 2·48)	−0·13 (−0·42 to 0·15)
12 months	20·78 (15·46), n=54	18·73 (13·35), n=45	−1·41 (2·51); p=0·574 (−6·34 to 3·51)	−0·08 (−0·38 to 0·21)
**Anomalous experiences**‡				
Baseline	21·80 (15·02), n=65	22·38 (14·63), n=64	..	..
6 months	15·57 (12·87), n=61	16·34 (14·93), n=59	−0·76 (1·46); p=0·604 (−3·61 to 2·10)	−0·05 (−0·24 to 0·14)
12 months	14·37 (12·97), n=60	19·16 (15·61), n=57	2·02 (1·48); p=0·171 (−0·87 to 4·92)	0·14 (−0·06 to 0·33)

Data are unadjusted mean (SD) or n (%), unless otherwise specified. BCSS=Brief Core Schema Scales. PSWQ=Penn State Worry Questionnaire.

* Adjusted for baseline score, therapist, interaction of timepoint with treatment allocation, and for including a random effect at the individual level.

† End of treatment statistically significant findings.

‡ End of treatment non-statistically significant findings.

The provision of usual care in each group is summarised in the appendix (pp 44, 65–68). Eight individuals allocated to befriending received other psychological therapy during the trial compared with three individuals allocated to the Feeling Safe Programme.

Over 12 months, 16 people (ten men; six women) allocated to the Feeling Safe Programme had a total of 53 adverse events and 20 people (13 men; seven women) in the befriending group had a total of 68 adverse events (appendix pp 45–46). No adverse events were classified as related to trial treatment or procedures.

## Discussion

Both the Feeling Safe Programme and befriending were taken up by patients to a very high degree and were associated with clinical improvement. The targeted cognitive therapy led to large clinical changes in persecutory delusions that were greater than the non-specific relationship benefits of befriending. We believe these results show that if a proven theoretical model is translated into focused intervention techniques that are implemented intensively—within an intervention framework that explicitly addresses the multifactorial complexity of causation in psychosis and patient preference—then major improvements in treatment outcomes are possible. The effect size change in overall delusion severity (*d*=1·20), as assessed by the PSYRATS assessment that is routinely used in psychosis clinical trials, is closer to those seen in the successful targeted cognitive approaches for anxiety disorders. The treatment approach is modular and manualised, which will facilitate implementation. At the end of treatment there were significant improvements with the Feeling Safe Programme in several other important outcomes, but these were of a more modest effect size above befriending. At the end of treatment there were significant improvements with the Feeling Safe Programme compared with befriending in seven other secondary outcomes (ie, overall paranoia, anger, ideas of reference, psychological wellbeing, patient satisfaction, time use, and EQ-5D index of quality of life). Befriending showed no significant improvements above the Feeling Safe Programme in any outcome. There were no significant group differences in eight secondary outcomes (ie, ideas of persecution, LTCQ quality of life, depression, hallucinations, suicidal ideation, anhedonia, step-count, and EQ-5D quality of life health rating). The Feeling Safe Programme treatment gains were largely maintained over time. In sum, the specific focus of the new cognitive intervention led to large clinical effects on persecutory delusions that were additional to those of befriending, but there were much more modest differences, when they appeared, between the two approaches for the general secondary outcomes. The benefit arising from the therapeutic relationship common to psychological therapies is clearly very important, and befriending is a valuable approach for patients with psychosis, but the addition of specific techniques can lead to larger change, principally in the specific targeted outcome. Type of therapy received is not inconsequential.

The analysis of the mediators is also informative. The Feeling Safe Programme improved many of the hypothesised mediators and these explained a large proportion of the treatment effect on persecutory delusions. However, the effect size on the mediators was generally lower than hoped, although it should be kept in mind that only a proportion of patients completed each module type. There was also a greater effect on vulnerability beliefs than the development of safety beliefs, which indicates a weakening of the influence of threat-based memories, but insufficient establishment of alternative safety beliefs. The theoretical model has been translated into efficacious treatment, but our view is that the treatment could be improved further by achieving greater traction on the mechanisms. Encouragingly, treatment effects were not hindered by factors often considered to be problematic for therapy, such as working memory, illicit drug use, and anger. The absence of moderators is a promising indicator for the general applicability in clinical services of the cognitive therapy.

The principal limitation of our trial is the small sample size when comparing two active treatments. There will be less precision in effect size estimates, limited power to detect moderate differences, and even less power to detect differences in dichotomous variables. 150 patients were not recruited as planned, but this factor was adequately compensated by a higher follow-up rate than conservatively estimated. We did not do subgroup analyses by gender as we had not previously found this characteristic to moderate our treatment outcomes.^10^ There were slight differences between the two trial groups in the number of sessions attended, which was a result of the reality of comparatively less uptake of befriending sessions by patients. Befriending was a popular intervention, with high uptake, but there was even greater engagement with the Feeling Safe Programme. We sought to balance the number of sessions provided for each intervention, but many patients did not want the additional befriending sessions offered (ie, they received a maximum dose), whereas many patients receiving the Feeling Safe Programme would have liked an even greater number of sessions to that provided. The imbalance in session number might plausibly account for a degree of the difference in outcomes, although this speculation is a moot point given that many patients had reached their limit for the non-specific intervention and it is not known what would have occurred if the dose had been increased beyond that, and balanced against this potential bias is that a greater number of patients allocated to befriending received additional psychological interventions during the trial, which could have lowered the effect size estimate of the Feeling Safe Programme. The trial was done in one centre, and there were few participants from ethnic minority groups, which could limit the generalisability of our results. Our study provides grounds for both a multicentre trial and for further refinement of the intervention methods.

## Data sharing

Deidentified participant data will be available in anonymised form from the corresponding author (DF) on reasonable request (including a study outline), subject to review. The trial protocol is published. Data analytic plans and full statistical reports are available in the appendix.

## Declaration of interests

DF reports grants from National Institute for Health Research, Medical Research Council, and International Foundation, and is a founder and non-executive board director of a University of Oxford spin-out company, Oxford VR. DF has also written popular science, self-help, and academic books about paranoia with several publishers for which royalties are received. RE reports grants from the National Institute for Health Research. All other authors declare no competing interests.
