# Supplementary material for: Comparison of a theoretically driven cognitive therapy (the Feeling Safe Programme) with befriending for the treatment of persistent persecutory delusions: a parallel, single-blind, randomised controlled trial
Source: Lancet Psychiatry. 2021 Aug;8(8):696–707. doi: 10.1016/S2215-0366(21)00158-9 (PMC8311296; doi:10.1016/S2215-0366(21)00158-9)
Supplement: Supplementary appendix [file mmc1.pdf]

THE LANCET
Psychiatry

Supplementary appendix

This appendix formed part of the original submission and has been peer reviewed. We post it as supplied by the authors.

Supplement to: Freeman D, Emsley R, Diamond R, et al. Comparison of a theoretically driven cognitive therapy (the Feeling Safe Programme) with befriending for the treatment of persistent persecutory delusions: a parallel, single-blind, randomised controlled trial. *Lancet Psychiatry* 2021; published online July 8. http://dx.doi.org/10.1016/S2215-0366(21)00158-9.

Study protocol

Trial Title: Psychological support for fears about other people: A comparison of the Feeling Safe Programme to befriending.

Ethics Ref: 15/SC/0508

Date and Version No: 9th December 2015, Version 2.0

Chief Investigator: Daniel Freeman (University of Oxford)

Investigators: Felicity Waite (University of Oxford), Richard Emsley (University of Manchester), Graham Dunn (University of Manchester), David Kingdon (University of Southampton), Linda Davies (University of Manchester), Ray Fitzpatrick (University of Oxford)

Sponsor: University of Oxford

Funder: NHS National Institute of Health Research (NIHR)

Chief Investigator Signature:

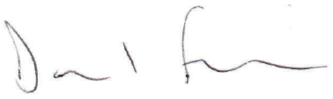

Please declare any/no potential conflicts of interest: None

Confidentiality Statement

This document contains confidential information that must not be disclosed to anyone other than the Sponsor, the Investigator Team, host organisation, and members of the Research Ethics Committee, unless authorised to do so.

TABLE OF CONTENTS

1. KEY TRIAL CONTACTS 4

2. SYNOPSIS 5

3. ABBREVIATIONS 7

4. BACKGROUND AND RATIONALE 8

5. OBJECTIVES AND OUTCOME MEASURES 9

6. TRIAL DESIGN 11

7. PARTICIPANT IDENTIFICATION 12

7.1. Trial Participants 12

7.2. Inclusion Criteria 12

7.3. Exclusion Criteria 13

8. TRIAL PROCEDURES 13

8.1. Recruitment 13

8.1. Screening and Eligibility Assessment 13

8.2. Informed Consent 13

8.3. Randomisation and blinding 14

8.4. Baseline Assessments 14

8.5. Subsequent Visits 15

8.6. Discontinuation/Withdrawal of Participants from Trial Treatment 16

8.7. Definition of End of Trial 16

9. PSYCHOLOGICAL TREATMENTS 16

9.1. Description 16

9.2. Compliance with Trial Treatments 16

10. SAFETY REPORTING 16

10.1. Definitions .... **Error! Bookmark not defined.**

10.2. Causality .... **Error! Bookmark not defined.**

10.3. Procedures for Recording Adverse Events 16

10.4. Reporting Procedures for Serious Adverse Events .... **Error! Bookmark not defined.**

10.5. SUSAR Reporting .... **Error! Bookmark not defined.**

10.6. Safety Monitoring Committee 17

We will form a Data Monitoring and Ethics Committee (DMEC) with an independent clinician chair, independent statistician, and further independent clinician. 17

11. STATISTICS 17

12. DATA MANAGEMENT 19

12.1. Source Data 19

12.2. Access to Data 19
12.3. Data Recording and Record Keeping 19
13. QUALITY ASSURANCE PROCEDURES 19
14. ETHICAL AND REGULATORY CONSIDERATIONS 19
14.1. Declaration of Helsinki 19
14.2. Guidelines for Good Clinical Practice 20
14.3. Approvals 20
14.4. Reporting 20
14.5. Participant Confidentiality 20
14.6. Expenses and Benefits 20
14.7. Other Ethical Considerations 20
15. FINANCE AND INSURANCE 21
15.1. Funding 21
15.2. Insurance 21
16. PUBLICATION POLICY 21
17. REFERENCES 21
18. APPENDIX A: SCHEDULE OF PROCEDURES 25
19. APPENDIX B: AMENDMENT HISTORY 26

1. KEY TRIAL CONTACTS

**Chief Investigator**	Prof Daniel Freeman, Department of Psychiatry, University of Oxford, Warneford Hospital, Oxford OX3 7JX. Email: Daniel.freeman@psych.ox.ac.uk . Tel. 01865 226490
**Sponsor**	Heather House, Clinical Trials Research Governance, University of Oxford, Joint Research Office, Block 60, Churchill Hospital, Oxford OX3 7LE. Email: ctrg@admin.ox.ac.uk. Tel 01865 57228
**Clinical Trials Unit**	Oxford Cognitive Health and Neuroscience Clinical Trials Unit, University of Oxford, Warneford Hospital, Oxford OX3 7JX. Email: ochnctu@psych.ox.ac.uk. Tel 01865 226465
**Statistician**	Dr Richard Emsley and Prof Graham Dunn, Centre for Biostatistics, Institute of Population Health, University of Manchester, Jean McFarlane Building, Oxford Road, Manchester, M13 9PL. Email: Richard.emsley@manchester.ac.uk. Tel 0161 2755205

2. SYNOPSIS

Trial Title	Psychological support for fears about other people: A comparison of the Feeling Safe Programme to befriending.	
Clinical Phase	II	
Trial Design	Randomisation to the Feeling Safe Programme (a cognitive intervention) or befriending (social support). Standard NHS care continues as usual in both arms.	
Trial Participants	Patients with persistent persecutory delusions (despite receiving treatment from services) in the context of non-affective psychosis (typically schizophrenia diagnosis).	
Planned Sample Size	150 patients in the trial.<br>(10 family members/partners and 10 mental health staff in a qualitative sub-sample)	
Treatment duration	6 months	
Follow up duration	6 months	
Planned Trial Period	12 months	
	Objectives	Outcome Measures
Primary	Test improvements in persistent persecutory delusions by treatment type.	Psychotic Symptoms Rating Scale – Delusions (Haddock et al., 1999)
Secondary	1. Test clinical improvements by treatment type in well-being, patient satisfaction, activity levels, paranoia, suicidal ideation, and overall psychiatric symptoms.<br><br>2. Test moderation and mediation of treatment effects.<br><br>3. Carry out a cost-effectiveness analysis.	1. Warwick-Edinburgh Mental Well-being Scale (Tennant et al., 2007); the CHOICE (Greenwood et al., 2010); step count; time-budget (Jolley et al., 2006); GPTS (Green et al., 2008); Columbia-Suicide Severity Rating Scale (Posner et al., 2011);, SPEQ (Ronald et al., 2014); BDI-II (Beck et al., 1996); belief flexibility (Waller et al., 2015); LTCQ (Potter et al., 2015); DAR-5 (Forbes et al., 2014).<br><br>2. Penn State Worry Questionnaire (Meyer et al., 1990); Brief Core Schema Scales (Fowler et al., 2006); Insomnia Severity Scale (Bastien et al., 2001); safety behaviours (Freeman et al., 2001); jumping to conclusions (Garety et al., 2005).<br><br>3. EQ-5D-5L (see http://www.euroqol.org/); Client Service Receipt Inventory (CSRI; Beecham & Knapp, 1992).

Scientific summary

Background: Persecutory delusions (strong unfounded fears that others intend harm to them) occur in over 70% of patients with schizophrenia. This major psychotic experience is a key treatment target. The delusion has substantial impact for patients (and families), including suicidal ideation, isolation, reduced activity, and hospital admission. Schizophrenia is among the top ten disorders in terms of burden of illness, disability and societal and health costs worldwide. The total annual cost to the public sector in England is over £7 billion. Life expectancy is 15-20 years shorter for people with these problems.

Approximately half of patients do not respond adequately to the first line treatment, medication. Residual problems are very common. The 2014 National Audit of Schizophrenia calls for NHS Trusts to increase access to evidence-based psychological interventions. But trials show that the psychological treatment needs improvement. A much more efficacious, easily useable intervention is urgently required.

The chief investigator and colleagues have developed a rigorously tested theoretical model of persecutory delusions. At the core of the delusion is a belief of being unsafe, developed in the context of genetic and environmental risk, that is maintained by a number of factors including disrupted sleep, worrying, negative beliefs about the self, reasoning biases, and avoidance of others. The delusion diminishes if maintaining factors are successfully reduced and the patient is then enabled to relearn that they are safe. The chief investigator and colleagues have been developing brief intervention modules each targeting a maintaining factor. One by one the modules have been successfully evaluated. These modules need to be tested together as a full treatment (20 sessions). A case series has already established the feasibility and potential benefits of the full treatment, called The Feeling Safe Programme.

Aims: The target is recovery in persistent delusions for 50% of patients. The key question asked is: Does the Feeling Safe Programme lead to greater recovery in persecutory delusions, psychological well-being, and activity levels compared to befriending (i.e. controlling for the extra time spent with a therapist)?

Method: The study is a randomised controlled trial for 150 patients who have persecutory delusions despite previous treatment i.e. the group most at need. Patients will be randomised to The Feeling Safe Programme or befriending (both provided over six months). Medication prescription will continue as usual. Assessments, by a rater blind to allocation, will be conducted at 0, 6 (post treatment), and 12 months. All main analyses will be intention-to-treat. A health economic evaluation is included. A small number of qualitative interviews will also be carried out with patients, family members, and NHS staff to determine views of the intervention and implementation. The trial is funded by the NHS National Institute for Health Research.

3. ABBREVIATIONS

AE	Adverse event
AR	Adverse reaction
CI	Chief Investigator
CRF	Case Report Form
CRO	Contract Research Organisation
CT	Clinical Trials
CTA	Clinical Trials Authorisation
CTRG	Clinical Trials and Research Governance
DMC/DMSC	Data Monitoring Committee / Data Monitoring and Safety Committee
DSUR	Development Safety Update Report
GCP	Good Clinical Practice
GP	General Practitioner
ICF	Informed Consent Form
ICH	International Conference on Harmonisation
IMP	Investigational Medicinal Product
IRB	Independent Review Board
MHRA	Medicines and Healthcare products Regulatory Agency
NHS	National Health Service
NRES	National Research Ethics Service
PI	Principal Investigator
PIL	Participant/ Patient Information Leaflet
R&D	NHS Trust R&D Department
REC	Research Ethics Committee
SAE	Serious Adverse Event
SAR	Serious Adverse Reaction
SDV	Source Data Verification
SMPC	Summary of Medicinal Product Characteristics
SOP	Standard Operating Procedure
SUSAR	Suspected Unexpected Serious Adverse Reactions
TMF	Trial Master File
TSG	Oxford University Hospitals Trust / University of Oxford Trials Safety Group

4. BACKGROUND AND RATIONALE

The clinical problem

Persecutory delusions, a central problem in schizophrenia, are unfounded beliefs that others are trying to harm the person. Almost half of individuals with persecutory delusions have major depression (Vorontsova et al., 2013). Persecutory delusions predict serious violence (Coid et al., 2013), suicide (Hor & Taylor, 2010), and hospital admission (Castle et al., 1994). It is well-recognised that treatments for persecutory delusions need significant improvement. The first line treatment, medication, has effect sizes (standardised mean differences) varying between 0.33 and 0.88 (median=0.44) (Leucht et al., 2013), with problems of major side-effects, poor compliance, and residual symptoms. In a recent review, Kennedy et al (2014) found that 'almost 60% of patients failed to achieve response after 23 weeks on antipsychotic drug therapy.' Similarly, meta-analysis for first generation psychological treatment (when added to medication) indicates an effect size of 0.36 for delusions (van der Gaag et al., 2014). Psychological treatment is a valued treatment choice for patients but there are also problems of availability. The National Audit of Schizophrenia (2014) states: "It is clear that the numbers of service users having access to, and actually receiving, these types of intervention remain very low. This needs to be addressed and has significant funding implications." Using advances in the understanding of the causes of persecutory delusions, our team have been developing a new targeted psychological treatment - called 'The Feeling Safe Programme' – that is aimed to improve efficacy and deliverability.

The translational studies leading to the trial

At the core of a persecutory delusion is the belief that the person is unsafe (Freeman et al, 2002). The latest research shows that the heritability of paranoid thoughts is 50% (Zavos et al., 2014), indicating genetic and environmental risk leading to such fears. Once developed, the beliefs concerning danger are maintained by a number of factors (Freeman & Garety, 2014). For example, worry brings implausible ideas to mind, keeps them there, and exacerbates the distress; reasoning biases prevent the processing of alternative explanations; negative self-beliefs lead the person to feel inferior and vulnerable; and avoidance (a type of 'safety behaviour') prevents the person receiving disconfirmatory evidence that they are safe. Therefore treatment needs to target the maintenance factors, before helping the patient to go into everyday situations and relearn that they are safe.

The CI and colleagues have been developing brief treatment modules targeting each of the five key maintenance factors: disrupted sleep, negative self-beliefs, worry, reasoning biases, and avoidance behaviours. Each element has been evaluated separately to show that it merits inclusion in a full treatment. Ten studies have now been carried out. The strongest test has been for reducing worry. A randomised controlled trial ('The Worry Intervention Trial') with 150 patients with persistent persecutory delusions was recently completed (Freeman, Dunn et al., 2015). This had blind ratings and a 95% follow-up rate. Targeting worry, in just six sessions, significantly reduced both worry and the persecutory delusions (both effect sizes=0.5, $p<.001$). A mediation analysis showed that two thirds of the reductions in the delusions were due to reductions in worry. There were also significant increases in psychological well-being and reductions in overall psychiatric symptoms. There was no evidence of adverse events. The CI

and colleagues have also completed a pilot RCT with 30 patients with persistent persecutory delusions targeting, in six sessions, negative self-beliefs (Freeman, Pugh et al, 2014). Ratings were blind and 100% of patients were followed up. Treatment resulted in reductions in negative self-beliefs (d=0.24) and delusions (d=0.6), and improvements in psychological well-being (d=1.2). Again, there was no evidence of any adverse events associated with treatment. Two recent randomised controlled studies have also shown the benefits of reducing reasoning biases in patients with delusions (Garety et al., 2014; Waller et al., 2015). A case series indicated that persecutory delusions may be reduced by targeting insomnia (Myers et al, 2011), and we have recent data from a pilot randomised controlled trial that sleep can be improved significantly (effect size = 1.9) in patients with delusions and hallucinations and that there may be associated improvements in paranoia (effect size=0.2) (Freeman et al, in revision). A current study by the CI and team also addresses the best method of patients learning that they are safe. It is showing that going into feared situations (i.e. reducing avoidance) while dropping unhelpful safety behaviours that prevent full processing of disconfirmatory evidence reduces delusions to a much greater extent than exposure alone (UKCRN ID 12951).

All these elements have now been put together as a full intervention, called The Feeling Safe Programme, delivered in 20 sessions over six months. The feasibility of this treatment has been recently established in a case series, and there have been indications of clear clinical benefits for the patients (UKCRN ID 16387).

The central aim now is to test this new theoretically-driven treatment for persecutory delusions. The target group is those at most need: patients whose delusions have not responded to current treatment. It is anticipated that the Feeling Safe Programme will lead to 50% of patients having recovery in persistent persecutory delusions. We will test the intervention against an equal time receiving befriending. Befriending has benefits for patients with psychosis, in the short-term comparable to first generation psychological therapy for psychosis (e.g. Sensky et al., 2000; Li et al., 2015). This choice of comparison allows us to determine whether the Feeling Safe Programme has benefits over and above the extra time spent with a therapist, which is important to determine for future training needs and service provision.

5. OBJECTIVES AND OUTCOME MEASURES

Objectives	Outcome Measures	Timepoint(s) of evaluation of this outcome measure (if applicable)
**Primary Objective**<br>To test the following hypotheses:<br>1. The Feeling Safe Programme will lead to lower levels of persecutory delusions compared to befriending.	The primary outcome measure will be conviction in the persecutory delusion (using a 0–100% scale), assessed within the Psychotic Symptoms Rating Scale - Delusions (Haddock et al., 1999). We will test rates of recovery in the delusion (defined as conviction falling below 50%) and dimensional change in conviction levels.	0, 6mths, 12mths<br>(Primary endpoint: 6mths).

**Secondary Objectives**<br>To test the following hypotheses:<br><br>1. The Feeling Safe Programme will lead to improved well-being, patient satisfaction, and activity levels compared to befriending.<br><br>2. The Feeling Safe Programme will lead to lower levels of paranoia, total delusion severity, suicidal ideation, and psychiatric symptoms.<br><br>3. The benefits will persist at follow-up.	1. Psychological well-being will be assessed by the Warwick-Edinburgh Mental Well-being Scale (Tennant et al., 2007). Patient satisfaction will be assessed using the CHOICE, a service user-led outcome measure (Greenwood et al., 2010). Activity levels will be assessed using step count and a time-budget measure (Jolley et al., 2006).<br><br>2. We will also include measures of overall paranoia (GPTS; Green et al, 2008), overall delusion severity (PSYRATS Delusions total), suicidal ideation (Columbia-suicide Severity Rating Scale; Posner et al, 2011), and overall psychiatric symptoms, (SPEQ – hallucinations and anhedonia subscales; Ronald et al, 2014); BDI-II (Beck et al., 1996); belief flexibility (Waller et al, 2015); LTCQ (Potter et al, 2015); DAR-5 (Forbes et al, 2014).	0, 6mths, 12mths (primary endpoint: 6mths).
**Tertiary Objectives**<br>To test the following hypotheses:<br><br>1. Changes in emotional and reasoning processes will mediate the change in delusions.<br><br>2. Working memory and illicit drug use will moderate treatment effects.<br><br>3. The Feeling Safe Programme will be cost-effective.<br><br>We will also carry out qualitative interviews with a small number of patients, family members, and	1. For mediation we will include: Penn State Worry Questionnaire (Meyer et al., 1990); Brief Core Schema Scales (Fowler et al., 2006); Insomnia Severity Index (Bastien et al., 2001); Safety Behaviours Questionnaire (Freeman et al, 2001), and jumping to conclusions (Garety et al., 2005).<br><br>2. We will include as moderators: working memory (Wechsler, 2007) and illicit drug use (Marsden et al., 1998).<br><br>3. We will record all service use, and other relevant health economic data, using an adapted version of the Client Service Receipt Inventory (CSRI; Beecham & Knapp, 1992). Quality of life will be assessed with the EQ-5D-5L (see http://www.euroqol.org/).<br><br>Qualitative interviews will be conducted. The questions to be asked will be	0, 6mths, 12mths

mental health staff about the Feeling Safe Programme. The purpose is in order to refine the intervention and ready it for potential NHS implementation.	developed with the McPin Foundation and a Patient Advisory Group, but focus on the topics of how things were before the intervention, the experience of the intervention, and the subsequent effects. Staff views will be on potential implementation issues.	12 months

6. TRIAL DESIGN

The design is a parallel group randomised controlled trial with single blind assessment to test whether the new psychological treatment will reduce persecutory delusions more effectively than befriending (an attention control condition) (see Figure 1). Standard care will be measured but remain as usual in both groups. Assessments will be carried out at 0, 6 (post treatment) and 12 months. The trial will be registered, the protocol submitted for publication, and a Project Advisory Group, a Data Monitoring and Ethics Committee, and a Patient and Public Advisory Group set-up.

Figure 1. Trial flow diagram.

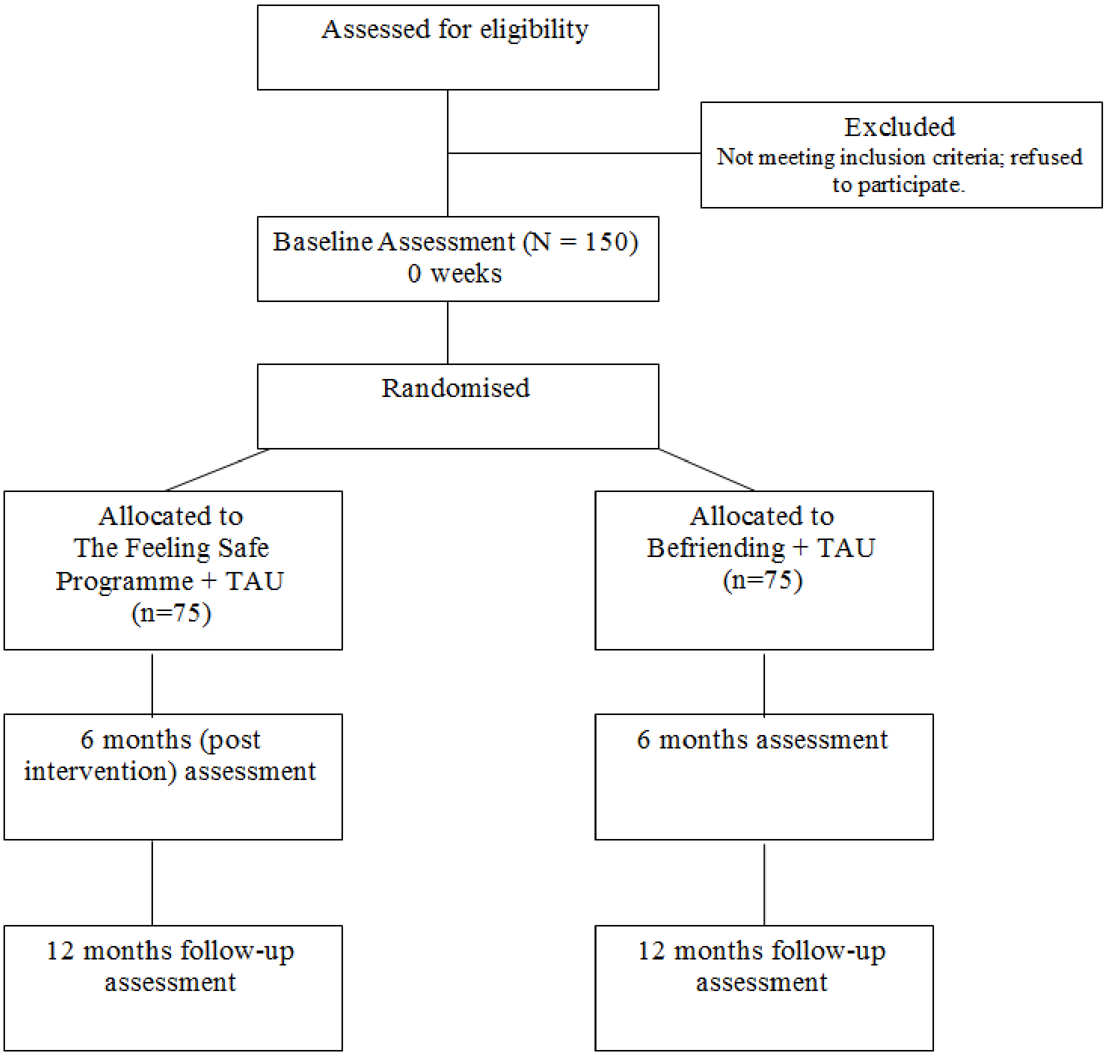

7. PARTICIPANT IDENTIFICATION

7.1. Trial Participants

Participants with persistent persecutory delusions in the context of non-affective psychosis.

7.2. Inclusion Criteria

- Participant is willing and able to give informed consent for participation in the trial.
- Male or Female, aged 16 years or above.
- Persistent (at least 3 months) persecutory delusion (as defined by Freeman & Garety, 2000), held with at least 60% conviction
- Primary diagnosis of schizophrenia-spectrum psychosis (non-affective psychosis).

7.3. Exclusion Criteria

The participant may not enter the trial if any of the following apply:

- Current receipt of another psychological therapy.
- Insufficient comprehension of English.
- Primary diagnosis of alcohol, drug, or personality disorder.
- In forensic settings.
- Organic syndrome.
- Learning disability.

8. TRIAL PROCEDURES

The schedule of procedures is summarised in the appendix.

8.1. Recruitment

Referrals to the trial will be sought from the relevant clinical teams in the mental health Trusts. If a patient is willing to be approached by the research team then information about the trial will be provided and screening conducted. All suitable patients will be given at least 24hours to consider taking part in the trial, although in practice it is typically a week. Recruitment is primarily expected to occur from Oxford Health NHS Foundation Trust, but we will also recruit from two neighbouring Trusts: Northamptonshire Healthcare NHS Foundation Trust and Berkshire Healthcare NHS Foundation Trust.

8.1. Screening and Eligibility Assessment

The key screening with the patient is for the presence of a current persecutory delusion. This is established in a brief discussion with the patient. The clinical diagnosis is provided by the Trust clinical team.

8.2. Informed Consent

Written and verbal versions of the Participant Information and Informed Consent will be presented to the participants detailing no less than: the exact nature of the trial; what it will involve for the participant; the implications and constraints of the protocol; the known side effects and any risks involved in taking part. It will be clearly stated that the participant is free to withdraw from the trial at any time for any reason without prejudice to future care, without affecting their legal rights and with no obligation to give the reason for withdrawal.

The participant will be allowed as much time as wished to consider the information, and the opportunity to question the Investigator, their mental health team or other independent parties to decide whether they will participate in the trial. Written Informed Consent will then be obtained by means of participant dated signature and dated signature of the person who presented and obtained the Informed Consent. The person who obtained the consent must be suitably qualified and experienced, and have been authorised to do so by the Chief Investigator. A copy of the signed Informed Consent will be given to the participant. The original signed form will be retained at the trial site.

8.3. Randomisation and blinding

Randomisation will occur after completion of the baseline assessment. An online randomisation system will be written by the Oxford Cognitive Health and Neurosciences Clinical Trials Unit. Randomisation using a permuted blocks algorithm, with randomly varying block size, will be stratified by therapist and severity of delusion (moderate (60-89% conviction)/high (90%+ conviction)). (Therapists will provide both interventions in order to reduce the confounding of therapist effects and increase statistical power.)

The trial assessors will be blind to group allocation, but the patients and trial therapists will not be (they cannot be blinded to what psychological treatment is delivered or received). The trial therapists will inform patients of the randomisation outcome, so that the research assessors remain blind to group allocation. Precautionary strategies to prevent breaks of blind included: the therapist and assessor considering room use and booking arrangements; patients being reminded by the assessor not to talk about treatment allocation; and, after the initial assessment, the assessor not looking at the patient's clinical notes. If an allocation is revealed between assessment sessions then re-blinding will occur using another assessor.

8.4. Baseline Assessments

The measures have been successfully used in the pilot studies. Assessments are in person, typically in clinic rooms or at home (for patients who find it difficult to leave their residence). Basic demographic and clinical data will be collected (e.g. age, gender, ethnicity, clinical diagnosis). The primary outcome measure will be conviction in the persecutory delusion (using a 0–100% scale), assessed within the Psychotic Symptoms Rating Scale-Delusions scale (Haddock et al, 1999). Recovery is defined as the conviction in the delusional belief falling below 50% i.e. there is greater doubt than belief in the delusion. Conviction greater than 50% is a standard definition of the presence of a delusion (e.g. Hartley et al, 2012), although typically such beliefs are held with much greater certainty. For example, in our Feeling Safer Programme pilot study (n=12) the initial conviction levels in the delusions showed a mean of 90% (SD=17); in a previous study with 100 patients with delusions the mean conviction rating was 82% (SD = 20) (Freeman et al, 2004); while in the Worry Intervention Trial it was found that at baseline half of the 150 patients had 100% conviction in the persecutory delusions (Freeman et al, 2015). Reductions in conviction are highly associated with improvements in well-being (p=.001). Although recovery is the target it is still a key interest to examine change in dimensional scores. We expect a higher proportion than 50% to have benefits from the intervention.

Psychological well-being will be assessed by the Warwick-Edinburgh Mental Well-being Scale (Tennant et al, 2007), quality of life by the EQ-5D-5L (see http://www.euroqol.org/), and patient satisfaction using the CHOICE, a service user-led outcome measure (Greenwood et al, 2010). Activity levels will be assessed using step count (a measure of the number of steps taken measured on a small watch-like device worn on the wrist) and a time-budget measure (Jolley et al, 2006). We will also include measures of overall paranoia (Green et al, 2008), suicidal ideation (Columbia-Suicide Severity Rating Scale; Posner et al, 2011), and overall psychiatric symptoms (assessed using symptom specific measures: Specific psychotic experiences questionnaire – hallucinations and anhedonia subscales (Ronald et al., 2014); Beck depression inventory (BDI-II; Beck et al., 1996); belief flexibility (single item) (Waller et al, 2015); Long-term conditions questionnaire (LTCQ: Potter et al., 2015); Dimensions of Anger Reactions-5 (DAR-5; Forbes et al., 2014)..

As well as advancing treatment, evidence of moderators and mechanisms of action will inform theories of persecutory delusions and future stratified medicine approaches. We will include as moderators: working memory (Wechsler, 2007) and illicit drug use (Marsden et al, 1998). For mediation we will include: Penn

State Worry Questionnaire (Meyer et al, 1990); Brief Core Schema Scales (Fowler et al, 2006); Insomnia Severity Index (Bastien et al., 2001); safety behaviours (Freeman et al, 2001), and jumping to conclusions (Garety et al., 2005). We will record service use, and other relevant health economic data, using the Client Service Receipt Inventory (CSRI; Beecham & Knapp, 1992).

Included in the baseline assessment only will be assessment of two issues which are not often explored within this population but which are of clinical interest. These relate to the onset of persecutory beliefs and body image. A checklist of events preceding the onset of persecutory beliefs has been generated by the research team. Body image will be assessed using the Body-esteem scale for adolescents and adults (BESAA; Mendelson, Mendelson & White, 2001), a single item from the SCOFF questionnaire (Morgan, Reid & Lacey, 1999) relating to control over eating and a calculation of Body Mass Index based on height and weight measurements.

8.5. Subsequent Visits

There are two further trial assessments: 6months and 12months. The assessments at these time-points are: Psychotic Symptoms Rating Scale-Delusions (Haddock et al, 1999); Warwick-Edinburgh Mental Well-being Scale (Tennant et al, 2007); EQ-5D-5L (see http://www.euroqol.org/); the CHOICE (Greenwood et al, 2010); step count; time-budget (Jolley et al, 2006); GPTS (Green et al, 2008); Columbia-suicide Severity Rating Scale (Posner et al, 2011); SPEQ – hallucinations and anhedonia subscales (Ronald et al., 2014); Beck depression inventory (BDI-II; Beck et al., 1996);); belief flexibility (single item) (Waller et al., 2015); Long-term conditions questionnaire (LTCQ: Potter et al, 2015); Dimensions of Anger Reactions-5 (DAR-5; Forbes et al., 2014).); Penn State Worry Questionnaire (Meyer et al, 1990); Brief Core Schema Scales (Fowler et al, 2006); Insomnia Severity Index (Bastien et al, 2001); safety behaviours (Freeman et al, 2001); jumping to conclusions (Garety et al, 2005); Client Service Receipt Inventory (CSRI; Beecham & Knapp, 1992).

Qualitative studies

For a subsample of trial patients we will also carry out qualitative interviews. Patients (n=10) and family members/partners chosen by patients (n=10) will be interviewed, by people with lived experience of psychosis employed by the McPin Foundation, about the Feeling Safe Programme. This will be used to help refine the final version of the treatment and to incorporate patient views into a subsequent dissemination package. Interpretative Phenomenological Analysis will be used, which focuses on understanding the lived experience. Analysis will follow the procedure described by Smith et al (2009). The analysis will be carried out by the McPin Foundation. The McPin Foundation (http://mcpin.org/) "exists to transform mental health research by putting the lived experience of people affected by mental health problems at the heart of research methods and the research agenda". They have, for example, been commissioned by NHS England to deliver a service user evaluation of the six IAPT (Improving Access to Psychological Therapy) pilot sites for SMI (Severe Mental Illness). Mental health staff (n=10) will also be interviewed by a member of the trial team. This will be used to inform potential implementation of the treatment in the NHS. Views will be obtained on both the direct delivery of the Feeling Safe Programme and the changes in service required. The framework method, a type of thematic analysis, will be used for this analysis of qualitative data (Gale et al, 2013). Separate consent forms will be used for the qualitative studies, and potential participants will be chosen at random during the course of approximately a year. The exact questions asked will be developed in consultation with the trial Patient and Public Advisory Group over the first months of the trial.

8.6. Discontinuation/Withdrawal of Participants from Trial Treatment

Each participant has the right to withdraw from the trial at any time. Withdrawal from the trial will not result in exclusion of the previously collected data for that participant from analysis (unless this is specifically requested).

8.7. Definition of End of Trial

The end of trial is the date of the last assessment of the last participant.

9. PSYCHOLOGICAL TREATMENTS

9.1. Description

Feeling Safe Programme. After the assessment, the patient is offered a menu of appropriate treatment modules. Typically three to four modules are completed, based upon the assessments and patient preference. Direct behavioural tests to relearn safety are typically carried out in the final sessions. The range of modules offered are: improving sleep, reducing worry, increasing self-confidence, improving reasoning processes, and behavioural tests for reducing fear beliefs. These modules are delivered in a one-to-one format, with supportive telephone calls or texts between sessions. Sessions typically last one hour, but this is flexible. Treatment is offered over six months. In the pilot the typical number of sessions provided was 20.

Befriending. This will follow a protocol devised by David Kingdon (a trial investigator), which has previously been used under his supervision in two large clinical trials for patients with psychosis over 20 sessions (e.g. Sensky et al, 2000; Li et al, 2015). Essentially the aim is to simulate how a good friend would respond, involving: a general focus on non-threatening topics (although patients are not actively dissuaded from talking about concerns); non-confrontation; empathy; and supportiveness.

9.2. Compliance with Trial Treatments

Both treatments will be provided by either research team clinical psychologists (with honorary Trust clinical contracts) or other mental health staff, under the weekly supervision of the trial team. For both treatments the number of sessions and length are recorded, sessions are taped when patients are agreeable, and tapes are rated for fidelity and competence. Patient beliefs about the potential effectiveness of the intervention that he or she receives will be assessed after two sessions with the Credibility/expectancy questionnaire (Devilly & Borkovec, 2000), and therapeutic empathy will also be assessed with a patient questionnaire (Burns & Nolen-Hoeksema, 1992).

10. SAFETY REPORTING

10.1. Definition of Serious Adverse Events

A serious adverse event is any untoward medical occurrence that:

- results in death
- is life-threatening

- requires inpatient hospitalisation or prolongation of existing hospitalisation
- results in persistent or significant disability/incapacity
- consists of a congenital anomaly or birth defect.

Other 'important medical events' may also be considered serious if they jeopardise the participant or require an intervention to prevent one of the above consequences. (We note that admissions to psychiatric hospital are expected in this client group, and are not considered an adverse event.)

NOTE: The term "life-threatening" in the definition of "serious" refers to an event in which the participant was at risk of death at the time of the event; it does not refer to an event which hypothetically might have caused death if it were more severe.

10.2. Reporting Procedures for Serious Adverse Events

All serious adverse events that come to our attention are reviewed by the study team, and are sent to the Chair of the Data Monitoring and Ethics Committee for a decision on whether it is potentially related to the intervention or trial procedures. A serious adverse event (SAE) occurring to a participant will be reported to the REC that gave a favourable opinion of the study where the event was 'related' (resulted from administration of any of the research procedures) and 'unexpected' in relation to those procedures. Reports of related and unexpected SAEs will be submitted within 15 working days of the Chief Investigator becoming aware of the event, using the HRA report of serious adverse event form (see HRA website).

This patient group has a higher rate than the general population for the occurrence of adverse events. For example, suicide attempts occur at a higher rate, as do physical health problems. However these are still rare in our studies. For example, in our Worry Intervention Trial with 150 patients with persistent persecutory delusions followed for six months, no patients died or were admitted to secure units during the study but there were six suicide attempts (two in the psychological treatment intervention group, and four in the standard care control group), and two serious violent incidents (one in each group). None were deemed by the DMEC to be related to the trial. Serious adverse events related to psychological reactions (i.e. SAR or SUSAR) are extremely rare (and have not occurred in our studies).

In order to monitor this, we check medical notes at the end of a patient's participation for the following events pre-specified as adverse: 1. All deaths. 2. Suicide attempts. 3. Serious violent incidents. 4. Admissions to secure units. 5. Formal complaints about therapy. We also, of course, record any such event that we become aware of during a patient's participation. We note that admissions to *psychiatric* hospital are expected in this client group, and are not considered an adverse event.

10.3. Safety Monitoring Committee

We will form a Data Monitoring and Ethics Committee (DMEC) with an independent clinician chair, independent statistician, and further independent clinician.

11. STATISTICS

A full statistical analysis plan will be written by the trial statisticians (RE, GD) prior to any analysis being undertaken. We will report data in line with the Consolidated Standards of Reporting Trials (CONSORT) 2010 Statement showing attrition rates and loss to follow-up. All analyses will be carried out using the intention to treat principle with data from all participants included in the analysis including those who do not complete therapy. Every effort will be made to follow up all participants in both arms for research assessments.

Analysis will be conducted in Stata version 14. Descriptive statistics within each randomised group will be presented for baseline values. These will include counts and percentages for binary and categorical variables and means and standard deviations, or medians with lower and upper quartiles, for continuous variables, along with minimum and maximum values and counts of missing values. There will be no tests of statistical significance or confidence intervals for differences between randomised groups on any baseline variable.

Descriptive statistics will be used to summarize assessments of feasibility and acceptability in terms of recruitment, drop-out and completeness of therapy.

The primary hypothesis for change in the primary outcome measure, conviction in the persecutory delusion (using a 0–100% scale) at 6 months, will be analysed using a linear regression model allowing for the baseline measurement of outcome, severity of delusion, therapist and treatment assignment as fixed effects. To compare rates of recovery (scores falling below 50%), we will use logistic regression models instead of linear models. Secondary outcome measures will be analysed using the same modelling approach. This includes analysis of the primary outcome and secondary outcomes at 12 months.

The mediation analysis will investigate putative mediational factors using modern causal inference methods. This involves using parametric regression models to test for mediation of the Feeling Safe Intervention on outcome through the putative mediators. Analyses will adjust for baseline measures of the mediator, outcomes, and possible measured confounders. We will include repeated measurement of mediators and outcomes to account for classical measurement error and baseline confounding, and where feasible, use instrumental variable methods (baseline covariate by randomization interactions as potential instruments) to investigate the sensitivity of the estimates to these problems and that of unmeasured confounding.

Moderators will be assessed separately by repeating the primary analysis models and including interaction terms between the randomised intervention and each moderator. The coefficient of the interaction term is a measure of whether the treatment effect differs between levels of the moderator.

Missing data on individual measures will be pro-rated if more than 90% of the items are completed; otherwise the measure will be considered as missing. We will check for differential predictors of missing outcomes by comparing responders to non-responders on key baseline variables. Any significant predictors will be included in the analysis models. This accounts for missing outcome data under a missing at random assumption, conditional on the covariates included in the model. As a sensitivity analysis, we will assess whether treatment adherence is associated with missing data, and if it is associated, use inverse probability weights or multiple imputation to compare results.

An economic evaluation, adopting a health and social care perspective, will be undertaken by the health economist (LD) to assess the cost-effectiveness, both at one-year and over the patient's lifetime, of the intervention to determine the incremental cost per Quality-Adjusted Life Year (QALY) gained.

For a recovery rate in delusions of 50% in the Feeling Safe Programme, compared to 20% with befriending, a study will have over 90% power with 60 patients in each arm. The trial will however gain greater power by also examining change in delusion dimensional scores. If the standardised effect of the new intervention compared to befriending were smaller than 10 percentage points on the conviction scale (0-100%) (d=0.5) then we would not consider further development of the intervention to be worth pursuing. If the true effect size were this ten point difference (SD=20) then a two-sample t-test, with a two-sided significance level of 0.05, would have 80% power to detect a statistically significant effect with outcome data available for 64 participants per randomised arm. We aim to recruit 75 per arm. This conservatively allows for drop-out of 15%. Allowing for stratum membership and baseline levels of the measures in a more refined analysis of covariance will increase both statistical power and precision.

12. DATA MANAGEMENT

12.1. Source Data

We keep data from the assessments, collected on paper from the patient interviews. All documents will be stored safely in confidential conditions. On all trial-specific documents, other than the signed consent, the participant will be referred to by the trial participant number, not by name.

12.2. Access to Data

Direct access will be granted to authorised representatives from the Sponsor, host institution and the regulatory authorities to permit trial-related monitoring, audits and inspections.

12.3. Data Recording and Record Keeping

All trial data will be entered on to the statistical analysis programme SPSS. The participants will be identified by a unique trial specific number in databases. The name and any other identifying detail will not be included in any trial data electronic file. Source data will be stored in a locked cabinet in a locked room for ten years post publication of the trial results.

13. QUALITY ASSURANCE PROCEDURES

The trial will be conducted in accordance with the current approved protocol, GCP, and standard operating procedures. Data will be evaluated for compliance with the protocol and accuracy in relation to source documents. All electronic data entry is double checked against the source documents. A DMEC and TSC will meet at least annually during the trial.

14. ETHICAL AND REGULATORY CONSIDERATIONS

14.1. Declaration of Helsinki

The Investigator will ensure that this trial is conducted in accordance with the principles of the Declaration of Helsinki.

14.2. Guidelines for Good Clinical Practice

The Investigator will ensure that this trial is conducted in accordance with relevant regulations and with Good Clinical Practice.

14.3. Approvals

The protocol, informed consent form, participant information sheet and any proposed advertising material will be submitted to an appropriate NHS Research Ethics Committee (REC) and host institution for written approval.

The Investigator will submit and, where necessary, obtain approval from the above parties for all substantial amendments to the original approved documents.

14.4. Reporting

The CI shall submit, on request, an Annual Progress Report to the REC, host organisation and Sponsor. In addition, an End of Trial notification and final report will be submitted to the REC and Sponsor.

14.5. Participant Confidentiality

The trial staff will ensure that the participants' anonymity is maintained. The participants will be identified only by a participant ID number on all trial documents and any electronic database, with the exception of the CRF, where participant initials may be added. Audio-recordings of the qualitative interviews will be destroyed after transcription. All documents will be stored securely and only accessible by trial staff and authorised personnel. The trial will comply with the Data Protection Act, which requires data to be anonymised as soon as it is practical to do so.

14.6. Expenses and Benefits

For each trial assessment time point (i.e. three times), patients are reimbursed £15 for their time and effort. Reasonable travel expenses for any visits additional to normal care will be reimbursed on production of receipts, or a mileage allowance provided as appropriate.

14.7. Other Ethical Considerations

There is clinical equipoise between the two psychological treatments. Both are expected to have benefits for patients. However it is hypothesised that gains will be greater with the Feeling Safe Programme. Clinical equipoise exists because the Feeling Safe Programme has not been evaluated in a randomised controlled trial and collective professional opinion would be that such an evaluation is needed to determine its efficacy.

The other main ethical issue is the burden of the assessments for the participants. These typically take one to two hours. However we have successfully used this assessment battery before (indeed have used much longer assessments in trials). It is generally a patient group who have limited social contact, who often have few activities during the day, and who appreciate the time spent with our staff. Hence in our clinical trials there is always improvement in the control condition even when that just comprises the additional monitoring. Patients can take breaks and also complete the assessments over several meetings. Nevertheless if a patient does find the assessments too long then the battery can always be shortened to

the primary measure. However our data completion rates are typically very high, as are our follow-up rates, indicating that patients are fully informed about what the trial will involve.

15. FINANCE AND INSURANCE

15.1. Funding

The trial is funded by a NIHR Research Professorship award to the CI.

15.2. Insurance

The University has a specialist insurance policy in place which would operate in the event of any participant suffering harm as a result of their involvement in the research (Newline Underwriting Management Ltd, at Lloyd's of London). NHS indemnity operates in respect of the clinical treatment that is provided.

16. PUBLICATION POLICY

The results of the trial will be published in a journal. All investigators would be expected to be co-authors.

17. REFERENCES

Bastien, C. H., Vallieres, A. & Morin, C. M. (2001). Validation of the Insomnia Severity Index as an outcome measure for insomnia research. Sleep Medicine, 2, 297-307.

Beck, A.T., Steer, R.A., & Brown, G.K. (1996). Manual for the Beck Depression Inventory-II. San Antonio, TX: Psychological Corporation.

Beecham, J. & Knapp, M. (1992). Costing psychiatric interventions. In Measuring Mental Health Needs (Eds G Thornicroft, CR Brewin, JK Wing) pp 163-184.Gaskell.

Burns, D.D., & Nolen-Hoeksema, S. (1992). Therapeutic empathy and recovery from depression in cognitive-behavioural therapy: a structural equation model. Journal of Consulting and Clinical Psychology, 59, 305-311.

Castle, D.J., Phelan, M., Wessely, S., & Murray, R.M. (1994). Which patients with non-affective functional psychosis are not admitted at first psychiatric contact? British Journal of Psychiatry, 165, 101-106.

Coid, J., Ullrich, S., Kallis, C., Keers, R., Barker, D., Cowden, F., & Stamps, R. (2013). The relationship between delusions and violence. JAMA Psychiatry, 70, 465-471.

Devilly, G. & Borkovec, T. (2000). Psychometric properties of the credibility/expectancy questionnaire. Journal of Behavior Therapy and Experimental Psychiatry, 31, 73-86.

Dowrick, C., Dunn, G., Ayuso-Mateos, J., Dalgard, O., Page, H., Lehtinen, V., Casey, P., Wilkinson, C., Vazquez-Barquero, J., & Wilkinson, G. (2000). Problem solving treatment and group psychoeducation for depression. BMJ, 321, 1450.

Emsley, R., Dunn, G. & White, I.R. (2010). Modelling mediation and moderation of treatment effects in randomised controlled trials of complex interventions. Statistical Methods in Medical Research, 19, 237-270.

Forbes, D., Alkemade, N., Mitchell, D., Elhai, J., McHugh, T., Bates, G. et al. (2014). Utility of the Dimensions of Anger Reactions-5 (DAR-5) scale as a brief anger measure. *Depression and Anxiety, 31*(2), 166-173.

Fowler, D., Freeman, D., Smith, B., Kuipers, E., Bebbington, P., Bashforth, H. et al. (2006). The Brief Core Schema Scales (BCSS): psychometric properties and assocations with paranoia and grandiosity in non-clinical and psychosis samples. Psychological Medicine, 36, 749-759.

Freeman, D., Dunn, G., Startup, H., Pugh, K., Cordwell, J., Mander, H., Cernis, E., Wingham, G., Shirvell, K., & Kingdon, D. (2015).) Effects of cognitive behaviour therapy for worry on persecutory delusions in patients with psychosis (WIT): a parallel, single-blind, randomised controlled trial with a mediation analysis. Lancet Psychiatry, 2, 305-313.

Freeman, D. & Garety, P.A. (2000). Comments on the content of persecutory delusions: Does the definition need clarification? British Journal of Clinical Psychology, 39, 407-414.

Freeman, D. & Garety, P. (2014). Advances in understanding and treating persecutory delusions: a review. Social Psychiatry and Psychiatric Epidemiology, 49, 1179-1189.

Freeman, D., Garety, P.A., & Kuipers, E. (2001). Persecutory delusions: developing the understanding of belief maintenance and emotional distress. Psychological Medicine, 31, 1293-1306.

Freeman, D., Garety, P., Kuipers, E., Fowler, D. & Bebbington, P. (2002). A cognitive model of persecutory delusions. British Journal of Clinical Psychology, 41, 331-347.

Freeman, D., Pugh, K., Dunn, G., Evans, N., Sheaves, B., Waite, F., Cernis, E., Lister, R., & Fowler, D. (2014). An early Phase II randomized controlled trial testing the effect on persecutory delusions of using CBT to reduce negative cognitions about the self. Schizophrenia Research, 160, 186-192.

Freeman, D., Waite, F., Startup, H., Myers, E., Lister, R., McInerney, J., Harvey, A., Geddes, J., Zaiwalla, Z., Luengo-Fernandez, R., Foster, R., Clifton, L., & Yu, L.-M. (In revision). A randomised controlled pilot trial testing the effects of cognitive behavioural therapy to improve sleep for patients with persistent delusions and hallucinations: The Better Sleep Trial (BEST). Lancet Psychiatry.

Gale, N., Heath, G., Cameron, E., Rashid, S., & Redwood, S. (2013). Using the framework method for the analysis of qualitative data in multi-disciplinary health research. BMC Medical Research Methodology, 13, 117.

Garety, P.A., Freeman, D., Jolley, S., Dunn, G., Bebbington, P.E., Fowler, D.G., Kuipers, E., Dudley, R., 2005. Reasoning, emotions and delusional conviction in psychosis. Journal of Abnormal Psychology, 114, 373-384.

Garety, P., Waller, H., Emsley, R., Jolley, S., Kuipers, E., Bebbington, P., Dunn, G., Fowler, D., Hardy, A., & Freeman, D. (2015). Cognitive mechanisms of change in delusions. Schizophrenia Bulletin, 41, 400-410.

Green, C., Freeman, D., Kuipers, E., Bebbington, P., Fowler, D., Dunn, G., & Garety, P.A. (2008). Measuring ideas of persecution and reference: the Green et al Paranoid Thought Scales (G-PTS). Psychological Medicine, 38, 101-111.

Greenwood, K., Sweeney, A., Williams, S., Gaerty, P., Kuipers, E., Scott, J., & Peters, E. (2010). CHoice of Outcome In Cbt for psychosEs (CHOICE): The development of a new service user–led outcome measure of CBT for psychosis. Schizophrenia Bulletin, 36, 126-135.

Haddock, G., McCarron, J., Tarrier, N. & Faragher, F. B. (1999). Scales to measure dimensions of hallucinations and delusions: the psychotic symptom rating scales (PSYRATS). Psychological Medicine, 29, 879-889.

Hartley, S., Haddock, G. & Barrowclough, C. (2012). Anxiety and depression and their links with delusions and hallucinations in people with a dual diagnosis of psychosis and substance misuse. Behaviour Research and Therapy, 50, 65-71.

Hor, K. & Taylor, M. (2010). Suicide and schizophrenia. Journal of Psychopharmacology, 24, s81-90.

Jolley, S., Garety, P.A., Ellett, L., Kuipers, E., Freeman, D., Bebbington, P.E., Fowler, D.G., & Dunn, G. (2006). A validation of a new measure of activity in psychosis. Schizophrenia Research, 85, 288-295.

Kay, S. R. (1991). Positive and Negative Syndromes in Schizophrenia. New York: Brunner.

Leucht, S., Cipriani, A., Spineli, L., Mavridis, D., Örey, D., Richter, F., Samara, M., Barbui, C., Engel, R., Geddes, J., Kissling, W., Stapf, M., Lässig, B., Salanti, G., & Davis, J. (2013). Comparative efficacy and tolerability of 15 antipsychotic drugs in schizophrenia: a multiple-treatments meta-analysis. The Lancet, 382, 951-962.

Li, J.-Z., Guo, Z.-H., Wang, N., Xu, Y.-Z., Qu, Y., Wang, Z.-Q., Sun, J., Yan, L.-Q., Ng, R., Turkington, D. & Kingdon, D. (2015). Cognitive-behavioural therapy for patients with schizophrenia. Psychological Medicine, 45, 1893-1905.

Marsden, J., Gossop, G., Stewart, D., Best, D., Farrell, M., Lehmann, P., Edwards, C., & Strang, J. (1998). The Maudsley Addition Profile (MAP). Addiction, 93, 1857-1867.

Mendelson, B. K., Mendelson, M. J., & White, D. R. (2001). Body-esteem scale for adolescents and adults. Journal of personality assessment, 76 (1), 90-106.

Meyer, T. J., Miller, M. L., Metzger, R. L. & Borkovec, T. D. (1990). Development and validation of the Penn State Worry Questionnaire. Behaviour Research and Therapy, 1990, 28, 487-495.

Morgan, J. F., Reid, F., & Lacey, J. H. (1999) The SCOFF questionnaire: assessment of a new screening tool for eating disorders. British Medical Journal, 319, 1467–1468.

Myers, E., Startup, H. & Freeman, D. (2011). Cognitive behavioural treatment of insomnia in patients with persecutory delusions. Journal of Behavior Therapy and Experimental Psychiatry, 42, 330–336.

Posner, K., Brown, G., Stanley, B., Brent, D., Yershova, K., Oquendo, M., Currier, G., Melvin, G., Greenhill, L., Shen, S., & Mann, J. (2011). The Columbia-Suicide Severity Rating Scale. American Journal of Psychiatry, 168, 1266-1277.

Potter, C., Kelly, L., Hunter, C.., Gibbons, E., Jenkinson, C., Fitzpatrick, R., & Peters, M. (2015, November). *Refining items in the Long Term Conditions Questionnaire (LTCQ): Cognitive Interviews, Stakeholder Feedback and Translatability Assessment.* Poster session presented at the International Society for Quality of life Research, 22nd Annual conference, Vancouver.

Ronald A, Sieradzka D, Cardno A. G., Haworth C. M. A, McGuire P, Freeman D. (2014). Characterization of psychotic experiences in adolescence using the Specific Psychotic Experiences Questionnaire (SPEQ): Findings from a study of 5000 16-year-old twins. *Schizophrenia Bulletin*, 40, 868-77.

Sensky, T., Turkington, D., Kingdon, D., Scott, J., Scott, J.L., Siddle, R., O'Carroll, M., & Barnes, T.R.E. (2000). A randomized controlled trial of cognitive-behavioural therapy for persistent symptoms in schizophrenia resistant to medication. Archives of General Psychiatry, 57, 165-172

Smith, J. A., Flowers, P., & Larkin, M. (2009). Interpretative Phenomenological Analysis: Theory, Method and Research. London: Sage.

Tennant, R., Hiller, L., Fishwick, R., Platt, S., Joseph, S., Weich, S. et al. (2007). The Warwick-Edinburgh Mental Well-being Scale (WEMWBS): development and UK validation. Health and Quality of Life Outcomes, 5, 63-80.

van der Gaag, M., Valmaggia, L., & Smit, F. (2014). The effects of individually tailored formulation-based cognitive behavioural therapy in auditory hallucinations and delusions: a meta-analysis. Schizophrenia Research. http://dx.doi.org/10.1016/j.schres.2014.03.016

Vorontsova, N., Garety, P. & Freeman, D. (2013). Cognitive factors maintaining persecutory delusions in psychosis: the contribution of depression. Journal of Abnormal Psychology, 122, 1121-1131.

Zavos, H.M.S., Freeman, D., Haworth, C.M.A., McGuire, P., Plomin, R., Cardno, A.G., Ronald, A. (2014). Consistent etiology of severe, frequent psychotic experiences and milder, less frequent manifestations: A twin study of specific psychotic experiences in adolescence. JAMA Psychiatry, 71, 1049-1057.

Waller H, Emsley R, Freeman D, Bebbington, P, Dunn G, Fowler D, Hardy A, Kuipers E and Garety P (2015) Thinking Well: A randomised controlled feasibility study of a new CBT therapy targeting reasoning biases in people with distressing persecutory delusional beliefs J Behav Ther Exp Psy 2015 48, 82-89 doi:10.1016/j.jbtep.2015.02.007

Wechsler, D. (1997). Wechsler Adult Intelligence Scale-III/Wechsler Memory Scale. Third Edition Technical Manual. San Antonio, TX: The Psychological Corporation.

18. APPENDIX A: SCHEDULE OF PROCEDURES

Procedures	Visits			
	Screening	Baseline	6 months	12 months
Eligibility assessment	X	X		
Informed consent	X	X		
Demographics		X		
Randomisation		X		
Delusion assessments (PSYRATS, GPTS, belief flexibility)	X	X	X	X
Service user measure (CHOICE)		X	X	X
Quality of Life (EQ-5D; WEMWBS)		X	X	X
Activity (Step count, time-budget)		X	X	X
Psychiatric symptoms (SPEQ, BDI-II, LTCQ; DAR-5)		X	X	X
Moderators (working memory, illicit drug use)		X		
Mediators (PSWQ; BCSS; ISI; JTC; SBQ)		X	X	X
Service receipt (CSRI)		X	X	X
Treatment credibility		X		
Experience of therapy (qualitative interview)				X
Adverse event assessments				X
Onset of delusion (Checklist)		X		
Body image (BEESA; SCOFF; BMI)		X		

19. APPENDIX B: AMENDMENT HISTORY

Amendment No.	Protocol Version No.	Date issued	Author(s) of changes	Details of Changes made
Amendment 1	2.0	8th January 2016	DF, FW	• Secondary outcome measures (pages 5, 10, 14, 15): The PANSS has been removed and the replacement measures added; measure of actigraphy will be replaced with a step count.<br><br>• Additional measures at the baseline assessment (page 15): The measures of belief onset and body image have been added.<br><br>• References (pages 21, 23, 24): All references for the replacement/additional measures have been added<br><br>• Schedule of procedures (page 25): The schedule of procedures has been updated to reflect the change in secondary outcome measures and additional measures at baseline.

Protocol amendments must be submitted to the Sponsor for approval prior to submission to the REC committee or MHRA.

19.1. The Feeling Safe Study: Psychological support for fears about other people

STATISTICAL ANALYSIS PLAN

Version: 1.0

Authors: Prof Richard Emsley, Dr Felicity Waite, Prof Daniel Freeman
Date: 19/06/2020

Protocol version: This SAP has been written based on Protocol V4.0 dated 26th October 2017 and the published trial protocol (Freeman et al, 2016: https://trialsjournal.biomedcentral.com/articles/10.1186/s13063-016-1245-0)

Trial registration: ISRCTN 18705064, registration date: 11/11/2015 http://www.isrctn.com/ISRCTN18705064

19.2. Version history:

Version:	Date:	Changes:
1.0	19/05/2020	First draft of SAP

Principal investigator: Prof Daniel

Freeman Department of Psychiatry

University of Oxford
Felicity.waite@psych.ox.ac.uk

Trial coordinator: Dr Felicity Waite.

Department of Psychiatry
University of Oxford
Felicity.waite@psych.ox.ac.uk

Trial statistician: Prof Richard Emsley

Department of Biostatistics and Health Informatics Institute of Psychiatry, Psychology and Neuroscience Kings College London
Richard.Emsley@kcl.ac.uk

19.3. Chief Investigator: Professor Daniel Freeman

Signature Date .26/06/2020.

19.4. Trial Statistician: Professor Richard Emsley

Signature . Date 23/06/2020

19.5. Trial Steering Group Chair: Professor David Fowler

Signature Date 24/06/2020

19.6. Data Monitoring and Ethics Committee Chair: Professor Paul Bebbington

Signature.. .... Date ..24/6/2020................................

19.7. Data Monitoring and Ethics Committee statistician: Dr Victoria Vickerstaff

Signature..... .... Date ..25/06/2020.................................

1. Objectives of the Statistical Analysis Plan (SAP)

This document details the presentation and analysis strategy for the primary papers reporting results from the Feeling Safe trial. It is intended that the results reported in these papers will follow the strategy set out herein; subsequent papers of a more exploratory nature will not be bound by this analysis plan but will be expected to follow the broad principles laid down for the primary paper(s). The principles are not intended to curtail exploratory analysis or to prohibit sensible statistical and reporting practices but they are intended to establish the strategy that will be followed as closely as possible in analysing and reporting the trial.

Health economic outcomes are addressed briefly however this plan does not include a health economic analysis.

This SAP has been prepared in accordance with the King's Clinical Trial Units Standard Operating Procedure ST-02 (Statistical Analysis Plan). It follows the Guidelines for the Content of Statistical Analysis Plans in Clinical Trials (Gamble et al, 2017).

2. Trial Summary

2.1. Summary

The study is a randomised controlled trial for 150 patients who have persecutory delusions despite previous treatment i.e. the group most at need. A drop-out rate of 15% was allowed for in the power calculation. Patients will be randomised to The Feeling Safe Programme or befriending (both provided over six months). Other treatment, including medication prescription will continue as usual. Assessments, by a rater blind to allocation, will be conducted at 0, 6 (post treatment), and 12 months. All main analyses will be intention-to-treat. A health economic evaluation is included. A small number of qualitative interviews will also be carried out with patients, family members, and NHS staff to determine views of the intervention and implementation.

The design is a parallel group randomised controlled trial with single blind assessment to test whether the new psychological treatment will reduce persecutory delusions more effectively than befriending (an attention control condition). Standard care will be measured but remain as usual in both groups. Assessments will be carried out at 0, 6 (post treatment) and 12 months.

2.2. Primary Objective

**Primary Objective**<br>To test the following hypotheses:<br>1. The Feeling Safe Programme will lead to lower levels of persecutory delusions compared to befriending.	The primary outcome measure will be conviction in the persecutory delusion (using a 0–100% scale), assessed within the Psychotic Symptoms Rating Scale - Delusions (Haddock et al., 1999). We will test dimensional change in conviction levels and rates of recovery in the delusion (defined as conviction falling below 50%). .	0, 6mths, 12mths<br>(Primary endpoint: 6mths).

2.3. Trial Durations and treatment

The trial will run from February 2016 with final assessments due for completion in June 2020.

Participation in the trial is for a duration of 12 months. This includes 20 sessions of psychological therapy (The Feeling Safe Programme or a befriending control condition) provided in the first 6 months of participation.

2.4. Eligibility

Trial participants: Participants with persistent persecutory delusions in the context of non-affective psychosis.

19.8. Inclusion Criteria

- Participant is willing and able to give informed consent for participation in the trial.
- Male or Female, aged 16 years or above.
- Persistent (at least 3 months) persecutory delusion (as defined by Freeman & Garety, 2000), held with at least 60% conviction
- Primary diagnosis of schizophrenia-spectrum psychosis (non-affective psychosis).

19.9. Exclusion Criteria

The participant may not enter the trial if any of the following apply:

- Current receipt of another psychological therapy.
- Insufficient comprehension of English.
- Primary diagnosis of alcohol, drug, or personality disorder.
- In forensic settings.
- Organic syndrome.
- Learning disability

2.5. Randomisation

Randomisation using a permuted blocks algorithm, with randomly varying block size, will be stratified by therapist. Therapists will provide both interventions in order to reduce the confounding of therapist effects and increase statistical power.

2.6. Sample size

For a recovery rate in delusions of 50 % in the Feeling Safe Programme, compared to 20 % with Befriending, a study will have over 90 % power with 60 patients in each arm. The trial will, however, gain greater power by also examining change in delusion dimensional scores. If the standardised effect of the new intervention compared to befriending were smaller than 10 percentage points on the conviction scale (0 to 100 %) (d = 0.5), then we would not consider further development of the intervention to be worth pursuing. If the true effect size were this ten-point difference (SD = 20), then a two-sample t-test with a two-sided significance level of 0.05 would have 80 % power to detect a statistically significant effect with outcome data available for 64 participants per randomised arm. We aim to recruit 75 per arm. This conservatively allows for a drop-out of 15 %. Allowing for stratum membership and baseline levels of the measures in a more refined analysis of covariance will increase both statistical power and precision.

3. Objectives and outcome measures

Objectives	Outcome Measures	Timepoint(s) of evaluation of this outcome measure (if applicable)
**Primary Objective**<br>To test the following hypotheses:<br>1. The Feeling Safe Programme will lead to lower levels of persecutory delusions compared to befriending.	The primary outcome measure will be conviction in the persecutory delusion (using a 0–100% scale), assessed within the Psychotic Symptoms Rating Scale - Delusions (Haddock et al, 1999). We will test dimensional change in conviction levels and rates of recovery in the delusion (defined as conviction falling below 50%).	0, 6mths, 12mths<br>(Primary endpoint: 6mths).
**Secondary Objectives**<br>To test the following hypotheses:<br>1. The Feeling Safe Programme will lead to improved well-being, patient satisfaction, and activity levels compared to befriending.<br>2. The Feeling Safe Programme will lead to lower levels of paranoia, total delusion severity, suicidal ideation, and psychiatric symptoms.<br>3. The benefits will persist at follow-up.	1. Psychological well-being will be assessed by the Warwick-Edinburgh Mental Well-being Scale (Tennant et al, 2007), health status by the EQ-5D-5 L, and quality of life by the Long Term Conditions Questionnaire (LTCQ). Patient satisfaction will be assessed using the CHOICE, a service user-led outcome measure (Greenwood et al, 2010). Activity levels will be assessed using actigraphy (step count) and a time-budget measure (Jolley et al, 2006).<br>2. We will also include measures of overall paranoia (R-GPTS; Freeman et al, 2019), overall delusion severity (PSYRATS Delusions total), depression (BDI-II), suicidal ideation (Columbia-suicide Severity Rating Scale; Posner et al, 2011), anhedonia (TEPS), hallucinations (SPEQ-H voices items), and anger (DAR-5).	0, 6mths, 12mths (primary endpoint: 6mths).
**Tertiary Objectives**<br>To test the following hypotheses:<br>1. Changes in emotional and reasoning processes will mediate the change in delusions.<br>2. Working memory and illicit drug use will moderate treatment effects.	1. For mediation we will include: Safety beliefs (0-100% scale), vulnerability belief (0-100% scale), Penn State Worry Questionnaire (Meyer et al, 1990); Brief Core Schema Scales (Fowler et al, 2006); Insomnia Severity Index (Bastien et al, 2001); Safety Behaviours Questionnaire (Freeman et al, 2001), jumping to conclusions (Garety et al, 2005), possibility of being mistaken (belief flexibility), and anomalous experiences (SPEQ).<br>2. We will include as moderators: working memory (Wechsler, 2007) illicit drug use	0, 6mths, 12mths

3. The Feeling Safe Programme will be cost-effective.	(Marsden et al, 1998), anger (DAR-5), and presence of voices.<br><br>3. We will record all service use, and other relevant health economic data, using an adapted version of the Client Service Receipt Inventory (CSRI; Beecham & Knapp, 1992). Quality of life will be assessed with the EQ-5D-5L (see http://www.euroqol.org/)	

Scoring rules for the outcomes are provided in Appendix 2.

4. General analysis principles

We will report data in line with the Consolidated Standards of Reporting Trials (CONSORT) 2018 Statement for Social and Psychological Interventions (Grant et al, 2018, Trials) showing attrition rates and loss to follow-up (see CONSORT diagram, appendix 1).

Analyses will be carried out using the intention to treat principle: participants analysed in the group they are randomised to, and available data from all participants is included, including those who do not complete therapy. Every effort will be made to follow up all participants in both arms for research assessments.

This statistical analysis plan will be agreed with the Trial Steering Committee and Data Monitoring and Ethics Committee before any inspection of post-randomisation data by the research team.

No interim analysis is planned. All analysis will take place following the end of data collection after the last patient last visit. Significance level (type 1 error) will be 0.05, and 95% confidence intervals will be reported.

Analysis will be conducted in Stata version 16.0 or later.

5. Data summary and reporting

Descriptive statistics within each randomised group will be presented for baseline values. These will include counts and percentages for binary and categorical variables, and means and standard deviations, or medians with lower and upper quartiles, for continuous variables, along with counts of missing values. There will be no tests of statistical significance or confidence intervals for differences between randomised groups on any baseline variable.

Descriptive statistics will be used to summarize withdrawal from intervention and/or from follow-up and completeness of therapy, and timing of withdrawal/loss to follow-up.

Outcomes at 6 and 12 months will be presented separately for each group and summarised using means and standard deviations, along with counts of missing values.

The number of serious adverse events and adverse events will be presented as the number of events and number of individuals with events. These will be provided separately for each randomised group.

6. Statistical methods for inferential analysis

6.1. Analysis of primary and secondary outcomes

The primary outcomes will be analysed using a logistic mixed-effect model with outcome measurement (at the two follow-up time points) as the dependant variable. The model will include fixed effects for timepoint, treatment, timepoint by treatment interactions and therapist. Since an inclusion criteria required each participant to have conviction >60% at screening, all participants have the same baseline value (=1, for presence of conviction) and so there is no requirement for additional baseline adjustment.

For the primary continuous outcome and all secondary outcomes, these will be analysed using linear mixed-effect models with outcome measurement (at the two follow-up time points) as the dependant variable. The model will include fixed effects for timepoint, treatment, timepoint by treatment interactions, the baseline measure of the outcome and therapist, assuming a linear relationship between baseline and outcome.

Observations will be clustered by participant with an unstructured correlation matrix for the residuals. The model will be fitted using restricted maximum likelihood estimation.

For each outcome and timepoint we will report the treatment effect estimate as the adjusted mean difference between groups, its standard error, 95% confidence intervals and p-value.

In addition, we will report estimates for Cohen's D effect sizes as the adjusted mean difference of the outcome divided by the sample standard deviation of the outcome at baseline. Confidence intervals for Cohen's D will be calculated by dividing the confidence limits by the sample standard deviation of the outcome at baseline. These will be displayed in a Forest Plot with the primary outcome at the top, followed by secondary outcomes, with a separate plot for each time point.

6.2. Missing data

Imputation and pro-rating

Missing data on individual measures will be pro-rated on a subscale level if more than 90% of the items of a subscale are completed; otherwise the measure will be considered as missing. Missing values in baseline covariates will be handled using mean imputation – the missing value will be imputed with the mean of the covariate for all participants in the trial (White et al, 2011, BMJ).

Assumptions for primary analysis

The primary analysis assumes data are missing at random, conditional on the observed values of the outcome at baseline, and follow up, and other covariates in the model.

Sensitivity analysis

We will conduct a sensitivity analysis for the primary outcome to assess whether different assumptions about missing data lead to different results. The sensitivity analysis will be conducted using the same model as is used in the primary analysis with the addition of baseline variables found to be predictive of missingness. Baseline variables will be considered predictive of missingness if $p < 0.05$ in a univariate logistic regression model, with attending the visit as the outcome and the baseline variable of interest as the only predictor. This sensitivity analysis will assume data is missing at random conditional on the variables in the primary analysis model and variables that are found to be predictive of missingness.

6.3. Mediation and moderation analysis

The mediation analysis will investigate putative mediational factors using modern causal inference methods. This involves using parametric regression models to test for mediation of the Feeling Safe Intervention on outcome through the putative mediators. Analyses will adjust for baseline measures of the mediator, outcomes, and possible measured confounders. We will include repeated measurement of mediators and outcomes to account for classical measurement error and baseline confounding, and where feasible, use instrumental variable methods (baseline covariate by randomization interactions as potential instruments) to investigate the sensitivity of the estimates to these problems and that of unmeasured confounding.

Moderators will be assessed separately by repeating the primary analysis models and including interaction terms between the randomised intervention and each moderator. The coefficient of the interaction term is a measure of whether the treatment effect differs between levels of the moderator. We will report the difference in treatment effect between levels of the moderator variable.

6.4. Therapy factors

For each group, we will report the number of sessions, total treatment time, modules completed, sessions outside, and credibility score separately. As an exploratory analysis, we will investigate the role of these therapy factors as post-randomisation effect modifiers of any treatment effects.

7. Changes from trial protocol

The published protocol (Freeman et al, 2016) contains the preliminary statistical analysis plan, included a statement that "a full statistical analysis plan will be written by the trial statisticians (RE, GD) prior to any analysis being undertaken". This document details the full SAP and deviates from that published in the protocol in the following ways:

- The relevant CONSORT statement for this trial is now the updated 2018 statement for social and psychological interventions, not the 2010 statement as previously stated.

- Analysis will be conducted in Stata version 16.1 or later, not version 14 as previously stated.

- We will use linear or logistic mixed models to estimate the treatment effects at 6 and 12 months in one model with restricted maximum likelihood estimation, rather than fitting and estimating separate linear or logistic models at each time point. All other details, including covariates for adjustment, are as previously stated and the interpretation of the coefficients from the model as estimates of the treatment effect is unchanged. The advantage of the mixed model approach is to allow missingness of outcome data at 12 months to be conditional on observed data at 6 months under a missing at random missingness assumption.

- The scoring for the Green Paranoid Thought Scale will be the revised Freeman et al 2019 scoring of the GTPS items.

19.10. Appendix 1: Example analysis code

Data will be in long format with two rows for each participant, one for 6 month time point and one row for the 12 month timepoint.

Variable names

- pid: participant id
- treat: Arm of the trial participant is randomised to
- timepoint: follow-up timepoint
- baseline: baseline measure of the outcome
- therapist: Stratification factor
- outcome: outcome measure

Analysis code

20. *Model for binary outcomes analysed using mixed effect model:

```
melogit outcome i.treat##i.timepoint therapist || pid:, reml

melogit, or


**Follow-up

melogit outcome i.treat##ib12.timepoint therapist || pid:, reml

melogit, or
```

21. *Model for continuous outcomes analysed using mixed effect model:

```
mixed outcome i.treat##i.timepoint baseline therapist || pid:, /// res(unstructured, t(timepoint))
noconstant reml

margins treat, at(timepoint==6) pwcompare(effects)


**Follow-up

margins treat, at(timepoint==12) pwcompare(effects)

//Should be the same as main effect of treat in:

// mixed outcome i.treat##ib12.timepoint baseline therapist || pid:, /// res(unstructured,
t(timepoint)) noconstant reml
```

21.1. Appendix 2: Deriving outcomes

Scoring rules for outcomes

Outcome acronym	Number of questions	Scoring	Min-Max possible values	Scores for better outcomes
Conviction_PrimaryOutcome	1	Scoring method 1: 0-100 dimensional.<br><br>Scoring method 2: categorical 0-49 = 1 (recovery), 50-100 = 0 (persistent).	0-100.<br><br>0-1.	Scoring method 1: Lower scores better.<br><br>Scoring method 2: higher scores better.
PSYRATS_total	6	Likert scale: 0-4	0-24	Lower scores better.
R-GPTS_PtA_Total	8	Likert scale: 0-4	0-32	Lower scores better.
R-GPTS_PtB_Total	10	Likert scale: 0-4	0-40	Lower scores better.
BDI_Total	21	Likert scale: 0-3	0-63	Lower scores better.
Anhedonia_Total	10	Likert scale: 1-6	10-60	Higher scores better.
CHOICE_Total	12	Likert scale: 1-10	12-120	Higher sores better.
Time_Budget_Total	1	Total score *(level of activity rated between 0-3 for 28 datapoints: 4 timepoints per day for 7 days)*	0-112	Higher sores better.
WEMWBS_Total	14	Likert scale: 1-5	14-70	Higher sores better.
LTC_Total	20	Likert scale: 0-4	0-80	Higher sores better.
EQ5-D	2	Scoring method 1: Crosswalk index value (0-1 dimensional) *calculated from the score of 5 items each scored 1-5 using EuroQol index data.*<br><br>Scoring method 2: 1 item dimensional 0-100	0-1<br><br>0-100	Higher sores better.<br><br>Higher sores better.
CSSRS_Ideation	1	Ideation item only	0-5	Lower scores better.
Mean_Daily_stepcount	1	Mean daily step count.	0 ≤	Higher sores better.
Hallucinations_Total	3	Likert scale: 0-5	0-15	Lower scores better.
Anger_Total	5	Likert scale: 1-5	5-25	Lower scores better.

21.2. Appendix 3: Dummy tables for primary publication

Table 1: Baseline Characteristics – Demographics

Baseline characteristics		Feeling Safe	Befriending	Sample
Age	Mean(SD)			
Sex N(%)	Female			
	Male			
Ethnicity N(%)	White			
	Black Caribbean			
	Black African			
	Black other			
	Indian			
	Pakistani			
	Chinese			
	Other			
Employment (N%)	Unemployed			
	Employed FT			
	Employed PT			
	Self Employed			
	Retired			
	Student			
	Housewife / Husband			
No. hours working/week	Mean(SD)			
No. hours volunteering/week	Mean(SD)			
Marital Status (N%)	Single			
	Cohabiting			
	Married or Civil Partnership			
	Divorced			
	Widowed			
Living situation	Living along (+/- children)			
	Living with spouse (+/- children)			
	Living together as a couple			
	Living with parents			
	Living with other relatives			
	Living with others			
Diagnosis (n)	Schizophrenia			
	Schizo-affective disorder			
	Delusional disorder			

	Psychosis NOS			
Medication prescribed	Antipsychotic			
	Antidepressant			
	Anxiolytic			
	Mood stabiliser			
	Hypnotic			
	Stimulant			
	Total number of psychotropics			
	Antipsychotic_CPZequiv			

Table 2: Primary and secondary outcomes

	No. (% of group)		
Outcome	Feeling Safe	Befriending	OR (SE); p-value (95% CI)
Conviction			
Month 6 – Yes			
No			
Month12 - Yes			
No			

	Unadjusted, Mean (SD)			
Outcome	Feeling Safe<br>*n*= XX	Befriending<br>*n*= XX	Adjusted Difference (SE); p-value (95% CI)	Cohen *d*<br>*(95% CI)*
Outcome				
Baseline			-	-
6 months				
12 months				

Table 3: Description of Adverse Events (AE)

Description			Feeling Safe	Befriending
			Total number of events (people)	Total number of events (people)
Death				
Suicide attempts				
Violent incidents (needing police involvement)				
Formal complaints about therapy				
Hospital admission	Physical health			
	Psychiatric	Under section		
		Informal		
Attendance at A&E				
Other: safeguarding concerns				
Other: complaint regarding trial team – suspected breach of confidentiality				
Total				

Appendix 4: CONSORT diagram

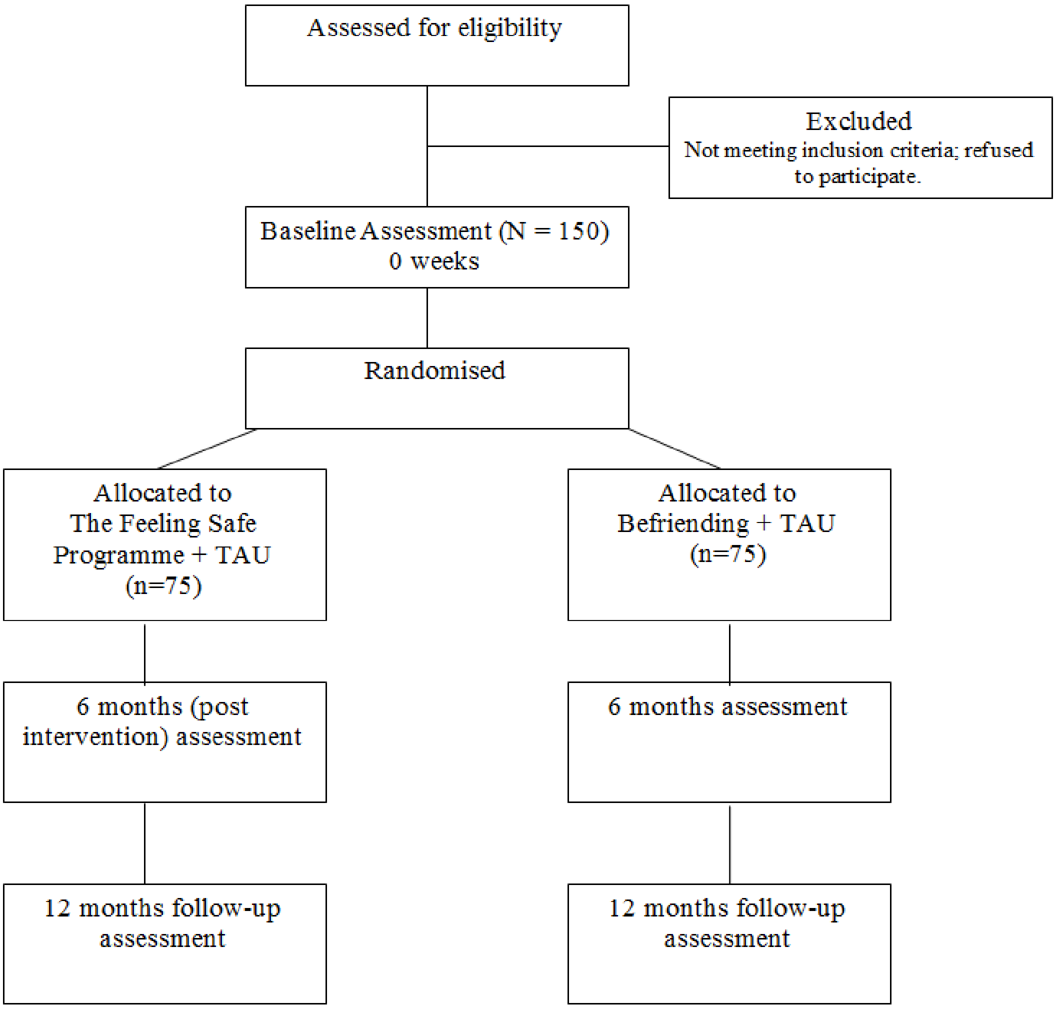

Table 1: Baseline characteristics

Baseline characteristics		Befriending n=66	Feeling Safe n=64	Sample n=130
Age, mean(SD)		41.3 (12.0)	41.9 (12.3)	41.6 (12.1)
Sex, n(%)	Female	22 (33.3%)	30 (46.9%)	52 (40.0%)
	Male	44 (66.7%)	34 (53.1%)	78 (60.0%)
Ethnicity, n(%)	White	58 (87.9%)	52 (81.3%)	110 (84.%)
	Black Caribbean	5 (7.6%)	3 (4.7%)	8 (6.2%)
	Black African	0	2 (3.1%)	2 (1.5%)
	Black other	0	1 (1.6%)	1 (0.8%)
	Indian	1 (1.5%)	2 (3.1%)	3 (2.3%)
	Pakistani	1 (1.5%)	2 (3.1%)	3 (2.3%)
	Chinese	0	1 (1.6%)	1 (0.8%)
	Other	1 (1.5%)	1 (1.6%)	2 (1.5%)
Employment, n(%)	Unemployed	51 (77.3%)	51 (79.7%)	102 (78.5%)
	Employed FT	5 (7.6%)	1 (1.6%)	6 (4.6%)
	Employed PT	5 (7.6%)	4 (6.3%)	9 (6.9%)
	Self Employed	0	1 (1.6%)	1 (0.8%)
	Retired	2 (3.0%)	2 (3.1%)	4 (3.1%)
	Student	2 (3.0%)	2 (3.1%)	4 (3.1%)
	Housewife / Husband	1 (1.5%)	3 (4.7%)	4 (3.1%)
No. hours/week, mean(SD)	Working	4.1 (10.9)	2.1 (7.5)	3.2 (9.4)
	Volunteering	0.5 (1.4)	0.4 (1.5)	0.4 (1.4)
Marital status, n(%)	Single	49 (74.6%)	43 (67.2%)	92 (70.8%)
	Cohabiting	2 (3.0%)	0	2 (1.5%)
	Married/Civil Partnership	8 (12.1%)	18 (28.1%)	26 (20.0%)
	Divorced	7 (10.6%)	3 (4.7%)	10 (7.7%)
Living situation, n(%)	Living alone (+/- children)	29 (43.9%)	23 (35.9%)	52 (40.0%)
	Living with spouse	10 (15.2%)	14 (21.9%)	24 (18.5%)
	Living together as a couple	2 (3.0%)	2 (3.1%)	4 (3.1%)
	Living with parents	17 (25.8%)	10 (15.6%)	27 (20.8%)
	Living with other relatives	0	4 (6.3%)	4 (3.1%)
	Living with others	8 (12.1%)	11 (17.2%)	19 (14.6%)
Diagnosis, n(%)	Schizophrenia	43 (65.2%)	36 (56.3%)	79 (60.8%)
	Schizo-affective disorder	9 (13.6%)	13 (20.3%)	22 (16.9%)
	Delusional disorder	2 (3.0%)	2 (3.1%)	4 (3.1%)
	Psychosis NOS	12 (18.2%)	13 (20.3%)	25 (19.2%)

Table 2: Baseline Medications

**Prescribed medications - Baseline**	**Befriending n=66**	**Feeling Safe n=64**	**Total n=130**
Antipsychotic, n(%)	64 (97%)	61 (95.3%)	125 (96.2%)
Antidepressant, n(%)	47 (71.2%)	32 (50.0%)	79 (60.8%)
Anxiolytic, n(%)	4 (6.1%)	7 (10.9%)	11 (8.5%)
Mood stabiliser, n(%)	10 (15.2%)	8 (12.5%)	18 (13.8%)
Hypnotic, n(%)	7 (10.6%)	5 (7.8%)	12 (9.2%)
Total number of psychotropics, mean(SD)	2.6 (1.2)	2.3 (1.3)	2.4 (1.2)
Antipsychotic CPZ equivalent dose (mg/day), mean(SD)*	514.1 (412.9)	449.9 (392.7)	482.7 (402.8)

**Data missing for 5 patients (Befriending n=2, Feeling Safe n=3)*

Table 3: Medications at 6 months

**Prescribed medications – 6 months**	**Befriending n=64**	**Feeling Safe n=62**	**Total n=126**
Antipsychotic, n(%)	60 (93.8%)	60 (96.8%)	120 (95.2%)
Antidepressant, n(%)	42 (65.6%)	32 (51.6%)	74 (58.7%)
Anxiolytic, n(%)	4 (6.3%)	9 (14.5%)	13 (10.3%)
Mood stabiliser, n(%)	10 (15.6%)	8 (12.9%)	18 (14.3%)
Hypnotic, n(%)	8 (12.5%)	7 (11.3%)	15 (11.9%)
Total number of psychotropics, mean(SD)	2.5 (1.4)	2.3 (1.3)	2.4 (1.3)
Antipsychotic CPZ equivalent dose (mg/day), mean(SD)*	545.3 (408.7)	462.4 (404.1)	503.9 (406.8)

Table 4: Medications at 12 months

**Prescribed medications – 12 months**	**Befriending n=63**	**Feeling Safe n=60**	**Total n=123**
Antipsychotic, n(%)	59 (93.7%)	57 (95.0%)	116 (94.3%)
Antidepressant, n(%)	44 (69.8%)	35 (58.3%)	79 (64.2%)
Anxiolytic, n(%)	3 (4.8%)	7 (11.7%)	10 (8.1%)
Mood stabiliser, n(%)	9 (14.3%)	10 (16.7%)	19 (15.4%)
Hypnotic, n(%)	9 (14.3%)	3 (5.0%)	12 (9.8%)
Total number of psychotropics, mean(SD)	2.5 (1.4)	2.4 (1.3)	2.5 (1.3)
Antipsychotic CPZ equivalent dose (mg/day), mean(SD)*	547.0 (403.3)	464.1 (399.6)	506.2 (401.9)

Table 5: Adverse events

Description	Befriending Total number of events (people)	Feeling Safe Total number of events (people)
**Adverse events at 6 months**	**35 (14)**	**26 (11)**
Suicide Attempts		
No treatment required	13 (2)	7 (4)
Treatment required	0	0
A&E attendance	3 (3)	3 (1)
During inpatient stay	0	1 (1)
Death	0	0
Life-threatening injury	0	0
Hospital admission		
Physical	4 (3)	3 (3)
Psychiatric	6 (6)	5 (4)
Under section	(3)	(2)
Informal	(2)	(2)
Attendance at A&E		
Physical	4(4)	3 (2)
Psychiatric	2 (2)	3 (3)
Violent incident (police involvement)	0	0
Disability	0	0
Foetal Harm	0	0
Formal complaints about therapy	0	0
Other	3 (2)	1 (1)
Ambulance services at house	0	1 (1)
DSH requiring medical treatment	2 (1)	0
Safeguarding concerns, threat from neighbours	1 (1)	0
**Adverse events at 12 months**	**33 (11)**	**27 (13)**
Suicide Attempts		
No treatment required	2 (2)	4 (3)
Treatment required	1 (1)	0
A&E attendance	1 (1)	4 (1)
During inpatient stay	1 (1)	0
Death	0	0
Life-threatening Injury	0	0
Hospital admission		
Physical	2 (2)	3 (3)
Psychiatric	8 (7)	8 (7)
Under section	(5)	(5)
Informal	(3)	(4)
Attendance at A&E		

Description	Befriending Total number of events (people)	Feeling Safe Total number of events (people)
Physical	2 (2)	5 (5)
Psychiatric	10 (2)	2 (2)
Violent incident (police involvement)	0	0
Disability	0	0
Foetal Harm	0	0
Formal complaints about therapy	0	0
Other	5 (2)	0
DSH requiring medical treatment	4 (1)	0
999 call and ambulance/ paramedics visited residence	1 (1)	0
**TOTAL**	**68 (20)**	**53 (16)**

Table 6: Outcome scoring by timepoint

	Baseline			6 months			12 months		
**Clinical scales**	**Befriending mean (SD)**	**Feeling Safe mean (SD)**	**Overall mean (SD)**	**Befriending mean (SD)**	**Feeling Safe mean (SD)**	**Overall mean (SD)**	**Befriending mean (SD)**	**Feeling Safe mean (SD)**	**Overall mean (SD)**
Conviction <50%, n(%)	0 (0%)	0 (0%)	0 (0%)	22 (34.9%)	32 (50.8%)	54 (42.9%)	22 (34.9%)	27 (46.6%)	49 (40.5%)
Conviction	86.4 (12.6)	87.1 (12.2)	86.8 (12.4)	59.6 (27.1)	49.4 (35.5)	54.5 (31.9)	59.4 (32.8)	50.2 (36.0)	55.0 (24.5)
PSYRATS	18.2 (2.6)	18.5 (2.3)	18.3 (2.5)	14.2 (4.8)	11.6 (5.9)	12.9 (6.0)	13.5 (5.6)	11.6 (6.4)	12.6 (6.0)
PSYRATS 25% reduction, n(%)	-	-	-	25 (37.9%)	38 (59.4%)	63 (48.5%)	27 (40.9%)	32 (50.0%)	59 (48.5%)
PSYRATS 50% reduction, n(%)	-	-	-	9 (13.6%)	21 (32.8%)	30 (23.1%)	10 (15.2%)	21 (32.8%)	31 (23.8%)
RGPTS PtA	17.4 (8.2)	17.3 (7.2)	17.3 (7.7)	12.6 (8.2)	10.2 (7.1)	11.4 (8.1)	13.1 (9.0)	10.2 (7.9)	11.7 (8.2)
RGPTS PtB	27.4 (8.5)	26.7 (8.2)	27.1 (8.4)	17.2 (11.1)	14.3 (11.8)	15.8 (11.5)	17.9 (12.2)	14.2 (12.3)	16.1 (12.4)
RGPTS Total	44.8 (14.6)	43.9 (13.8)	44.4 (14.2)	29.8 (17.3)	24.4 (18.4)	27.2 (18.0)	31.0 (19.7)	24.4 (17.6)	27.8 (18.9)
BDI	31.9 (12.4)	30.2 (11.3)	31.1 (11.9)	21.5 (12.6)	18.8 (12.0)	20.2 (12.3)	23.1 (13.8)	20.3 (13.5)	21.8 (13.7)
Anhedonia	30.1 (11.8)	30.8 (11.0)	30.4 (11.4)	34.1 (12.4)	35.6 (12.0)	34.8 (12.2)	32.5 (12.0)	35.1 (10.3)	33.8 (11.2)
CHOICE	48.5 (18.7)	47.7 (15.0)	48.1 (16.9)	61.7 (21.1)	68.3 (21.4)	64.9 (21.4)	61.4 (23.3)	69.3 (21.3)	65.1 (22.6)
Time Budget	56.3 (14.3)	51.0 (15.0)	53.7 (14.9)	59.8 (15.6)	59.4 (15.3)	59.6 (15.4)	61.1 (16.9)	57.7 (15.6)	59.5 (16.3)
WEMWBS	35.1 (8.8)	34.0 (8.2)	34.5 (8.5)	39.7 (10.8)	43.8 (10.1)	41.7 (10.6)	39.4 (9.6)	41.3 (10.0)	40.3 (10.5)
LTC	35.2 (11.9)	34.5 (8.7)	34.8 (10.5)	41.6 (13.7)	44.9 (13.0)	43.2 (13.4)	41.6 (14.2)	44.3 (11.5)	42.9 (13.0)
Anger	10.7 (4.5)	11.1 (4.8)	10.9 (4.6)	9.6 (4.3)	8.3 (3.4)	9.0 (4.0)	9.6 (5.1)	9.1 (4.5)	9.4 (4.8)
Hallucinations	7.4 (5.9)	7.6 (5.9)	7.5 (5.9)	6.4 (5.5)	6.1 (5.8)	6.2 (5.6)	5.1 (5.2)	7.0 (5.7)	6.0 (5.5)
CSSRS	2.0 (1.6)	1.9 (1.6)	1.9 (1.6)	1.4 (1.6)	1.4 (1.6)	1.4 (1.6)	1.3 (1.4)	1.5 (1.7)	1.4 (1.5)
Fitbit steps	7910.9 (6043.2)	6176.1 (3425.2)	7182.7 (5155.5)	6749.1 (4826.7)	6413.1 (4307.8)	6606.5 (4582.3)	-	-	-
EQ5D Index	0.5 (0.3)	0.5 (0.3)	0.5 (0.3)	0.6 (0.3)	0.6 (0.2)	0.6 (0.2)	0.6 (0.3)	0.6 (0.2)	0.6 (0.3)
EQ5D health today	47.9 (21.0)	50.2 (20.2)	49.0 (20.6)	56.5 (23.3)	58.4 (20.7)	57.4 (22.0)	53.8 (25.2)	60.7 (21.1)	57.0 (23.5)

Table 7: Primary outcome analysis – Conviction (cont.)

	Befriending n=66<br>Unadjusted mean (SD)	Feeling Safe n=64<br>Unadjusted mean (SD)	Adjusted Difference* (SE); p-value (95% CI)	*Cohen d* (95% CI)
**Conviction**				
Baseline	86.4 (12.6)	87.1 (12.2)	-	-
6 months	59.6 (27.1)	49.4 (35.5)	10.690 (4.621); p=0.021 (1.632, 19.747)	0.864 (0.132, 1.596)
12 months	59.4 (32.8)	50.2 (36.0)	8.428 (4.714); p=0.074 (0.811, 17.668)	0.681 (0.066, 1.428)

**Adjusted for baseline score, therapist, and interaction of timepoint with treatment allocation, and including a random effect at the individual level*

Table 8: Primary outcome analysis - Conviction (binary)

	Befriending n=66<br>n (%)	Feeling Safe n=64<br>n (%)	OR (SE); p-value (95% CI)
**Conviction**			
Baseline	0 (0%)	0 (0%)	-
6 months	22 (34.9%)	32 (50.8%)	3.944 (2.956); p=0.067 (0.908-17.134)
12 months	22 (34.9%)	27 (46.6%)	2.364 (1.740); p=0.242 (0.559-10.000)

Table 9: Secondary outcome analysis

	**Befriending n=66**<br>Unadjusted mean (SD)	**Feeling Safe n=64**<br>Unadjusted mean (SD)	**Adjusted Difference* (SE); p-value (95% CI)**	***Cohen d* (95% CI)**
**PSYRATS**				
Baseline	18.2 (2.6)	18.5 (2.3)	-	-
6 months	14.2 (4.8)	11.6 (5.9)	2.944 (0.834); p<0.0001 (1.309, 4.579)	1.195 (0.532, 1.859)
12 months	13.5 (5.6)	11.6 (6.4)	2.135 (0.850); p=0.012 (0.468, 3.802)	0.867 (0.190, 1.544)
**RGPTS Pt A**				
Baseline	17.4 (8.2)	17.3 (7.2)	-	-
6 months	12.6 (8.2)	10.2 (7.1)	2.391 (1.037); p=0.021 (0.358, 4.423)	0.312 (0.047, 0.577)
12 months	13.1 (9.0)	10.2 (7.9)	2.527 (1.052); p=0.016 (0.464, 4.590)	0.330 (0.061, 0.599)
**RGPTS Pt B**				
Baseline	27.4 (8.5)	26.7 (8.2)	-	-
6 months	17.2 (11.1)	14.3 (11.8)	2.857 (1.614); p=0.077 (-0.307, 6.021)	0.342 (-0.037, 0.721)
12 months	17.9 (12.2)	14.2 (12.3)	3.341 (1.642); p=0.042 (0.124, 6.559)	0.400 (0.014, 0.785)
**RGPTS Total**				
Baseline	44.8 (14.6)	43.9 (13.8)	-	-
6 months	29.8 (17.3)	24.4 (18.4)	5.590 (2.428) p=0.021 (0.831, 10.348)	0.395 (0.059, 0.731)
12 months	31.0 (19.7)	24.4 (17.6)	5.921 (2.461) p=0.016 (1.100, 10.746)	0.418 (0.078, 0.759)
**BDI**				
Baseline	31.9 (12.4)	30.2 (11.3)	-	-
6 months	21.5 (12.6)	18.8 (12.0)	2.319 (1.631); p=0.155 (-0.876, 5.515)	0.195 (-0.074, 0.464)
12 months	23.1 (13.8)	20.3 (13.5)	1.667 (1.660); p=0.315 (-1.586, 4.921)	0.141 (-0.133, 0.414)
**Anhedonia**				
Baseline	30.1 (11.8)	30.8 (11.0)	-	-
6 months	34.1 (12.4)	35.6 (12.0)	1.148 (1.202); p=0.340 (-1.208, 3.504)	0.101 (-0.106, 0.308)
12 months	32.5 (12.0)	35.1 (10.3)	1.443 (1.214); p=0.235 (-0.937, 3.823)	0.127 (-0.082, 0.336)

	Befriending n=66	Feeling Safe n=64	Adjusted Difference* (SE); p-value (95% CI)	*Cohen d* (95% CI)
	Unadjusted mean (SD)	Unadjusted mean (SD)		
**CHOICE**				
Baseline	48.5 (18.7)	47.7 (15.0)	-	-
6 months	61.7 (21.1)	68.3 (21.4)	7.917 (3.068); p=0.010 (1.904, 13.930)	0.469 (0.113, 0.825)
12 months	61.4 (23.3)	69.3 (21.3)	7.562 (3.111); p=0.015 (1.46,5 13.660)	0.448 (0.087, 0.809)
**Time Budget**				
Baseline	56.3 (14.3)	51.0 (15.0)	-	-
6 months	59.8 (15.6)	59.4 (15.3)	5.033 (2.099); p=0.016 (0.920, 9.147)	0.338 (0.062, 0.615)
12 months	61.1 (16.9)	57.7 (15.6)	1.987 (2.180); p=0.362 (-2.287, 6.260)	0.134 (-0.154, 0.421)
**WEMWBS**				
Baseline	35.1 (8.8)	34.0 (8.2)	-	-
6 months	39.7 (10.8)	43.8 (10.1)	5.087 (1.431); p<0.0001 (2.282, 7.891)	0.600 (0.269, 0.930)
12 months	39.4 (9.6)	41.3 (10.0)	2.263 (1.459); p=0.121 (-0.596, 5.122)	0.267 (-0.070, 0.604)
**LTC**				
Baseline	35.2 (11.9)	34.5 (8.7)	-	-
6 months	41.6 (13.7)	44.9 (13.0)	3.310 (1.744); 0.058 (-0.108, 6.728)	0.317 (-0.010, 0.643)
12 months	41.6 (14.2)	44.3 (11.5)	2.115 (1.777); p=0.234 (-1.368, 5.598)	0.202 (-0.131, 0.535)
**Anger**				
Baseline	10.7 (4.5)	11.1 (4.8)	-	-
6 months	9.6 (4.3)	8.3 (3.4)	1.450 (0.601) p=0.016 (0.271, 2.628)	0.312 (0.058, 0.565)
12 months	9.6 (5.1)	9.1 (4.5)	0.466 (0.609) p=0.445 (-0.728, 1.660)	0.100 (-0.157, 0.357)
**Hallucinations**				
Baseline	7.4 (5.9)	7.6 (5.9)	-	-
6 months	6.4 (5.5)	6.1 (5.8)	1.072 (0.611) p=0.080 (-0.126, 2.270)	0.183 (-0.022, 0.388)
12 months	5.1 (5.2)	7.0 (5.7)	-1.095 (0.612) p=0.073 (-2.294, 0.104)	-0.187 (-0.392, 0.018)
**CSSRS**				
Baseline	2.0 (1.6)	1.9 (1.6)	-	-
6 months	1.4 (1.6)	1.4 (1.6)	-0.184 (0.222)	-0.117

	Befriending n=66<br>Unadjusted mean (SD)	Feeling Safe n=64<br>Unadjusted mean (SD)	Adjusted Difference* (SE); p-value (95% CI)	*Cohen d* (95% CI)
			p=0.408 (-0.620, 0.252)	(-0.394, 0.160)
12 months	1.3 (1.4)	1.5 (1.7)	-0.331 (0.232)<br>p=0.153 (-0.785, 0.123)	-0.211<br>(-0.499, 0.078)
**Fitbit Steps**				
Baseline	7910.9 (6043.2)	6176.1 (3425.2)	-	-
6 months	6749.1 (4826.7)	6413.1 (4307.8)	482.981 (638.885) p=0.450 (-769.211, 1735.174)	0.094<br>(-0.149, 0.337)
12 months	-	-	-	-
**EQ5D - Index**				
Baseline	0.5 (0.3)	0.5 (0.3)	-	-
6 months	0.6 (0.3)	0.6 (0.2)	0.074 (0.033)<br>p=0.027 (0.008, 0.139)	0.280<br>(0.032, 0.529)
12 months	0.6 (0.3)	0.6 (0.2)	0.047 (0.034)<br>p=0.169 (-0.020, 0.113)	0.177<br>(-0.075, 0.429)
**EQ5D - Health today**				
Baseline	47.9 (21.0)	50.2 (20.2)	-	-
6 months	56.5 (23.3)	58.4 (20.7)	1.586 (3.424)<br>p=0.643 (-5.125, 8.297)	0.077<br>(-0.249, 0.404)
12 months	53.8 (25.2)	60.7 (21.1)	6.803 (3.508)<br>p=0.052 (-0.073, 13.680)	0.331<br>(-0.004, 0.665)

Table 10: PSYRATS Binary analysis

	Befriending n=66<br>n (%)	Feeling Safe n=64<br>n (%)	OR (SE); p-value (95% CI)
**PSYRATS**			
**25% reduction**			
Baseline	-	-	-
6 months	25 (37.9%)	38 (59.4%)	5.393 (3.868)<br>p=0.019 (1.322, 21.997)
12 months	27 (40.9%)	32 (50.0%)	2.034 (1.379)<br>p=0.295 (0.539, 7.678)
**50% reduction**			
Baseline	-	-	-

6 months	9 (13.6%)	21 (32.8%)	7.764 (6.780)<br>p=0.019 (1.402, 42.992)
12 months	10 (15.2%)	21 (32.8%)	6.258 (5.311)<br>p=0.031 (1.186, 33.020)

Table 11: Missing responses for outcomes by timepoint

	Baseline			6 months			12 months		
**Clinical scales**	**BF n=66**	**FS n=64**	**Overall n=130**	**BF n=66**	**FS n=64**	**Overall n=130**	**BF n=66**	**FS n=64**	**Overall n=130**
Conviction <50%	0 (0%)	0 (0%)	0 (0%)	3 (4.5%)	1 (1.6%)	4 (3.1%)	3 (4.5%)	6 (9.4%)	9 (6.9%)
Conviction	0 (0%)	0 (0%)	0 (0%)	3 (4.5%)	1 (1.6%)	4 (3.1%)	3 (4.5%)	6 (9.4%)	9 (6.9%)
PSYRATS	0 (0%)	0 (0%)	0 (0%)	4 (6.1%)	3 (4.7%)	7 (5.4%)	3 (4.5%)	9 (14.1%)	12 (9.2%)
RGPTS PtA	1 (1.5%)	1 (1.6%)	2 (1.5%)	4 (6.1%)	4 (6.3%)	8 (6.2%)	5 (7.6%)	7 (10.9%)	12 (9.2%)
RGPTS PtB	0 (0%)	1 (1.6%)	1 (0.8%)	4 (6.1%)	4 (6.3%)	8 (6.2%)	5 (7.6%)	7 (10.9%)	12 (9.2%)
RGPTS Total	1 (1.5%)	1 (1.6%)	2 (1.5%)	4 (6.1%)	4 (6.3%)	8 (6.2%)	5 (7.6%)	7 (10.9%)	12 (9.2%)
BDI	0 (0%)	0 (0%)	0 (0%)	5 (7.6%)	5 (7.8%)	10 (7.7%)	6 (9.1%)	8 (12.5%)	14 (10.8%)
Anhedonia	1 (1.5%)	0 (0%)	1 (0.8%)	5 (7.6%)	6 (9.4%)	11 (8.5%)	6 (9.1%)	7 (10.9%)	13 (10.0%)
CHOICE	1 (1.5%)	2 (3.1%)	3 (2.3%)	6 (9.1%)	9 (14.1%)	15 (11.5%)	7 (10.6%)	11 (17.2%)	18 (13.8%)
Time Budget	2 (3.0%)	2 (3.1%)	4 (3.1%)	7 (10.6%)	10 (15.6%)	17 (13.1%)	11 (16.7%)	15 (23.4%)	28 (21.5%)
WEMWBS	0 (0%)	0 (0%)	0 (0%)	3 (4.5%)	3 (4.7%)	6 (4.6%)	4 (6.1%)	7 (10.9%)	11 (11.5%)
LTC	2 (3.0%)	3 (4.7%)	5 (3.8%)	7 (10.6%)	9 (14.1%)	16 (12.3%)	7 (10.6%)	12 (18.8%)	30 (23.1%)
Anger	1 (1.5%)	2 (3.1%)	3 (2.3%)	6 (9.1%)	12 (18.8%)	18 (13.8%)	9 (13.6%)	13 (20.3%)	22 (16.9%)
Hallucinations	1 (1.5%)	0 (0%)	1 (0.8%)	5 (7.6%)	5 (7.8%)	10 (7.7%)	4 (6.1%)	6 (9.4%)	10 (7.7%)

CSSRS	1 (1.5%)	2 (3.1%)	3 (2.3%)	8 (12.1%)	10 (15.6%)	18 (13.8%)	11 (16.7%)	16 (25.0%)	27 (20.8%)
Fitbit Steps	19 (28.8%)	30 (23.1%)	49 (37.7%)	28 (42.4%)	36 (54.5%)	64 (49.2%)	-	-	-
EQ5D Index	1 (1.5%)	0 (0%)	1 (0.8%)	4 (6.1%)	8 (12.5%)	12 (9.2%)	5 (7.6%)	10 (15.6%)	15 (11.5%)
EQ5D health today	1 (1.5%)	0 (0%)	1 (0.8%)	4 (6.1%)	6 (9.4%)	10 (7.7%)	5 (7.6%)	10 (15.6%)	15 (11.5%)

Table 12: Secondary outcome analysis (mediators)

	**Befriending n=66** Unadjusted mean (SD)	**Feeling Safe n=64** Unadjusted mean (SD)	**Adjusted Difference* (SE); p-value (95% CI)**	***Cohen d* (95% CI)**
**Safety beliefs**				
Baseline	37.88 (29.64)	37.75 (26.34)	-	-
6 months	50.67 (28.71)	55.62 (29.41)	-6.519 (4.463); p=0.144 (-15.266, 2.227)	-0.233 (- 0.546, 0.080)
12 months	49.03 (29.47)	57.00 (28.58)	-8.094 (4.539); p=0.075 (-16.990, 0.803)	-0.290 (-0.608, 0.029)
**Vulnerability**				
Baseline	75.15 (23.26)	72.28 (24.07)	-	-
6 months	63.7 (26.89)	49.31 (32.24)	12.669 (4.731); p=0.007 (3.400, 21.941)	0.536 (0.144, 0.929)
12 months	56.73 (2733)	47.77 (31.07)	7.202 (4.811); p=0.134 (-2.227, 16.631)	0.305 (-0.094, 0.704)
**PSWQ Total**				
Baseline	63.26 (11.27)	62.58 (10.68)	-	-
6 months	57.35 (12.78)	54.25 (15.51)	3.327 (1.667); p=0.046 (0.060, 6.594)	0.304 (0.005, 0.602)
12 months	58.30 (12.21)	54.65 (11.34)	3.109 (1.680); p=0.064 (-0.184, 6.402)	0.284 (-0.017, 0.585)

	Befriending n=66	Feeling Safe n=64	Adjusted Difference* (SE); p-value (95% CI)	*Cohen d* (95% CI)
	Unadjusted mean (SD)	Unadjusted mean (SD)		
**BCSS Self Negative**				
Baseline	11.98 (5.77)	11.81 (5.31)	-	-
6 months	9.11 (5.79)	7.78 (5.84)	1.628 (0.783); p=0.038 (0.094, 3.163)	0.295 (0.017, 0.572)
12 months	9.53 (6.27)	8.46 (5.84)	1.150 (0.802); p=0.151 (-0.421, 2.721)	0.208 (-0.076, 0.492)
**BCSS Self Positive**				
Baseline	7.35 (4.65)	7.84 (5.17)	-	-
6 months	8.43 (5.15)	9.93 (5.53)	1.256 (0.737); p=0.088 (0.188, 2.700)	0.256 (-0.038, 0.551)
12 months	9.09 (4.73)	9.19 (6.23)	0.477 (0.755); p=0.527 (-1.958, 1.003)	-0.097 (-0.400, 0.205)
**BCSS Others Negative**				
Baseline	13.89 (5.33)	14.34 (5.15)	-	-
6 months	9.74 (6.39)	8.87 (6.40)	1.302 (0.786); p=0.097 (-0.238, 2.842)	0.249 (-0.045, 0.544)
12 months	11.32 (6.17)	9.08 (6.56)	2.339 (0.797); p=0.003 (0.777, 3.901)	0.447 (0.148, 0.746)
**BCSS Others Positive**				
Baseline	9.60 (4.14)	9.11 (4.90)	-	-
6 months	10.05 (4.17)	12.20 (5.96)	2.242 (0.712); p=0.002 (0.846, 3.638)	0.497 (0.187, 0.806)
12 months	9.98 (4.41)	11.15 (5.40)	0.982 (0.725); p=0.176 (-0.440, 2.403)	0.217 (-0.097, 0.532)
**Insomnia**				
Baseline	14.02 (6.36)	13.52 (7.4)	-	-

	Befriending n=66<br>Unadjusted mean (SD)	Feeling Safe n=64<br>Unadjusted mean (SD)	Adjusted Difference* (SE); p-value (95% CI)	*Cohen d* (95% CI)
6 months	12.25 (6.81)	8.5 (6.21)	2.983 (0.967); p=0.002 (1.090, 4.876)	0.433 (0.158, 0.709)
12 months	13.32 (6.60)	10.17 (6.78)	2.024 (0.969); p=0.037 (0.125, 3.923)	0.294 (0.018, 0.570)
**Safety behaviours**				
Baseline	34.14 (16.58)	33.85 (16.91)	-	-
6 months	21.98 (16.54)	19.85 (18.49)	2.221 (2.401); p=0.355 (-2.484, 6.926)	0.133 (-0.149, 0.415)
12 months	20.78 (15.46)	18.73 (13.35)	1.414 (2.515); p=0.574 (-3.515, 6.342)	0.085 (-0.211, 0.380)
**Jumping to conclusions**				
Baseline	4.14 (4.47)	3.50 (3.2)	-	-
6 months	4.98 (4.62)	3.57 (3.34)	1.124 (0.550); p=0.041 (0.046, 2.203)	0.285 (0.012, 0.568)
12 months	4.49 (3.91)	4.08 (3.26)	0.186 (0.585); p=0.750, (-0.960, 1.332)	0.047 (-0.243, 0.338)
**Possibility of being mistaken**				
Baseline	19.92 (20.90)	19.20 (21.21)	-	-
6 months	36.80 (28.07)	48.11 (34.65)	13.925 (4.468); p=0.002 (5.169, 22.681)	0.664 (0.246, 1.081)
12 months	35.05 (30.76)	47.16 (33.98)	12.532 (4.523); p=0.006 (3.668, 21.396)	0.597 (0.175, 1.020)
**Anomalous experiences**				
Baseline	21.80 (15.02)	22.38 (14.63)	-	-
6 months	15.57 (12.87)	16.34 (14.93)	0.755 (1.457); p=0.604 (-2.101, 3.611)	0.051 (-0.142, 0.244)
12 months	14.37 (12.97)	19.16 (15.61)	-2.021 (1.477); p=0.171 (-4.916, 0.875)	-0.137 (-0.333, 0.059)

Table 13: Mediation effects of Feeling Safe. Mediator variables at 6 months and conviction (binary) at 6 months. Effects show: causal mediation effect (percentile bootstrap SE); 95% confidence interval.

**Mediator**	**Total effect**	**Direct effect**	**Indirect effect**	**Proportion mediated**
Safety beliefs	2.27 (1.56)<br>0.73, 5.62	1.73 (0.96)<br>0.71, 4.10	1.31 (0.35)<br>0.87, 2.25	42.5%
Vulnerability	2.26 (1.66)<br>0.63, 6.05	1.14 (0.64)<br>0.39, 2.53	1.99 (0.71)<br>1.19, 3.90	**89.0%**
Worry (PSWQ)	3.03 (1.88)<br>1.05, 7.02	2.21 (1.19)<br>0.85, 4.83	1.38 (0.39)<br>0.94, 2.50	41.0%
BCSS Self Negative	2.42 (1.45)<br>0.83, 5.50	1.77 (0.97)<br>0.64, 3.94	1.37 (0.33)<br>0.95, 2.21	46.0%
BCSS Self Positive	2.26 (1.13)<br>0.90, 4.60	1.97 (0.97)<br>0.74, 4.17	1.15 (0.15)<br>0.98, 1.63	23.4%
BCSS Others Negative	2.62 (1.64)<br>0.91, 6.04	2.10 (1.20)<br>0.80, 4.78	1.25 (0.25)<br>0.91, 2.02	32.3%
BCSS Others Positive	2.25 (1.17)<br>0.83, 4.68	1.66 (0.82)<br>0.62, 3.47	1.35 (0.24)<br>1.05, 2.06	**46.8%**
Insomnia (ISI)	2.25 (1.12)<br>0.08, 4.48	1.87 (0.92)<br>0.66, 3.84	1.20 (0.22)<br>0.98, 2.05	30.1%
Safety Behaviours	2.89 (1.74)<br>1.03, 6.42	2.55 (1.36)<br>0.99, 5.52	1.13 (0.29)<br>0.84, 1.91	17.6%
Jumping to conclusions	3.25 (2.05)<br>1.15, 6.88	3.38 (2.40)<br>1.23, 7.31	0.96 (0.10)<br>0.69, 1.14	-6.0%
Jumping to conclusions (binary)	3.15 (2.16)<br>1.23, 7.37	3.70 (2.89)<br>1.25, 9.39	0.85 (0.14)<br>0.45, 1.03	-25.9%
Possibility of being mistaken (MADS)	2.30 (2.10)<br>0.71, 5.71	1.25 (0.66)<br>0.40, 2.87	1.83 (0.80)<br>1.08, 3.82	**80.6%**
Anomalous experiences (SPEQ)	1.86 (0.98)<br>0.69, 3.80	1.78 (0.8)<br>0.67, 3.52	1.05 (0.18)<br>0.83, 1.59	10.2%

Table 14: Mediation effects of Feeling Safe. Mediator variables at 6 months and conviction (binary) at 12 months. Effects show: causal mediation effect (percentile bootstrap SE); 95% confidence interval.

**Mediator**	**Total effect**	**Direct effect**	**Indirect effect**	**Proportion mediated**
Safety beliefs	1.65 (1.16)<br>0.76, 5.97	1.36 (0.75)<br>0.60, 3.46	1.22 (0.26)<br>0.84, 1.83	45.4%
Vulnerability	1.60 (0.92)<br>0.73, 4.82	1.09 (0.60)<br>0.46, 2.88	1.47 (0.35)<br>1.05, 2.39	**85.1%**
Worry (PSWQ)	1.62 (0.91)<br>0.68, 4.44	1.35 (0.71)<br>0.54, 3.55	1.20 (0.22)<br>1.00, 1.94	43.5%
BCSS Self Negative	1.56 (0.92)<br>0.66, 5.10	1.17 (0.63)<br>0.45, 2.84	1.33 (0.27)<br>0.99, 2.19	69.4%
BCSS Self Positive	1.51 (0.79)	1.39 (0.72)	1.08 (0.10)	22.2%

	0.71, 4.07	0.62, 3.41	0.98, 1.50	
BCSS Others Negative	1.65 (1.02)<br>0.67, 4.79	1.35 (0.73)<br>0.54, 3.28	1.23 (0.25)<br>0.87, 1.99	47.0%
BCSS Others Positive	1.47 (0.77)<br>0.68, 3.57	1.23 (0.66)<br>0.52, 3.03	1.19 (0.15)<br>1.01, 1.65	**50.4%**
Insomnia (ISI)	1.57 (0.83)<br>0.68, 4.37	1.26 (0.65)<br>0.52, 3.00	1.24 (0.20)<br>1.02, 1.87	**53.8%**
Safety Behaviours	1.49 (0.80)<br>0.66, 3.91	1.43 (0.75)<br>0.63, 3.81	1.04 (0.10)<br>0.95, 1.36	11.7%
Jumping to conclusions	1.74 (1.03)<br>0.73, 4.85	1.91 (1.13)<br>0.76, 4.97	0.91 (0.10)<br>0.66, 1.04	-23.3%
Jumping to conclusions (binary)	1.81 (1.36)<br>0.62, 5.48	2.37 (1.57)<br>0.98, 7.18	0.76 (0.16)<br>0.46, 1.08	-71.0%
Possibility of being mistaken (MADS)	1.52 (1.39)<br>0.58, 5.59	0.79 (0.44)<br>0.32, 2.25	1.92 (0.83)<br>1.00, 3.28	140.6%
Anomalous experiences (SPEQ)	1.29 (0.67)<br>0.60, 3.39	1.25 (0.64)<br>0.55, 3.03	1.03 (0.10)<br>0.89, 1.30	13.0%

Table 15: Mediation effects of Feeling Safe. Mediator variables at 6 months and conviction (continuous) at 6 months. Effects show: causal mediation effect (percentile bootstrap SE); 95% confidence interval.

Mediator	Total effect	Direct effect	Indirect effect	Proportion mediated
Safety beliefs	-9.97 (5.77)<br>-20.56, 1.42	-6.11 (4.79)<br>-15.14, 3.92	-3.86 (3.20)<br>-10.64, 1.86	38.7%
Vulnerability	-8.75 (5.83)<br>-18.86, 3.59	-020 (4.91)<br>-9.40, 9.86	-8.55 (3.51)<br>-16.68, -2.55	**97.7%**
Worry (PSWQ)	-15.37 (6.00)<br>-25.66, -2.12	-11.22 (5.53)<br>-21.05, 0.22	-4.16 (2.83)<br>-11.10, 0.30	27.1%
BCSS Self Negative	-12.69 (5.84)<br>-23.21, 0.62	-8.40 (5.57)<br>-19.38, 2.78	-4.29 (2.63)<br>-9.93, 0.08	33.8%
BCSS Self Positive	-13.2 (5.83)<br>-23.72, -0.36	-11.10 (5.77)<br>-21.81, 0.77	-2.17 (1.91)<br>-7.92, 0.10	16.4%
BCSS Others Negative	-13.74 (5.70)<br>-24.56, -2.64	-10.24 (5.28)<br>-20.34, 0.48	-3.50 (2.68)<br>-9.09, 1.25	25.5%
BCSS Others Positive	-13.09 (5.83)<br>-23.55, -0.87	-7.70 (5.78)<br>-17.86, 4.93	-5.38 (2.35)<br>-11.41, -1.40	**41.1%**
Insomnia (ISI)	-15.03 (6.24)<br>-25.37, -1.54	-12.80 (6.37)<br>-23.02, 1.87	-2.23 (2.00)<br>-8.19, 0.63	14.8%
Safety Behaviours	-15.31 (5.96)<br>-25.30, -2.50	-13.59 (5.71)<br>-24.46, -1.27	-1.72 (2.66)<br>-9.05, 2.28	11.2%
Jumping to conclusions	-15.55 (6.50)<br>-26.22, -1.01	-15.45 (6.39)<br>-26.07, -1.30	-0.10 (1.61)<br>-3.21, 3.13	0.6%
Jumping to conclusions (binary)	-14.58 (6.75)<br>-25.75, 0.11	-16.77 (6.41)<br>-27.49, -1.54	2.18 (3.01)<br>-0.52, 12.44	-15.0%
Possibility of being mistaken (MADS)	-10.24 (5.74)<br>-20.32, 1.19	-0.60, (4.18)<br>-8.64, 7.97	-9.64 (4.31)<br>-18.23, -1.63	**94.1%**
Anomalous experiences (SPEQ)	-9.68 (5.90)<br>-19.44, 3.49	-8.97 (5.80)<br>-19.09, 2.83	-0.71 (2.25)<br>-6.43, 2.86	7.3%

Table 16: Mediation effects of Feeling Safe. Mediator variables at 6 months and conviction (continuous) at 12 months. Effects show: causal mediation effect (percentile bootstrap SE); 95% confidence interval.

Mediator	Total effect	Direct effect	Indirect effect	Proportion mediated
Safety beliefs	-9.84 (6.35)<br>-23.79, 1.70	-6.55 (5.87)<br>-18.72, 4.00	-3.30 (2.98)<br>-9.28, 3.14	33.5%
Vulnerability	-8.41 (6.55)<br>-21.74, 3.77	-1.12 (6.35)<br>-14.25, 10.51	-7.29 (3.33)<br>-14.39, -1.17	**86.7%**
Worry (PSWQ)	-11.53 (7.03)<br>-26.90, 1.47	-8.14 (6.78)<br>-22.00, 4.61	-3.39 (2.59)<br>-11.93, -0.28	29.4%
BCSS Self Negative	-10.01 (6.97)<br>-24.57, 2.77	-5.5.50 (6.30)<br>-19.13, 6.22	-4.52 (2.88)<br>-10.84, -0.13	**45.2%**
BCSS Self Positive	-10.15 (6.81)<br>-24.77, 2.66	-8.45 (6.71)<br>-22.30, 5.44	-1.70 (1.50)<br>-6.96, 0.27	16.7%
BCSS Others Negative	-10.88 (6.70)<br>-25.13, 1.65	-7.36 (6.19)<br>-20.30, 4.51	-3.52 (2.76)<br>-9.21, 2.33	32.4%
BCSS Others Positive	-9.79 (6.80)<br>-23.97, 2.55	-5.92 (6.71)<br>-19.67, 7.13	-3.87 (6.80)<br>-9.39, -0.82	**39.5%**
Insomnia (ISI)	-10.86 (6.99)<br>-25.50, 1.63	-6.43 (6.83)<br>-21.85, 6.21	-4.42 (2.51)<br>-10.64, -0.87	**40.7%**
Safety Behaviours	-9.85 (7.04)<br>-25.20, 2.66	-8.93 (7.06)<br>-24.84, 4.11	-0.92 (1.73)<br>-5.78, 1.47	9.3%
Jumping to conclusions	-12.23 (7.44)<br>-26.75, 1.34	-13.50 (7.31)<br>-27.30, 0.40	1.28 (1.45)<br>-0.51, 5.52	10.5%
Jumping to conclusions (binary)	-10.80 (7.86)<br>-26.08, 3.60	-15.02 (7.08)<br>-29.65, -2.46	4.22 (3.53)<br>-0.68, 13.99	-39.1%
Possibility of being mistaken (MADS)	-7.98 (6.52)<br>-20.80, 4.08	1.86 (5.02)<br>-9.52, 10.28	-9.84 (4.17)<br>-16.75, -1.45	**123.3%**
Anomalous experiences (SPEQ)	-6.96 (6.66)<br>-21.89, 5.17	-6.28 (6.27)<br>-20.58, 4.74	-0.69 (2.08)<br>-5.11, 2.90	14.4%

Table 17: Moderator scoring

	Befriending n=66	Feeling Safe n=64	Overall n=130
**Working Memory**			
WAIS Letter-Number sequencing score	9.62 (11.81); 63	7.93 (3.07); 60	8.80 (8.73); 123
WAIS forward digit span score	9.44 (2.10); 63	9.38 (2.50); 60	9.41 (2.29); 123
WAIS backward digit span score	7.48 (11.91); 63	5.53 (1.68); 60	6.53 (8.63); 123
**Illicit drug use n(%)**			
Any	16 (24.24%)	5 (7.94%)	21 (16.28%)
Other than cannabis/skunk	10 (15.15%)	2 (3.23%)	12 (9.38%)
**Anger**	10.74 (4.51); 65	11.06 (4.82); 62	10.90 (4.65); 127
**Presence of voices , n(%)**	43 (66.15%)	40 (62.50%)	83 (64.34%)

Data presented as mean (SD); n, or n (%)

Table 18: Moderation analysis results

**Moderator**		**Outcome**	
		Conviction (binary)	Conviction (cont.)
**Working Memory (WAIS Letter-Number)**			
	6 Months	1.105 (0.684, 1.787); p=0.683	1.188 (-1.027, 3.403); p=0.293
	12 Months	0.697 (0.452, 1.310); p=0.335	0.468 (-1.882, 2.818); p=0.696
**Working Memory (Forward digit span)**			
	6 Months	0.909 (0.479, 1.722); p=0.769	-0.539 (-4.726, 3.648); p=0.801
	12 Months	0.726 (0.376, 1.399); p=0.338	-0.239 (-4.480, 4.003); p=0.912
**Working Memory (Backward digit span)**			
	6 Months	0.817 (0.362, 1.841); p=0.625	-0.851 (-4.844, 3.141); p=0.676
	12 Months	0.476 (0.194, 1.165); p=0.104	-4.515 (-8.672, -0.358); p=0.033

Moderator		Outcome: Conviction (binary)	Outcome: Conviction (cont.)
**Illicit drug use - Any**			
	6 Months	6.980 (0.084, 583.221); p=0.389	-7.196 (-35.720, 21.328); p=0.621
	12 Months	0.828 (0.011, 63.090); p=0.932	-23.276 (-52.087, 5.534); p=0.113
**Illicit drug use - Other than cannabis/skunk**			
	6 Months	-	11.007 (-30.501, 52.516); p=0.603
	12 Months	-	-27.175 (-69.121, 14.771); p=0.204
**Anger**			
	6 Months	0.926 (0.665, 1.290); p=0.650	-0.972 (-3.059, 1.115); p=0.361
	12 Months	0.864 (0.620, 1.202); p=0.385	-1.847 (-3.962, 0.267); p=0.087
**Presence of voices**			
	6 Months	0.529 (0.021, 13.295); p=0.699	6.541 (-12.731, 25.814); p=0.506
	12 Months	1.767 (0.074, 41.950); p=0.725	12.722 (-7.047, 32.490); p=0.207

Additional analyses:

Table 19: Time in therapy by allocation

	**Befriending n=66**	**Feeling Safe n=64**	**Total n=130**
Total time in mins, mean (SD)	906.4 (352.6)	1195.2 (464.1)	1048.6 (434.6)
Total sessions, mean (SD)	16.4 (5.7)	19.2 (6.8)	17.7 (6.4)

Table 20: Primary outcome analysis adjusted for therapy time (mins) - Conviction (cont.)

	**Befriending n=66** Unadjusted mean (SD)	**Feeling Safe n=64** Unadjusted mean (SD)	**Adjusted Difference* (SE); p-value (95% CI)**	***Cohen d* (95% CI)**
**Conviction**				
Baseline	86.4 (12.6)	87.1 (12.2)	-	-
6 months	59.6 (27.1)	49.4 (35.5)	9.407 (4.812); p=0.051 (-0.026, 18.840)	0.760 (0.002, 1.523)
12 months	59.4 (32.8)	50.2 (36.0)	7.113 (4.911); p=0.15 (-2.513, 16.739)	0.575 (-0.203, 1.353)

Adjusted for baseline score, therapist, time spent in therapy (mins) and interaction of timepoint with treatment allocation, and including a random effect at the individual level

Table 21: Primary outcome analysis adjusted for therapy time (sessions) - Conviction (cont.)

	**Befriending n=66**<br>Unadjusted mean (SD)	**Feeling Safe n=64**<br>Unadjusted mean (SD)	**Adjusted Difference* (SE); p-value (95% CI)**	***Cohen d* (95% CI)**
**Conviction**				
Baseline	86.4 (12.6)	87.1 (12.2)	-	-
6 months	59.6 (27.1)	49.4 (35.5)	10.500 (4.698); p=0.025 (1.293, 19.707)	0.849 (0.104, 1.593)
12 months	59.4 (32.8)	50.2 (36.0)	8.232 (4.792); p=0.086 (-1.161, 17.624)	0.665 (-0.094, 1.424)

Adjusted for baseline score, therapist, time spent in therapy (no. of sessions) and interaction of timepoint with treatment allocation, and including a random effect at the individual level

Table 22: Primary outcome analysis adjusted for therapy time (mins) - Conviction (bin.)

	**Befriending n=66**<br>n (%)	**Feeling Safe n=64**<br>n (%)	**OR (SE); p-value (95% CI)**
**Conviction**			
Baseline	0 (0%)	0 (0%)	-
6 months	22 (34.9%)	32 (50.8%)	3.759 (1.96); p=0.091 (0.811-17.426)
12 months	22 (34.9%)	27 (46.6%)	2.250 (1.738); p=0.294 (0.495-10.228)

Adjusted for baseline score, therapist, time spent in therapy (mins) and interaction of timepoint with treatment allocation, and including a random effect at the individual level

Table 23: Primary outcome analysis adjusted for therapy time (sessions) - Conviction (bin.)

	**Befriending n=66**<br>n (%)	**Feeling Safe n=64**<br>n (%)	**OR (SE); p-value (95% CI)**
**Conviction**			
Baseline	0 (0%)	0 (0%)	-
6 months	22 (34.9%)	32 (50.8%)	4.020 (3.061); p=0.068 (0.904-17.878)
12 months	22 (34.9%)	27 (46.6%)	2.411 (1.803); p=0.240 (0.556-10.446)

Adjusted for baseline score, therapist, time spent in therapy (no. of sessions) and interaction of timepoint with treatment allocation, and including a random effect at the individual level

Table 24: Secondary outcome (PSYRATS) analysis adjusted for therapy time (mins)

	**Befriending n=66** Unadjusted mean (SD)	**Feeling Safe n=64** Unadjusted mean (SD)	**Adjusted Difference* (SE); p-value (95% CI)**	***Cohen d* (95% CI)**
**PSYRATS**				
Baseline	18.2 (2.6)	18.5 (2.3)	-	-
6 months	14.2 (4.8)	11.6 (5.9)	2.791 (0.871); p=0.001 (1.083, 4.498)	1.133 (0.440, 1.826)
12 months	13.5 (5.6)	11.6 (6.4)	1.9975 (0.890); p=0.026 (0.231, 3.720)	0.802 (0.094, 1.510)

**Adjusted for baseline score, therapist, time spent in therapy (mins) and interaction of timepoint with treatment allocation, and including a random effect at the individual level*

Table 25: Secondary outcome (PSYRATS) analysis adjusted for therapy time (sessions)

	**Befriending n=66** Unadjusted mean (SD)	**Feeling Safe n=64** Unadjusted mean (SD)	**Adjusted Difference* (SE); p-value (95% CI)**	***Cohen d* (95% CI)**
**PSYRATS**				
Baseline	18.2 (2.6)	18.5 (2.3)	-	-
6 months	14.2 (4.8)	11.6 (5.9)	2.906 (0.849); p=0.001 (1.243, 4.570)	1.180 (-0.504, 1.855)
12 months	13.5 (5.6)	11.6 (6.4)	2.095 (0.866); p=0.016 (0.398, 3.793)	0.851 (0.161, 1.540)

**Adjusted for baseline score, therapist, time spent in therapy (no. of sessions) and interaction of timepoint with treatment allocation, and including a random effect at the individual level*

Table 26: Expectancy/Credibility scores by allocation

	**Befriending n=61**	**Feeling Safe n=58**	**Total n=130**
Expectancy score, mean (SD)	17.96 (5.0)*	19.8 (5.0)	18.8 (5.1)
Credibility score, mean (SD)	20 (5.0)	21.2 (4.4)	20.6 (7.8)

**Missing: n=2*

Table 27: Primary outcome analysis adjusted for expectancy scores - Conviction (cont.)

	**Befriending n=66**	**Feeling Safe n=64**	**Adjusted Difference* (SE); p-value (95% CI)**	***Cohen d* (95% CI)**

	Unadjusted mean (SD)	Unadjusted mean (SD)		
**Conviction**				
Baseline	86.4 (12.6)	87.1 (12.2)	-	-
6 months	59.6 (27.1)	49.4 (35.5)	9.267 (4.623); p=0.045 (0.207, 18.327)	0.749 (0.016, 1.481)
12 months	59.4 (32.8)	50.2 (36.0)	7.653 (4.722); p=0.105 (-1.602, 16.908)	0.618 (-0.129, 1.366)

**Adjusted for baseline score, therapist, expectancy score and interaction of timepoint with treatment allocation, and including a random effect at the individual level*

Table 28: Primary outcome analysis adjusted for credibility scores - Conviction (cont.)

	**Befriending n=66**<br>Unadjusted mean (SD)	**Feeling Safe n=64**<br>Unadjusted mean (SD)	**Adjusted Difference* (SE); p-value (95% CI)**	***Cohen d* (95% CI)**
**Conviction**				
Baseline	86.4 (12.6)	87.1 (12.2)	-	-
6 months	59.6 (27.1)	49.4 (35.5)	8.232 (4.652); p=0.077 (-0.885, 17.350)	0.665 (-0.715, 1.402)
12 months	59.4 (32.8)	50.2 (36.0)	5.339 (4.768); p=0.263 (-4.007, 14.685)	0.432 (-0.324, 1.187)

**Adjusted for baseline score, therapist, credibility score and interaction of timepoint with treatment allocation, and including a random effect at the individual level*

Table 29: Primary outcome analysis adjusted for expectancy scores - Conviction (bin.)

	**Befriending n=66**<br>n (%)	**Feeling Safe n=64**<br>n (%)	**OR (SE); p-value (95% CI)**
**Conviction**			
Baseline	0 (0%)	0 (0%)	-
6 months	22 (34.9%)	32 (50.8%)	2.650 (2.005); p=0.197 (0.602-11.671)
12 months	22 (34.9%)	27 (46.6%)	1.753 (1.329); p=0.459 (0.397-7.747)

*Adjusted for baseline score, therapist, expectancy score and interaction of timepoint with treatment allocation, and including a random effect at the individual level

Table 30: Primary outcome analysis adjusted for credibility scores - Conviction (bin.)

	**Befriending n=66** n (%)	**Feeling Safe n=64** n (%)	**OR (SE); p-value (95% CI)**
**Conviction**			
Baseline	0 (0%)	0 (0%)	-
6 months	22 (34.9%)	32 (50.8%)	2.730 (2.039); p=0.179 (0.632-11.800)
12 months	22 (34.9%)	27 (46.6%)	1.522 (1.138); p=0.574 (0.352-6.587)

*Adjusted for baseline score, therapist, credibility score and interaction of timepoint with treatment allocation, and including a random effect at the individual level

Table 31: Primary outcome analysis adjusted for antipsychotic CPZ equivalent dose - Conviction (cont.)

	**Befriending n=66** Unadjusted mean (SD)	**Feeling Safe n=64** Unadjusted mean (SD)	**Adjusted Difference* (SE); p-value (95% CI)**	***Cohen d* (95% CI)**
**Conviction**				
Baseline	86.4 (12.6)	87.1 (12.2)	-	-
6 months	59.6 (27.1)	49.4 (35.5)	11.843 (4.741); p=0.012 (2.551, 21.134)	0.957 (0.206, 1.708)
12 months	59.4 (32.8)	50.2 (36.0)	12.413 (4.942); p=0.012 (2.726, 22.100)	1.003 (0.220, 1.786)

*Adjusted for baseline score, therapist, antipsychotic CPZ equivalent dose at outcome timepoint and interaction of timepoint with treatment allocation, and including a random effect at the individual level

Table 32: Primary outcome analysis adjusted for antipsychotic CPZ equivalent dose - Conviction (bin.)

	**Befriending n=66** n (%)	**Feeling Safe n=64** n (%)	**OR (SE); p-value (95% CI)**
**Conviction**			
Baseline	0 (0%)	0 (0%)	-
6 months	22 (34.9%)	32 (50.8%)	3.878 (2.840); p=0.064 (0.923-16.289)
12 months	22 (34.9%)	27 (46.6%)	2.809 (1.356); p=0.426 (0.419-7.853)

Adjusted for baseline score, therapist, antipsychotic CPZ equivalent dose at outcome timepoint and interaction of timepoint with treatment allocation, and including a random effect at the individual level

Table 33: Mediation effects of Feeling Safe. Mediator (conviction (continuous)) at 6 months and secondary outcomes at 6 months. Effects show: causal mediation effect (percentile bootstrap SE); 95% confidence interval.

Mediator – conviction cont.	Total effect	Direct effect	Indirect effect	Proportion mediated
Vulnerability	-15.16 (5.18)<br>-27.51, -6.68	-9.22 (4.39)<br>-18.71, -1.18	-5.94 (3.34)<br>-13.16, -0.32	**39.2%**
BCSS Others Positive	2.02 (0.87)<br>0.20, 3.44	1.27 (0.80)<br>-0.22, 2.81	0.74 (0.46)<br>0.05, 1.67	**36.6%**
Possibility of being mistaken (MADS)	13.29 (4.84)<br>4.76, 24.19	6.24 (3.66)<br>-0.68, 13.76	7.05 (3.59)<br>0.67, 14.85	**53.0%**

Table 34: Feeling safe modules and changes in module target

Change in module score	Befriending	Feeling Safe - Module not taken	Feeling Safe – Module taken	Total
Worry			n=28	
Baseline score	63.26 (11.27)	60.2 (12.21)	65.67 (7.42)	
Change at 6 months	-5.59 (9.52)	-7.65 (13.58)	-10.64 (13.72)	-7.23 (11.67)
Change at 12 months	-5.54 (10.14)	-7.86 (13.68)	-9.52 (9.95)	-6.99 (11.14)
Sleep			n=20	
Baseline score	14.02 (6.36)	13.57 (8.42)	13.46 (6.02)	
Change at 6 months	-1.51 (6.00)	-2.17 (5.29)	-8.57 (7.65)	-2.63 (6.39)
Change at 12 months	-0.70 (7.09)	-0.33 (6.72)	-6.93 (8.33)	-1.44 (7.42)

Voices			n=21	
Baseline score	21.8 (15.02)	22.53 (13.59)	22.19=8 (16.13)	
Change at 6 months	-5.65 (9.63)	-6.39 (11.05)	-7.14 (7.70)	-6.15 (9.75)
Change at 12 months	-6.20 (11.48)	-5.47 (11.85)	-3.71 (8.72)	-5.53 (11.10)
Safe enough			n=56	
Baseline score	37.88 (29.64)	38.33 (27.18)	37.00 (25.70)	
Change at 6 months	11.95 (29.57)	10.75 (37.50)	19.74 (36.39)	15.22 (33.14)
Change at 12 months	11.48 (33.03)	9.5 (37.31)	21.06 (41.30)	15.29 (36.93)
Self-confidence			n=32	
Baseline score	11.98 (5.77)	11.2 (5.58)	12.59 (4.92)	
Change at 6 months	-2.48 (5.72)	-4.46 (5.53)	-4.07 (5.25)	-3.32 (5.59)
Change at 12 months	-2.14 (5.79)	-3.70 (6.40)	-3.32 (4.84)	-2.78 (5.69)

Data presented as mean (SD)

Table 35: Standard care by allocation

	Befriending events(people)	Feeling Safe events(people)	Overall events(people)
Any other therapy – 6 months	7 (3)	6 (2)	13 (5)
CBT/ACT	1 (1)	0	1 (1)
1:1 psychology on ward	3 (2)	1 (2)	4 (4)
Ward group	0	1 (1)	1 (1)
Psychosis group	3 (1)	0	3 (1)
Mentalization group	0	1 (1)	1 (1)
Any other therapy – 12 months	45 (7)	19 (2)	64 (9)
CBT for psychosis	18 (3)	0	18 (3)
CBT (eating disorders)	21 (1)	0	21 (1)
Psychological therapy (managing suicidality)	0	12 (1)	12 (1)
1:1 psychology on ward	0	4 (1)	4 (1)
Ward group	1 (1)	3 (1)	4 (2)
Managing difficult emotions group	1 (1)	0	1 (1)
Pathfinders (using CBT strategies)	4 (1)	0	4 (1)

Table 36: Service use by allocation

	Feeling Safe Programme (Treatment group)		Befriending (Control group)	
	n	Mean (SD)	n	Mean (SD)
**6 months before the trial**				
Number of nights in hospital	64 (n=8 admissions)	4.58 (14.66)	66 (n=9 admissions)	8.42 (25.63)
Meetings with psychiatrist	60	1.83 (1.50)	63	2.22 (2.45)
Meetings with care coordinator (CPN or social worker)	62	9.20 (8.44)	62	9.73 (9.48)
Meetings with counsellor or therapist	62	0.81 (3.46)	63	2.14 (6.31)
Visits to day-care centre / day hospital	64	1.59 (4.94)	66	1.24 (3.35)
GP meetings	62	2.79 (3.03)	63	2.79 (3.61)
**During trial participation: Baseline to 6 month (post-treatment) assessment**				
Number of nights in hospital	64 (n=4 admissions)	2.05 (9.37)	64 (n=6 admissions)	4.87 (20.44)
Meetings with psychiatrist	47	1.26 (1.34)	53	1.85 (1.96)
Meetings with care coordinator (CPN or social worker)	45	4.36 (4.29)	53	6.64 (5.39)

Meetings with counsellor or therapist	46	0.87 (2.85)	53	0.13 (0.52)
Visits to day-care centre / day hospital	64	1.14 (3.80)	64	0.34 (1.3)
GP meetings	47	2.43 (3.93)	54	2.22 (2.92)
**During trial participation: 6 month to 12 month (follow-up) assessment**				
Number of nights in hospital	63 (n=7 admissions)	5.17 (20.21)	63 (n=7 admissions)	4.22 (13.30)
Meetings with psychiatrist	44	1.18 (1.17)	51	1.59 (1.73)
Meetings with care coordinator (CPN or social worker)	44	5.34 (6.94)	50	6.30 (5.99)
Meetings with counsellor or therapist	44	0.34 (1.82)	51	1.8 (4.43)
Visits to day-care centre / day hospital	63	0.48 (2.26)	63	0.78 (3.67)
GP meetings	43	1.53 (1.64)	51	2.73 (3.67)

Plots

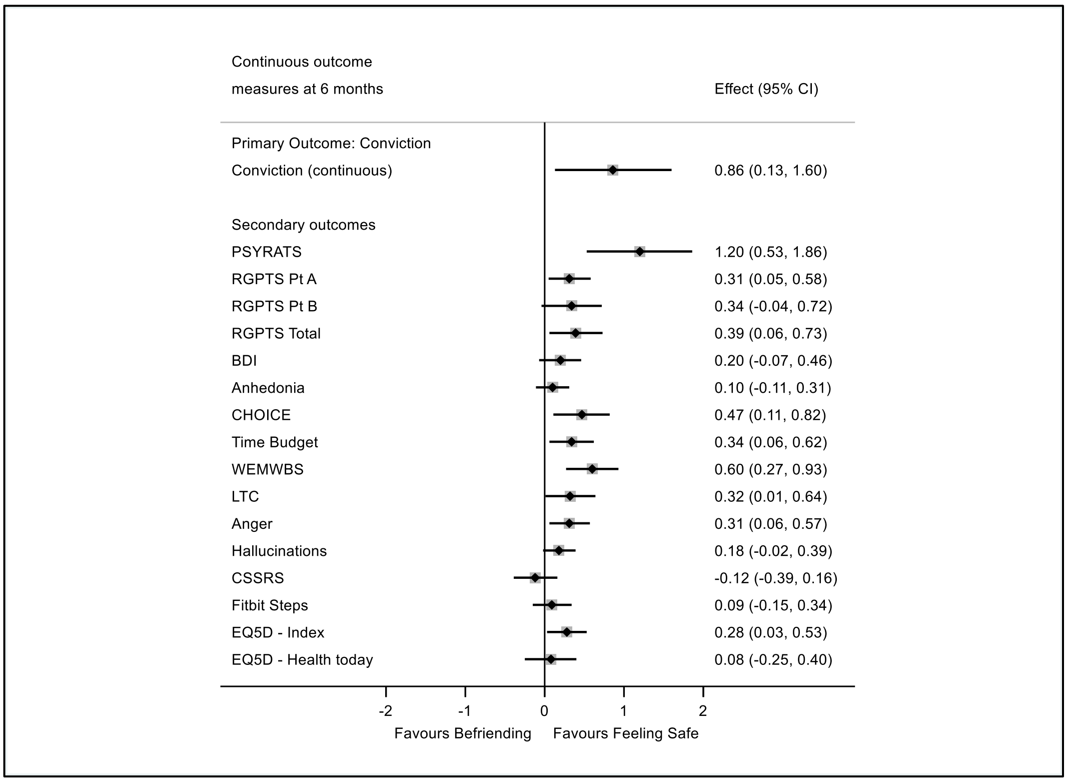

Figure 1: Continuous outcome measures at 6 months (effect size and 95% CI)

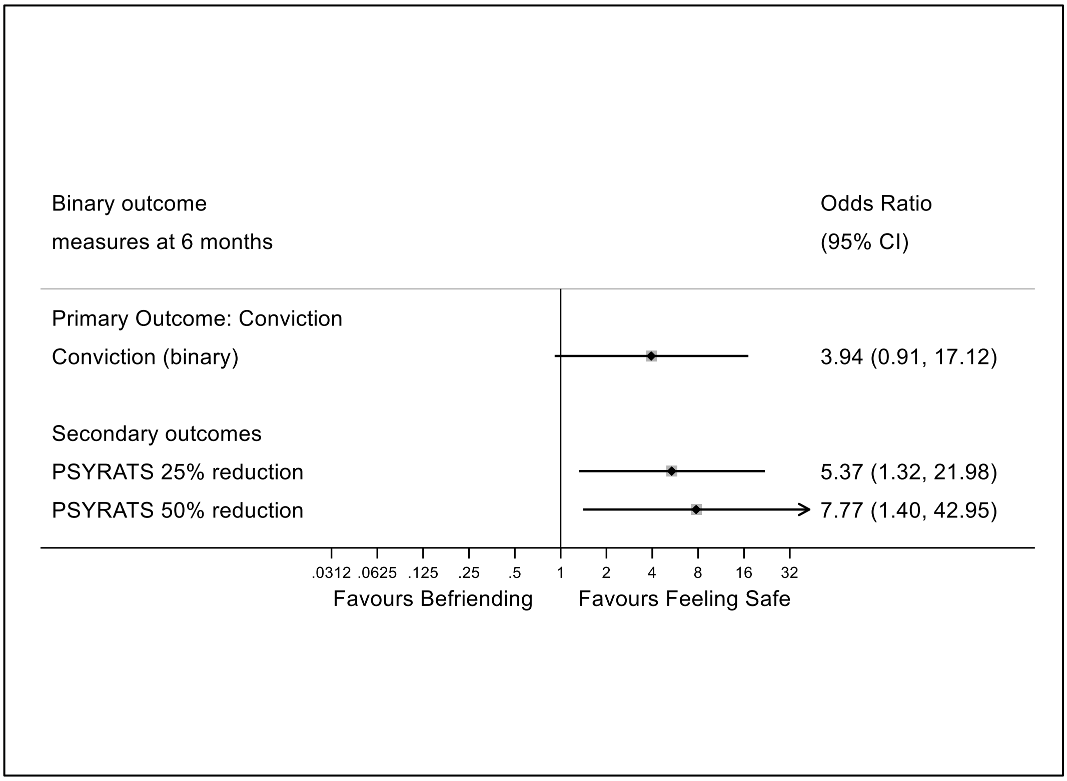

Figure 2: Binary outcome measures at 6 months (effect size and 95% CI)

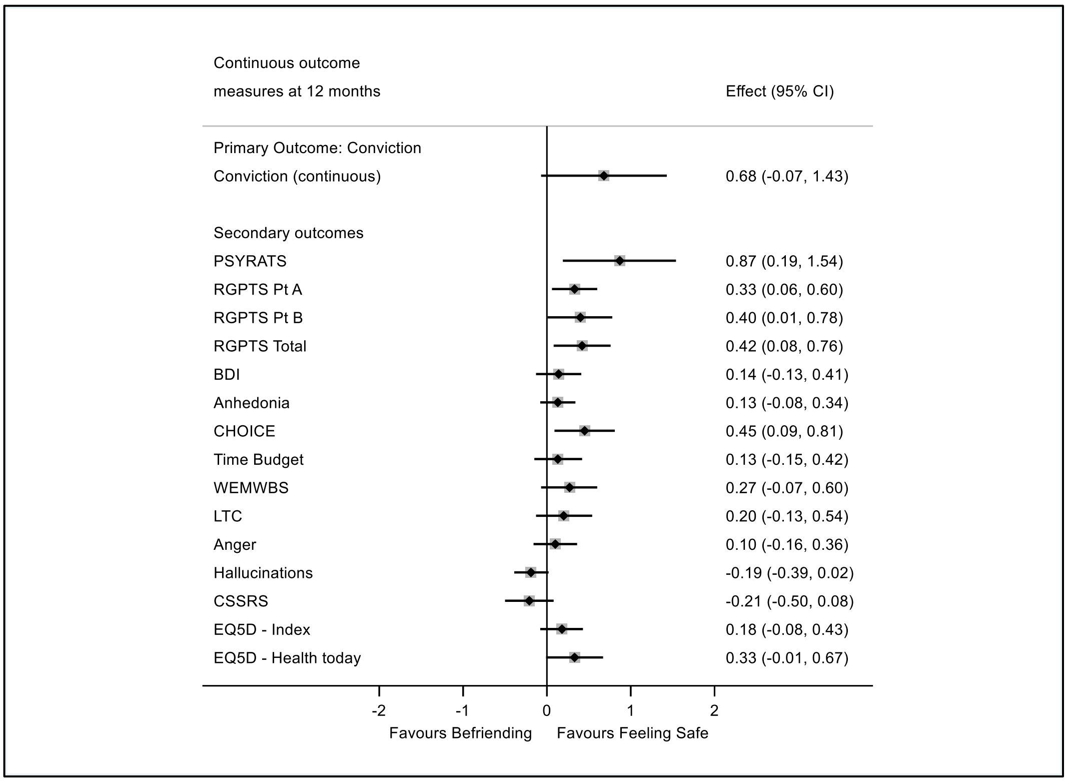

Figure 3: Continuous outcome measures at 12 months (effect size and 95% CI)

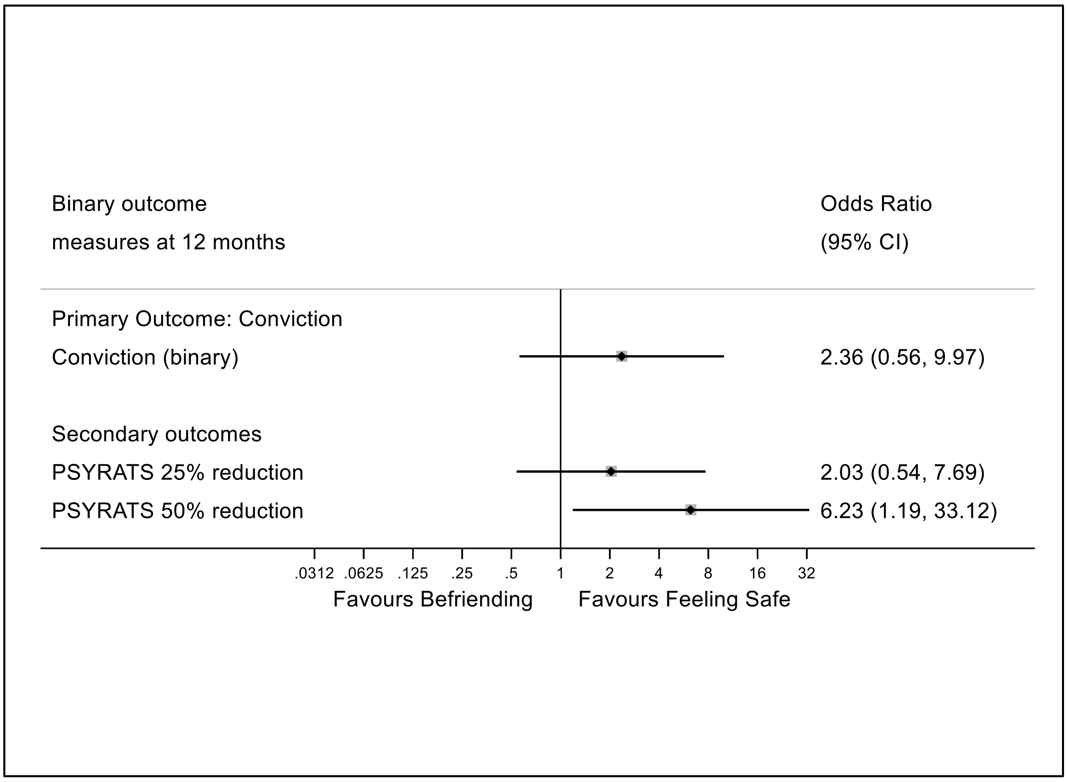

Figure 4: Binary outcome measures at 12 months (effect size and 95% CI)

Changes to protocol

The plan for analysis was finalised in the statistical analysis report (pages Appendix 27-42). All changes to the protocol were agreed before any outcome analyses were conducted, and were also agreed by the independent Trial Steering Committee (TSC) and Data Monitoring and Ethics Committee (DMEC). This was all signed off by the committees in June 2020 and the analysis report and main paper follow this plan.

In the protocol, belief flexibility was erroneously listed as an outcome but it is clearly a mediator (under reasoning) and moved to that category in the statistical analysis plan. The beliefs about safety and vulnerability and the anomalous experiences measure were explicitly added as mediators at this stage, since they are key mechanisms in the theoretical model being tested, which is why they were included in the study. The EQ-5D was also introduced as an outcome in the statistical analysis plan, since this is the most commonly used quality of life scale and there would be interest in the finding. With regard to moderators, the paper reports the three in the published trial protocol. The final change to the protocol was that we used the new revised method of scoring the GPTS, since it is an improvement to the scale (based upon an analysis of 10,000 people) and hence enables greater precision. The revised scale is reported in Freeman et al (2021).

Freeman, D., Loe, B.S., Kingdon, D., Startup, H., Molodynski, A., Rosebrock, L., Brown, P., Sheaves, B., Waite, F., & Bird, J.C. (2021). The revised Green et al., Paranoid Thoughts Scale (R-GPTS): psychometric properties, severity ranges, and clinical cut-offs. Psychological Medicine, 51, 244-253.

CONSORT 2010 checklist of information to include when reporting a randomised trial*

Section/Topic	Item No	Checklist item	Reported on page No
**Title and abstract**			
	1a	Identification as a randomised trial in the title	1
	1b	Structured summary of trial design, methods, results, and conclusions (for specific guidance see CONSORT for abstracts)	2
**Introduction**			
Background and objectives	2a	Scientific background and explanation of rationale	4
	2b	Specific objectives or hypotheses	5
**Methods**			
Trial design	3a	Description of trial design (such as parallel, factorial) including allocation ratio	5
	3b	Important changes to methods after trial commencement (such as eligibility criteria), with reasons	n/a
Participants	4a	Eligibility criteria for participants	5
	4b	Settings and locations where the data were collected	5
Interventions	5	The interventions for each group with sufficient details to allow replication, including how and when they were actually administered	7-8
Outcomes	6a	Completely defined pre-specified primary and secondary outcome measures, including how and when they were assessed	6
	6b	Any changes to trial outcomes after the trial commenced, with reasons	n/a
Sample size	7a	How sample size was determined	9
	7b	When applicable, explanation of any interim analyses and stopping guidelines	n/a
Randomisation:			
Sequence generation	8a	Method used to generate the random allocation sequence	6
	8b	Type of randomisation; details of any restriction (such as blocking and block size)	6
Allocation concealment mechanism	9	Mechanism used to implement the random allocation sequence (such as sequentially numbered containers), describing any steps taken to conceal the sequence until interventions were assigned	6
Implementation	10	Who generated the random allocation sequence, who enrolled participants, and who assigned participants to interventions	6

Blinding	11a	If done, who was blinded after assignment to interventions (for example, participants, care providers, those assessing outcomes) and how	6
	11b	If relevant, description of the similarity of interventions	7
Statistical methods	12a	Statistical methods used to compare groups for primary and secondary outcomes	8-9
	12b	Methods for additional analyses, such as subgroup analyses and adjusted analyses	9
**Results**			
Participant flow (a diagram is strongly recommended)	13a	For each group, the numbers of participants who were randomly assigned, received intended treatment, and were analysed for the primary outcome	11
	13b	For each group, losses and exclusions after randomisation, together with reasons	Figure 1
Recruitment	14a	Dates defining the periods of recruitment and follow-up	11
	14b	Why the trial ended or was stopped	11
Baseline data	15	A table showing baseline demographic and clinical characteristics for each group	Table 1
Numbers analysed	16	For each group, number of participants (denominator) included in each analysis and whether the analysis was by original assigned groups	Table 2
Outcomes and estimation	17a	For each primary and secondary outcome, results for each group, and the estimated effect size and its precision (such as 95% confidence interval)	Table 2 and page 12
	17b	For binary outcomes, presentation of both absolute and relative effect sizes is recommended	Table 2
Ancillary analyses	18	Results of any other analyses performed, including subgroup analyses and adjusted analyses, distinguishing pre-specified from exploratory	Appendix
Harms	19	All important harms or unintended effects in each group (for specific guidance see CONSORT for harms)	13, and appendix
**Discussion**			
Limitations	20	Trial limitations, addressing sources of potential bias, imprecision, and, if relevant, multiplicity of analyses	15
Generalisability	21	Generalisability (external validity, applicability) of the trial findings	15
Interpretation	22	Interpretation consistent with results, balancing benefits and harms, and considering other relevant evidence	14
**Other information**			
Registration	23	Registration number and name of trial registry	2 and 5
Protocol	24	Where the full trial protocol can be accessed, if available	5
Funding	25	Sources of funding and other support (such as supply of drugs), role of funders	10 & 18

*We strongly recommend reading this statement in conjunction with the CONSORT 2010 Explanation and Elaboration for important clarifications on all the items. If relevant, we also recommend reading CONSORT extensions for cluster randomised trials, non-inferiority and equivalence trials, non-pharmacological treatments, herbal interventions, and pragmatic trials. Additional extensions are forthcoming: for those and for up to date references relevant to this checklist, see www.consort-statement.org.
